# Evolutionary primacy of sodium bioenergetics

**DOI:** 10.1186/1745-6150-3-13

**Published:** 2008-04-01

**Authors:** Armen Y Mulkidjanian, Michael Y Galperin, Kira S Makarova, Yuri I Wolf, Eugene V Koonin

**Affiliations:** 1 School of Physics, University of Osnabrück, D-49069 Osnabrück, Germany; 2 A.N. Belozersky Institute of Physico-Chemical Biology, Moscow State University, Moscow, 119991, Russia; 3 National Center for Biotechnology Information, National Library of Medicine, National Institutes of Health, Bethesda, MD 20894, USA

## Abstract

**Background:**

The F- and V-type ATPases are rotary molecular machines that couple translocation of protons or sodium ions across the membrane to the synthesis or hydrolysis of ATP. Both the F-type (found in most bacteria and eukaryotic mitochondria and chloroplasts) and V-type (found in archaea, some bacteria, and eukaryotic vacuoles) ATPases can translocate either protons or sodium ions. The prevalent proton-dependent ATPases are generally viewed as the primary form of the enzyme whereas the sodium-translocating ATPases of some prokaryotes are usually construed as an exotic adaptation to survival in extreme environments.

**Results:**

We combine structural and phylogenetic analyses to clarify the evolutionary relation between the proton- and sodium-translocating ATPases. A comparison of the structures of the membrane-embedded oligomeric proteolipid rings of sodium-dependent F- and V-ATPases reveals nearly identical sets of amino acids involved in sodium binding. We show that the sodium-dependent ATPases are scattered among proton-dependent ATPases in both the F- and the V-branches of the phylogenetic tree.

**Conclusion:**

Barring convergent emergence of the same set of ligands in several lineages, these findings indicate that the use of sodium gradient for ATP synthesis is the ancestral modality of membrane bioenergetics. Thus, a primitive, sodium-impermeable but proton-permeable cell membrane that harboured a set of sodium-transporting enzymes appears to have been the evolutionary predecessor of the more structurally demanding proton-tight membranes. The use of proton as the coupling ion appears to be a later innovation that emerged on several independent occasions.

**Reviewers:**

This article was reviewed by J. Peter Gogarten, Martijn A. Huynen, and Igor B. Zhulin. For the full reviews, please go to the Reviewers' comments section.

## Background

The vast majority of the cellular ATP is produced by the membrane ATP synthases, reversible, rotary molecular machines that couple ion transfer across the membrane with the synthesis or hydrolysis of ATP. These machines belong to two distinct types, namely, F-type that is present in bacteria and eukaryotic organelles [1-4] and V-type, represented in archaea and in some bacteria [5-9]. The membranes of eukaryotic cells, in particular, *v*acuoles, contain V-type ATPases which use the energy of ATP hydrolysis to acidify cellular compartments [10-12].

The F/V-type ATPases couple transfer of protons or sodium cations across their membrane moieties with the concomitant hydrolysis or synthesis of ATP via a binding-change mechanism in their protruding catalytic parts [1,3,9,13]. Together with two evolutionarily unrelated families, the P-type ATPases and ABC transporters, the F/V ATPases belong to a heterogeneous group of enzymes that use the energy of ATP hydrolysis to translocate inorganic cations across membranes [14]. The F/V type ATPases, however, are unique functionally, because they can efficiently operate in the ATP synthase regimen, and mechanistically, in that their reaction cycle is accompanied by rotation of one enzyme part (rotor) relative to the other (stator) [3,15-17].

**Figure 1:**
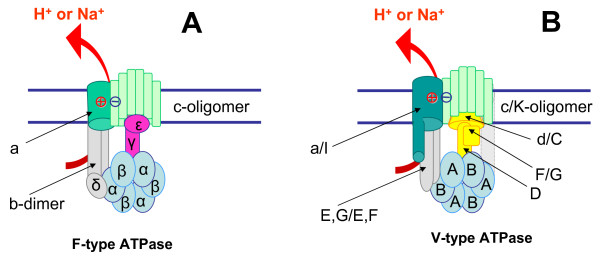
**Structure and evolutionary relationships of F- and V-type ATPases**. Orthologous subunits are shown by the same color and shape, and unrelated but functionally analogous subunits of the central stalk are shown by different colors and shapes. The subunits that show structural analogy but might not be homologous are shown by different but similar colors. The minimal, prokaryotic sets of subunits are depicted both for the F- and V-type ATPases. In the case of those V-ATPase subunits that are denoted by different letters in prokaryotes and eukaryotes, double notation is used: eukaryotic/prokaryotic. For further details, see ref. [37].

All F/V-ATPases have a mushroom-like structure, with the head protruding ~100 Å from the membrane (see refs. [5,7,9,12,18] and Figure 1). The head of the better studied F-type ATPase is a hexamer of three α and three β subunits (Figure 1A); each of the latter carries an ATP/ADP-binding catalytic site [18]. The ion-translocating, membrane-spanning F_O _sector of bacterial F-type ATPases is a complex of the integral membrane *a *subunit, two *b *subunits, and 10–15 small *c *subunits [3,19-21]; F_O _is connected to F_1 _by two distinct stalks [22,23]. The peripheral stalk consists of the protruding parts of the membrane-anchored *b *subunits that are connected to the α_3_β_3_ hexamer via the δ subunit [23]. The central stalk is formed by the elongated γ subunit that connects the two parts of the enzyme and the globular ε subunit that performs regulatory functions [18,22]. The F-type ATPase is a rotary dynamo machine in which sequential hydrolysis of ATP molecules by the α_3_β_3 _catalytic hexamer drives the rotation of the central stalk together with the ring of *c*-subunits [1,15-17]. The *c*-ring (rotor of the dynamo) is thought to slide along the interface with the *a *subunit that is rigidly bound via the peripheral stalk to the α_3_β_3_ hexamer and forms part of the stator; this sliding movement is coupled to the transmembrane ion transfer and generation of membrane potential. When the enzyme functions as ATP synthase, the ion current through F_O _causes the rotation of the γ_1_ε_1_*c*_10–15 _complex relative to the other subunits, and the catalysis of ATP synthesis is mediated by sequential interaction of the rotating γ subunit with the three catalytic β subunits [3,13,17,24].

The V-type ATPases, while sharing a common overall scaffold with F-ATPases, differ from them in many structural and functional features (see refs. [7,10-12] and Figure 1B). In particular, besides the large *a*-subunit and the *c*-oligomer (hereafter the eukaryotic notation is used [7]), the V_O _sector contains the *d *subunit, which serves as a socket for the D and F subunits of the central stalk of V_1 _[25,26].

The F/V-type ATPases are ubiquitous in the three domains of cellular life (bacteria, archaea, and eukaryotes) and comprise the centerpiece of cellular metabolism owing to their unique ability to utilize ion gradient for ATP production. Therefore, in addition to being the paradigmal biological nanomotors [17], these enzymes, depending on their ion specificity, define the nature of the bioenergetic cycle in any organism. The textbook bioenergetic cycle that operates in mitochondria, chloroplasts, and most prokaryotes includes the generation of proton-motive force (PMF) by primary transport systems (H^+^-pumps) and its utilization for ATP synthesis, as well as for solute transport, motility, reverse electron transport, etc [27]. However, certain extremophilic (in particular, thermophilic and alkaliphilic) and anaerobic bacteria and archaea use Na^+ ^as a coupling ion instead of or in addition to H^+ ^[28-30]. Similarly to the H^+ ^cycle, a Na^+ ^cycle includes primary Na^+ ^pumps, a Na^+^-transporting membrane ATP synthase, Na^+^-dependent membrane transporters, or a Na^+^-dependent flagellar motor.

Owing to its nearly ubiquitous presence in cells, the PMF has been generally viewed as the primary intermediate in biological energy transduction, in terms of both evolutionary history and of current importance for photosynthesis and respiration (see e.g. ref. [31]). By contrast, the ability of some prokaryotes to utilize sodium gradient for ATP synthesis is usually construed as a later exotic adaptation to survival in extreme environments [29,32-34]. Here we combine structural and phylogenetic analyses to show that sodium-translocating ATPases, most likely, antedated proton-translocating ATPases during evolution, so that the primordial membrane bioenergetics operated on sodium gradients.

## Results and discussion

### Phylogenetic distribution and evolutionary relationships of F- and V-ATPases

Sequence and structural comparisons of F- and V-type ATPases revealed homology between the subunits of the catalytic hexamer (α and B, β and A, respectively), membrane-embedded *c*/K-oligomers, and, perhaps, some subunits of the peripheral stalk (see Fig. 1), whereas the subunits of the central stalk have been found to be unrelated [8,35-37]. Based on this distribution of homologous and non-homologous subunits in the structure of F- and V-type ATPases, we recently outlined a scenario according to which F/V-ATPases evolved from ATP-dependent RNA/protein translocases that contained the translocated polymer at the place of the central stalk and, initially, functioned within primordial membranes that were permeable for small ions, but not for RNA or protein [37].

This analysis seems to bring some certainty into the hotly debated classification of rotary ATPases. While most authors prefer to speak of eukaryotic and bacterial V-type ATPases [7,9,38], some consider prokaryotic V-ATPases to be a separate group of A-type (from *a*rchaeal, as suggested by Gogarten and Starke [39]) ATPases, at the same level with eukaryotic V-type ATPases and F-type ATPases [5,40,41]. The latter division largely ignores the fact that V-type ATPase genes are encoded in many recently sequenced bacterial genomes, and the sequences of the bacterial V-ATPase subunits are closely related to their eukaryotic and archaeal counterparts (Table 1). Indeed, V-ATPases are the only type of membrane ATPases in all members of the bacterial phylum Deinococcus-Thermus [8] and most Chlamydia and Spirochaetes (Table 1). Taken together, the presence of an F-ATPase operon in *Protochlamydia amoebophila *(supposedly, an ancestral form of the phylum) and the existence of spirochetes that encode only F-ATPases (e.g. *Leptospira *spp.), as well as the universal presence of V-ATPases in archaea and their patchy phyletic distribution among bacteria, strongly suggest that bacteria acquired V-type ATPases from archaea, via multiple horizontal transfers [42]. Likewise, the presence of F-ATPase genes in the genomes of archaea *Methanosarcina acetivorans *and *Methanosarcina barkeri *[43], but not in *Methanosarcina mazei*, probably reflects acquisition of the corresponding genes from bacteria. In any case, Table 1 shows that V-ATPases are widespread in bacteria, many of which encode both F- and V-ATPases.

**Table 1:** Distribution of V-type ATPases in bacteria^a^

**Phylum, class**	**Species, strain**	**Complete genome**	**F-type ATPase**
**Bacteroidetes**			
	*Bacteroides caccae *ATCC 43185	-	**Y**
	*Bacteroides fragilis *YCH46	**Y**	**Y**
	*Bacteroides ovatus *ATCC 8483	-	**Y**
	*Bacteroides thetaiotaomicron *VPI-5482	**Y**	**Y**
	*Bacteroides uniformis *ATCC 8492	-	**Y**
	*Bacteroides vulgatus *ATCC 8482	-	**Y**
	*Parabacteroides distasonis *ATCC 8503	-	**Y**
	*Parabacteroides merdae *ATCC 43184	-	**Y**
	*Porphyromonas gingivalis *W83	**Y**	-
	*Psychroflexus torquis *ATCC 700755	-	**Y**
**Chlamydiae**			
	*Chlamydia muridarum *Nigg	**Y**	-
	*Chlamydia trachomatis *D/UW-3/CX	**Y**	-
	*Chlamydophila abortus *S26/3	**Y**	-
	*Chlamydophila caviae *GPIC	**Y**	-
	*Chlamydophila felis *Fe/C-56	**Y**	-
	*Chlamydophila pneumoniae *CWL029	**Y**	-
	*Protochlamydia amoebophila *UWE25	**Y**	**Y**
**Deinococcus-Thermus**			
	*Deinococcus geothermalis *DSM 11300	**Y**	-
	*Deinococcus radiodurans *R1	**Y**	-
	*Thermus thermophilus *HB8	**Y**	-
**Firmicutes**			
Bacilli	*Carnobacterium *sp. AT7	-	**Y**
	*Enterococcus faecalis *V583	**Y**	**Y**
	*Enterococcus faecium *DO	-	**Y**
	*Enterococcus hirae*	-	**Y**
	*Streptococcus gordonii *str. Challis	**Y**	**Y**
	*Streptococcus pneumoniae *TIGR4	**Y**	**Y**
	*Streptococcus pyogenes *M1 GAS	**Y**	**Y**
	*Streptococcus sanguinis *SK36	**Y**	**Y**
Clostridia	*Caloramator fervidus *ATCC 43024	-	n/d
	*Clostridium bolteae *ATCC BAA-613	-	**Y**
	*Clostridium botulinum *A str. ATCC 3502	**Y**	**Y**
	*Clostridium difficile *630	**Y**	**Y**
	*Clostridium novyi *NT	**Y**	**Y**
	*Clostridium perfringens *str. 13	**Y**	**Y**
	*Clostridium phytofermentans *ISDg	**Y**	**Y**
	*Clostridium *sp. L2–50	-	**Y**
	*Clostridium tetani *E88	**Y**	-
	*Clostridium thermocellum *ATCC 27405	**Y**	**Y**
	*Coprococcus eutactus *ATCC 27759	-	**Y**
	*Dorea longicatena *DSM 13814	-	**Y**
	*Eubacterium ventriosum *ATCC 27560	-	**Y**
	*Halothermothrix orenii *H 168	-	**Y**
	*Ruminococcus gnavus *ATCC 29149	-	**Y**
	*Ruminococcus torques *ATCC 27756	-	**Y**
	*Ruminococcus obeum *ATCC 2917	-	**Y**
	*Thermoanaerobacter ethanolicus *ATCC 33223	**Y**	-
Mollicutes	*Acholeplasma laidlawii *PG-8A	**Y**	**Y**
	*Eubacterium dolichum *DSM 3991	-	**Y**
**Fusobacteria**			
	*Fusobacterium nucleatum *subsp. *nucleatum *ATCC 25586	**Y**	**Y**
	*Fusobacterium nucleatum *subsp. *polymorphum *ATCC 10953	-	**Y**
	*Fusobacterium nucleatum *subsp. *vincentii *ATCC 49256	-	**Y**
**Proteobacteria**			
Alpha	*Labrenzia *(*Stappia) aggregata *IAM 12614	-	**Y**
Gamma	*Beggiatoa *sp. PS	-	**Y**
	*Neptuniibacter caesariensis*	-	**Y**
	*Nitrococcus mobilis *Nb-231	-	**Y**
	*Nitrosococcus oceani *ATCC 19707	**Y**	**Y**
Delta	*Anaeromyxobacter dehalogenans *2CP-C	**Y**	**Y**
	*Anaeromyxobacter *sp. Fw109-5	**Y**	**Y**
	*Geobacter uraniireducens *Rf4	**Y**	**Y**
**Spirochaetes**			
	*Borrelia afzelii *PKo	**Y**	-
	*Borrelia burgdorferi *B31	**Y**	-
	*Borrelia garinii *PBi	**Y**	-
	*Treponema denticola *ATCC 35405	**Y**	-
	*Treponema pallidum *subsp. *pallidum*	**Y**	-
**Thermotogae**			
	*Thermotoga *sp. RQ2	-	**Y**
	*Thermotoga neapolitana *DSM 4359	-	**Y**
	*Thermotoga petrophila *RKU-1	**Y**	**Y**
**Cyanobacteria**			
	*Synechococcus *sp. WH 5701	-	**Y**
**Planctomycetes**			
	*Kuenenia stuttgartiensis*	-	**Y**

^a ^The table lists bacterial species (with strain numbers) whose genomes have been found to encode V-type ATPases based on BLAST [109] searches of the NCBI non-redundant protein database. Availability of complete genome sequences is listed as of 12/31/2007. The absence of the F-ATPase genes in complete genomes is indicated with a dash; in incomplete genomes, it is marked as n/d, which stands for "not detected".

Furthermore, the absence of homology between the subunits of the central stalk indicates a major dichotomy between the F-type ATPases, on the one hand, and V-type ATPases, both prokaryotic and eukaryotic, on the other hand. In contrast, all subunits of prokaryotic V-type ATPases (A-type ATPases, according to refs. [5,40,41]) have their counterparts in the vacuolar V-ATPases of eukaryotes. Eukaryotic V-ATPases are more complex than their archaeal and bacterial counterparts owing to the presence of several additional subunits [7,9]. A similar phenomenon, however, is observed in F-ATPases, where the mitochondrial enzyme has several extra subunits as compared to the bacterial one [2,44]. Sequence analysis also indicates that the primary evolutionary split lies between the F-type and V-type ATPases followed by secondary splits between the prokaryotic and eukaryotic enzymes in each of the branches: in phylogenetic trees, bacterial, archaeal and eukaryotic V-ATPase subunits invariably cluster together and separately from the F-type ATPases [35,42,45]. This is a remarkable, rather rare case where the evolutionary affinity between archaea and eukaryotes is seen outside the information processing systems. Thus, consideration of A-type ATPases as a separate class of rotary ATPases does not seem fully justified, not to mention that it obscures the use of the archaeal enzyme as a model for studying the eukaryotic V-ATPase.

### Structural superposition of the Na^+^-binding sites of F- and V-ATPases

Proton-translocating and Na^+^-translocating forms are found among both F- and V-type ATPases. Notably, Na^+^-dependent ATPases are capable of translocating protons in the absence of Na^+ ^[29,34], whereas H^+^-dependent ATPases cannot translocate Na^+ ^[46]. The apparent cause of this asymmetry is that sodium translocation is more structurally demanding: the coordination number of Na^+ ^is 6, hence multiple, specific amino acid ligands are required to draw a sodium cation from water and to keep it in the non-polar inner space of the membrane.

**Figure 2:**
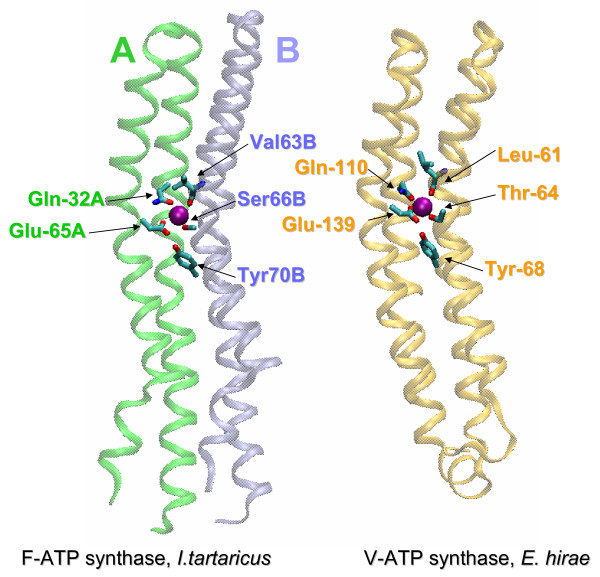
**Structures of the Na^+^-binding sites in the membrane rotor subunits of the Na^+^-translocating ATP synthase of F-type and V-type**. Left, *c *subunit of the Na^+^-translocating F-type ATP synthase of *Ilyobacter tartaricus *(PDB entry 1YCE [47]); right, NtpK subunit of the Na^+^-translocating V-type ATP synthase of *Enterococcus hirae *(PDB entry 2BL2 [38]). Note that in *I. tartaricus *the Na^+ ^ion (purple) crosslinks two identical subunits, A (green) and B (ice-blue), while in *E. hirae *the Na^+ ^ion is bound by a four-helical bundle that results from a subunit duplication. In both structures, major coordinating bonds to the Na^+ ^ion are provided by the principal ligand (Glu65A in *I. tartaricus *and Glu139 in *E. hirae*); other bonds come from a conserved glutamine (Gln32A in *I. tartaricus *and Gln110 in *E. hirae*), a hydroxy group of Ser66B in *I. tartaricus *and Thr64 in *E. hirae *(as initially predicted from sequence comparison [48] and a backbone carbonyl (Val63B in *I. tartaricus *and Leu61 in *E. hirae*). In *E. hirae *one more bond is provided by Gln65 (not shown, see the text). The remaining bonds are provided, most likely, by the unseen water molecules.

**Figure 3:**
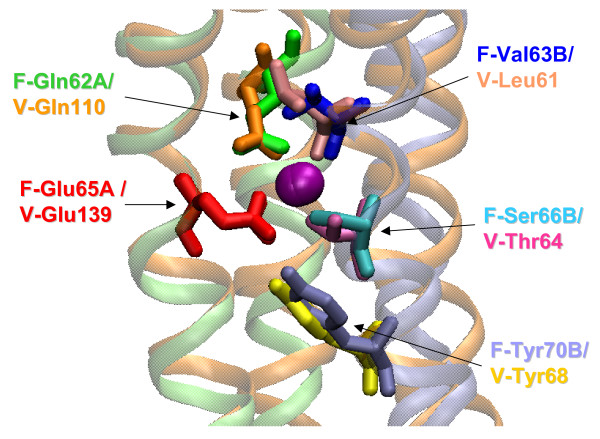
**Structural superposition of the Na^+^-binding sites of the F-type and V-type membrane ATPases**. The structures are the same as in Fig. 2. Note the overlap between the Na^+ ^ligands, as well as the non-ligating tyrosine (Tyr70B in *I. tartaricus *and Tyr68 in *E. hirae*) that is located beneath the Na^+ ^ion and stabilizes the principal Glu ligand.

**Figure 4:**
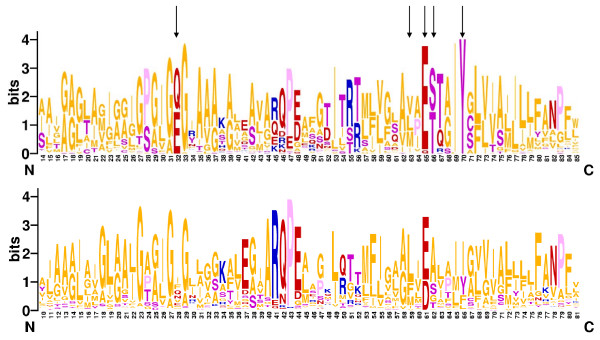
**Consensus sequences of the transmembrane segments of Na^+^-binding (top) and H^+^-binding (bottom) *c *subunits of prokaryotic F-type and V-type ATPases represented in the Sequence Logo format**. The height of each letter is proportional to the frequency of the respective amino acid in the given position [105, 106]. Residue numbering in the top and bottom panels follows the *c *subunits from *Ilyobacter tartaricus *[47] and *Escherichia coli*, respectively. The residues shown on Fig. 3 are indicated with arrows. The logo was constructed based on an alignment of single-hairpin c subunits (see Additional File 1). The alignment of Na^+^-translocating c subunits also included 13 hairpin domains from the *Methanopyrus kandleri *ATP synthase (see text and ref. 50). Note that the conservation of the Na^+^-ligands (e.g. Gln/Glu32, Glu65, Ser/Thr66) is partly absent in H^+^-binding subunits. It is noteworthy that the sodium ligands did not disappear completely; in addition to the principal acidic (Glu or Asp) residue, some Na^+ ^ligands are conserved in a variety of F-type and V-type H^+^-ATPases (see Additional File 1). This might reflect binding of the H_3_O^+ ^ion, rather than free proton, as first suggested by Boyer [107], in some enzymes, given that H_3_O^+ ^requires at least three coordination bonds [108].

The structures of the membrane-spanning, rotating oligomers of two Na^+^-translocating ATP synthases have been recently reported. The oligomer ring of the *Ilyobacter tartaricus *F-type ATP synthase consists of 11 *c*-subunits [47] whereas the ring of the *Enterococcus hirae *V-ATP synthase is composed of 10 K-subunits [38]. These structures show the same overall configuration of the Na^+^-binding sites (Fig. 2). All the four Na^+^-binding amino acid residues of *I. tartaricus *have readily identifiable counterparts in the *E. hirae *ATPase, and the two sets of ligands perfectly superimpose in space (Fig. 3). Even the position of a non-ligating tyrosine that stabilizes the principal, Na^+^-binding glutamate [34] is conserved. The K subunit of *E. hirae *ATPase contains an additional Na^+^-binding ligand, Gln65 [38], which is not shown on Fig. 3. The corresponding residue in the *I. tartaricus c*-subunit, Thr67, is much smaller, but, apparently can bind Na^+ ^ion through a water molecule. Sequence alignment of c/K subunits (Additional File 1) shows that all (predicted) Na^+^-translocating ATPases contain, in this position, a polar residue, Ser, Thr, Gln, or Arg, that is capable of coordinating the Na^+ ^ion either directly (Gln or Arg) or, in the case of Ser and Thr, via a water bridge [48]. In contrast, in all (predicted) H^+^-translocating ATPases, the corresponding position is occupied by an aliphatic residue incapable of making a hydrogen bond (Leu, Ile or Val). The sixth coordinating bond for the Na^+ ^ion, in all likelihood, is provided by a water molecule. In enzymes of both types, this water molecule might occupy the pocket between the conserved Pro/Ser28, Gly/Ala29 (see Fig. 4), and the Na^+ ^cation.

High-resolution crystal structures of H^+^-translocating *c*-oligomers are currently unavailable. However, sequence comparison indicates that they lack at least some residues that function as Na^+ ^ligands except for the principal acidic (Glu or Asp) cation-binding residue (see Fig. 4 and Additional File 1).

### The ancestral status of sodium bioenergetics inferred from phylogenetic analysis

The identical arrangement of the Na^+^-binding sites in the F-type and V-type Na^+^-translocating ATPases implies two mutually exclusive evolutionary scenarios:

1) the ancestor of the F/V ATPases was a H^+^-translocating enzyme, so the Na^+^-binding sites evolved independently in the two lineages via the recruitment of the same residues in the same positions, i.e., by convergence;

2) the common ancestor of the F/V ATPases was a Na^+^-translocating enzyme, but the ability to bind and translocate Na^+ ^was independently lost in multiple lineages of F- and V-ATPases, yielding H^+^-translocating enzymes.

**Figure 5:**
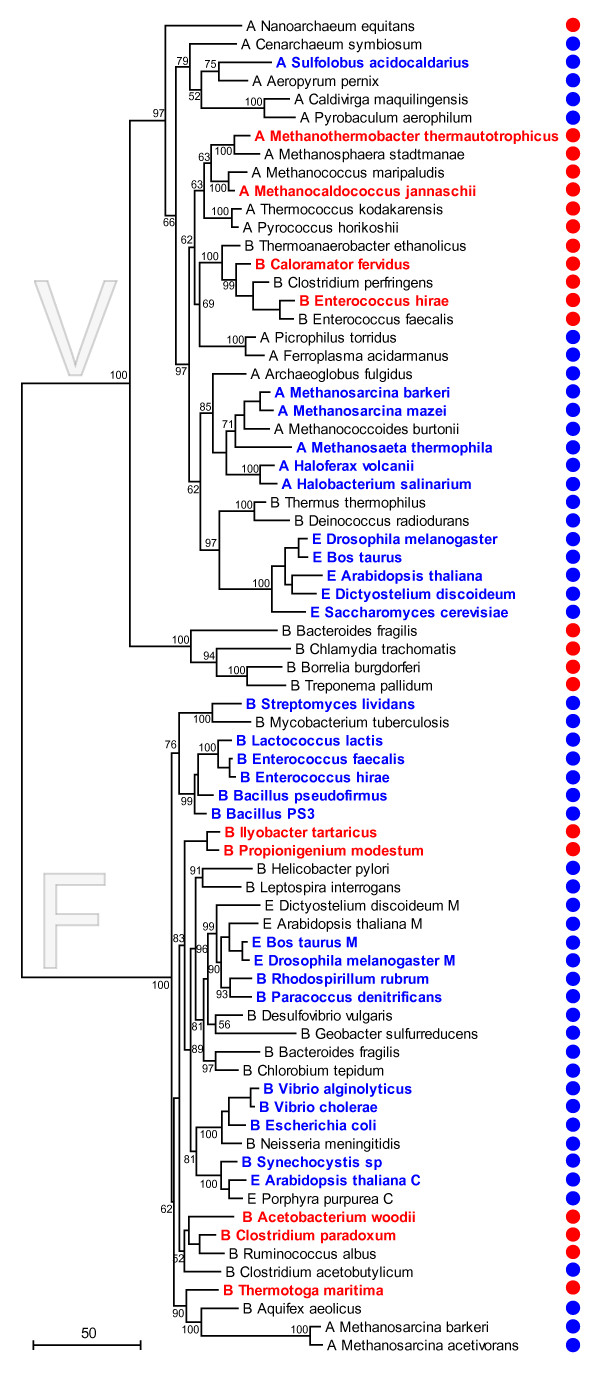
**Phylogeny of Na^+^-translocating and proton-translocating F/V-ATPases in Archaea (A), Bacteria (B) and Eukarya (E)**. The maximum-likelihood phylogenetic tree was constructed on the basis of a sequence alignment of β-subunits of F-ATPases and A subunits of V-ATPases (see Additional File 2). The names of organisms with experimentally characterized ATPases are shown in bold and colored red for Na^+^-dependent enzymes and blue for H^+^-dependent enzymes. The ATPases whose c subunits carry all Na^+^-ligands and are hence predicted to translocate sodium are denoted by red circles, and those lacking one or more of these ligands (and so predicted to translocate protons only) are denoted by blue circles. The numbers at internal branches indicate the RELL bootstrap probabilities (expressed as percentage points). The root position was forced between the F- and V-ATPases.

Convergence in the evolution of enzymes, manifest in the independent evolution of the same set of ligands conferring the same specificity in two lineages, is rare but not unheard of, as illustrated by the seminal study of Stewart et al. on the evolution of the langur monkey lysozyme that independently gained the same 7 ligands as the ungulate lysozymes during adaptation to a cellulolytic diet [49]. Thus, to distinguish between the two evolutionary scenarios, we constructed a phylogenetic tree of the catalytic subunits of the V- and F-ATPase and superimposed on it the known or predicted cation specificity, based on the available experimental data and presence or absence of the complete set of Na^+^-binding ligands (Fig. 5). The catalytic subunits were employed for this analysis because these are large proteins with numerous phylogenetically informative positions (see Additional File 2); by contrast, membrane-spanning *c*-subunits that harbor the cation-binding sites are short proteins that carry a weak phylogenetic signal, at best [45].

In the resulting tree, Na^+^-translocating ATPases did not form a clade within either the F or the V branch but instead comprised three distinct lineages among V-ATPases and at least three lineages among F-ATPases (Fig. 5). The monophyly of each clade of Na^+^-translocating ATPases was supported by moderate to high bootstrap values. In each of these clades, the same set of Na^+ ^ligands was conserved (Fig. 4). The independent emergence of (nearly) identical ensembles of Na^+ ^ligands in 6 distinct lineages of Na^+^-translocating ATPases can be, effectively, ruled out, leading to the conclusion that the common ancestor of V- and F-ATPases had a Na^+^-binding site. Unlike independent gain of the Na^+ ^ligands in multiple lineages, multiple independent losses of subsets of these ligands appear plausible and could have led to the multiple transitions to proton translocation, provided that the membranes in the respective organisms became proton-tight.

**Figure 6:**
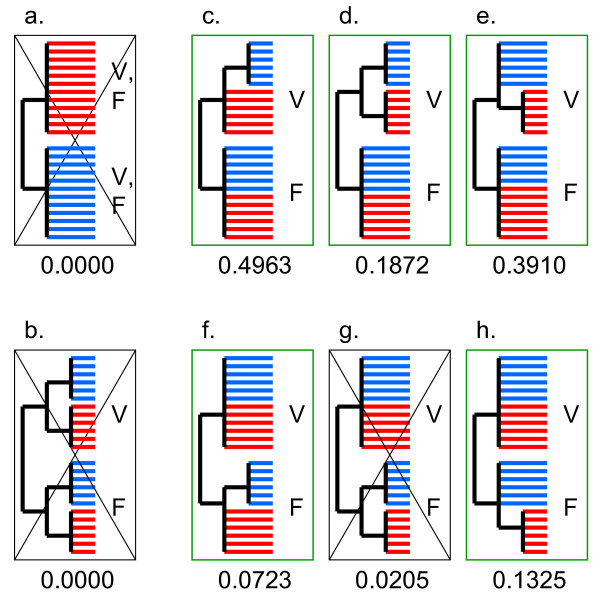
**Statistical analysis of F/V-ATPase tree topology**. The schematic shows the constraints on the tree topology and the results of the AU-test in comparison to the unconstrained tree. Red branches denote (predicted) Na^+^-dependent ATPases, and blue branches denote (predicted) H^+^-dependent ATPases. The corresponding AU-test p-value is shown for each constrained topology; trees with p-values < 0.05 are rejected (crossed frame). a. monophyly of all Na^+^-dependent and H^+^-dependent enzymes regardless of the type (F or V); b. monophyly of Na^+^-dependent and H^+^-dependent enzymes within V-ATPase and F-ATPase clades each; c. Na^+^-dependent V-ATPases paraphyletic to monophyletic H^+^-dependent V-ATPases; F-ATPases unconstrained; d. both Na^+^-dependent and H^+^-dependent V-ATPases monophyletic; F-ATPases unconstrained; e. H^+^-dependent V-ATPases paraphyletic to monophyletic Na^+^-dependent V-ATPases; F-ATPases unconstrained; f. Na^+^-dependent F-ATPases paraphyletic to monophyletic H^+^-dependent F-ATPases; V-ATPases unconstrained; g. both Na^+^-dependent and H^+^-dependent F-ATPases monophyletic; V-ATPases unconstrained; h. H^+^-dependent F-ATPases paraphyletic to monophyletic Na^+^-dependent F-ATPases; V-ATPases unconstrained.

The caveat beyond this conclusion is, of course, the uncertainty of the tree topology which remains a concern in any phylogenetic tree involving the deepest branchings in life's history. To address this concern, we performed statistical testing of alternative phylogenetic hypotheses using the approximately unbiased (AU) test. The AU test showed that the transition between Na^+ ^and H^+ ^translocation occurred at least twice, i.e., at least once in the V-lineage and at least once in the F-lineage: the topology with monophyletic Na^+^-translocating and H^+^-translocating ATPases was unequivocally rejected (Figure 6a). Moreover, the topology where the primary split in both the V-lineage and the F-lineage was between Na^+^-specific and H^+^-specific forms was rejected as well (Figure 6b). This result suggests that the switch, actually, occurred more than once in both V-type and F-type ATPases although topologies with a single switch in one of the principal branches could not be formally rejected (Figure 6c–h). The test failed to discriminate between the topologies where Na^+^-translocating or H^+^-translocating forms occupy the basal position in either the V-ATPase and F-ATPase clades (Fig. 6c,e,f,h). As discussed above, extraneous criteria, namely, the far greater likelihood of multiple losses of Na^+ ^ligands compared to that of multiple, independent acquisitions of these ligands strongly point to the ancestral status of Na^+ ^translocation.

### Evolution of the c subunits of F/V-type ATPases by duplication and loss of Na^+ ^ligands

The *c *subunits of F-type and some prokaryotic V-type ATPases are formed by two transmembrane (TM) segments that fold into a single helical hairpin and are less than 100 amino acid residues long. Some of these proteins carry the complete set of Na^+^-ligands, e.g. in the *c *subunits of the F-ATPase of *Thermotoga maritima *and the V-ATPase of *Nanoarchaeum equitans *(see Additional File 1), whereas others have a partial set of Na^+ ^ligands or even retain only the principal Glu/Asp cation-binding residue (e.g. F-ATPase from *Escherichia coli *or V-ATPase from *Pyrobaculum aerophilum*). Thus, H^+^-specific ATPases could have evolved from Na^+^-specific ATPases on multiple occasions and via somewhat different routes. The alignment of 2-helical *c *subunits shows patterns of loss/replacement of cation-binding residues that are generally consistent within bacterial and archaeal phyla, suggesting that these replacements occurred early in the evolution of the respective lineages (see Additional File 1). This finding corroborates the recent report of von Ballmoos and Dimroth on different modes of proton-binding by different F/V-type ATPases [34]. The pH-profiles of (i) the ATP hydrolysis and (ii) the modification of the principal carboxyl residue by dicyclohexylcarbodiimide (DCCD) were similar in the Na^+^-binding F-type ATPase (*I. tartaricus and Propionigenium modestum*) and H^+^-translocating V-type ATP synthase of *Halobacterium salinarium*. However, these profiles dramatically differed from the corresponding profiles of the F-type ATPases of *E-coli*, spinach chloroplasts, and bovine mitochondria. The revealed difference in the modes of proton-binding in the active site might reflect independent transition to H^+^-ATPase in different lineages.

In *c/*K subunits of other V-type ATPases, the 2-TM hairpins are duplicated, resulting in proteins with 4 TM segments (Fig. 2). *Methanocaldococcus jannaschii *and some other methanogens have a triplication of the 2TM hairpin, whereas *Methanopyrus kandleri *encodes a single protein of 1021 amino acid residues that encompasses 26 TM segments in 13 hairpins [50]. An early phylogenetic analysis of the two-hairpin *c *subunits has suggested that they evolved via several independent gene duplication and fusion events [45]. Assuming that the ancestral state was a Na^+^-translocating single-hairpin *c *subunit similar to the ones in *T. maritima *or *N. equitans*, it becomes possible to reconcile the history of duplications of the sequences encoding 2TM hairpins with the accompanying changes in cation specificity. Obviously, gene duplication *per se *could have occurred without any loss of the Na^+^-binding residues, as seen, e.g., in the proteins from *Methanothermobacter thermautotrophicus *or *Methanobrevibacter smithii *(see Additional File 1). In the *M. kandleri *protein, each of the 13 hairpins retains the complete set of Na^+^-binding residues such that one Na^+ ^ion per hairpin can be bound (see Additional File 1). However, in other V-ATPases, the duplication of the 2TM hairpins is often accompanied by the loss of one or more Na^+ ^ligands in one or both hairpins. In these cases, the hairpin duplication is not accompanied by the increase in the number of bound Na^+ ^ions (as in *E. hirae*, see Fig. 2). While in single-hairpin *c *subunits, as discussed above, the loss of even one ligand is likely to hamper Na^+^-binding, in the double-hairpin but single Na^+^-ion-binding *c*/K subunits, replacement/reshuffling of Na^+^-binding residues appears to be possible, being, however, constrained so that residues from two different hairpins provide the full complement of ligands for a Na^+ ^ion as seen in the *E. hirae *V-ATPase (Fig. 2).

### Primordial sodium bioenergetics and evolution of membranes

The possibility that sodium bioenergetics antedated proton bioenergetics has been considered previously [28,30,51] but has not drawn much attention, and proton-dependent bioenergetics is widely believed to be ancestral [31,34,52]. This view is attractive considering the intrinsic chemical coupling between protonation/deprotonation events and redox reactions, in particular, those of water and diverse quinones [53-55]. However, proton energetics demands sophisticated, proton-tight membranes. In the scenario of the origin of F/V-ATPases from ATP-dependent RNA/protein translocases [37], with the translocated polymer initially taking the position of the central stalk, these primordial tranlocases were proposed to function within primitive, ancient membranes that were permeable for both H^+ ^and Na^+ ^but not for RNA or protein. Under this model, the evolution of F/V-ATPases was concomitant with the evolution of modern, complex, ion-tight membranes from primordial, semi-permeable membranes [37]. Conceivably, the ancient translocase employed sodium cations to crosslink and stabilize the subunits of the *c*-oligomer (as in *I. tartaricus*, see Fig. 3), preventing its destruction by the translocated polymer. Thus, even at the stage of the RNA/protein translocase, when the primordial membranes might have been leaky to both Na^+ ^and H^+^, there could have been a mechanistic demand for Na^+^-binding and, accordingly, selection for the corresponding set of amino acid ligands. This scenario is supported by experiments demonstrating a dramatic decrease in the stability of the *c*-oligomers of Na^+^-translocating F-ATPases from *I. tartaricus *and *P. modestum *in the absence of Na^+ ^[56].

When considering the possibility that primordial bioenergetics functioned on a sodium gradient, a biogeochemical reality check is important: was there enough sodium in the ancient ocean? Currently, there is a consensus that the primordial ocean formed owing to the condensation of the atmospheric water vapor upon cooling of the Earth [57]. Hence, the primordial ocean was fresh at the time of its emergence. However, it became salty relatively fast as can be judged from the chemical composition of geologically trapped water (reviewed in [57,58]). In particular, quartz crystals found in iron oxide structures from the 3.5-3.2 Ga Barberton greenstone belt, South Africa, contain fluid inclusions with a Na/Cl ratio that is the same as in the present-day ocean (0.858), but the total amount of sodium and chlorine in these inclusions is 1.6 times the present day value [59]. Similarly, primary fluid inclusions from intra-pillow quartz from the North Pole Dresser Formation Pilbara craton, Western Australia (3.490 Ga), contain saline fluid with 12 wt% equivalent NaCl and a Cl/Br ratio of 631 [60]. Since this Cl/Br ratio is very close to that of modern seawater (Cl//Br = 647), it appears likely that this saline fluid is, indeed, archaeal seawater. Its higher salinity as compared to present seawater is believed to be due to the more intensive leaching of sodium (and other metals) from the crust by hot hydrothermal fluids [57,58]. Only starting from Proterozoic terrains, the water inclusions show salinity close to that of modern seawater [61], perhaps, reflecting certain calming of the hydrothermal activity.

**Figure 7:**
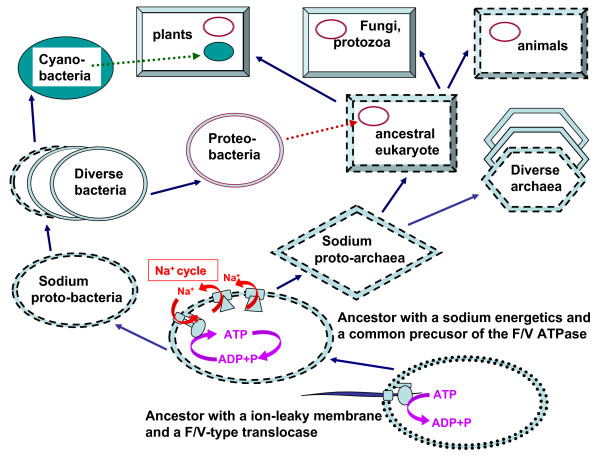
**The proposed scenario for the evolution of membrane bioenergetics**. The scheme shows the proposed transition from primitive membranes that were leaky both to Na^+ ^and H^+ ^(dotted lines), via membranes that were Na^+^-tight but H^+^-leaky (dashed lines) to the membranes that were impermeable to H^+ ^and Na^+ ^(solid lines). Dashed arrows show symbiotic acquisitions of α-proteobacteria (purple arrow) and of cyanobacteria (green arrow). The scheme emphasises that proton tightness of the membranes was achieved in different ways in different lineages (see text).

Thus, the above data indicate that, by the time of emergence of the first cells, sodium concentration in the ocean was comparable to or even exceeded that in extant oceans. Therefore, the next stage of evolution can be envisaged as selection for tighter membranes that would maintain the ionic homeostasis of the evolving cells, which would be particularly important given the increasing ocean salinity, and concomitantly, would create the opportunity for the utilization of ion gradients. As suggested by Skulachev [28], sodium-tight membranes could precede proton-tight membranes as structurally less demanding (see also below). In response to the increasing salinity of the primordial ocean, cells would need a mechanism for pumping Na^+ ^ions out of the cell, driving the transition from a protein translocase to the precursor of an ion-translocating membrane ATPase, as described elsewhere [37]. These ancestral ATPases would pump Na^+ ^along with the Na^+^-transporting pyrophosphatase [62] and chemically-driven Na^+^-pumps, such as Na^+^-transporting decarboxylase [29,63], which, being found in both bacteria and archaea, appear to antedate the divergence of the three domains of life. Unlike the other Na^+ ^pumps, the common ancestor of the V/F-ATPases, by virtue of its rotating scaffold, would be able to translocate Na^+ ^ions in both directions, so that, upon further increase in the external salinity, reversal of the rotation could result in Na^+^-driven synthesis of ATP by this primordial rotary machine. This would be the beginning of membrane bioenergetics: together with the ancient Na^+ ^pumps, the V/F-type ATP synthase would complete the first, sodium-dependent bioenergetic cycle in a cell membrane (Figure 7).

The subsequent transition to the H^+^-dependent bioenergetics required proton-tight membranes. The conductivity of lipid bilayers for protons is by 5–7 orders of magnitude higher than the conductivity for Na^+ ^and other small cations [64-66] and dramatically increases with temperature [32]. This difference stems from the unique mechanism of transmembrane proton translocation. Whereas transfer of other cations depends on their ability to penetrate the phospholipid membrane, crossing of the membrane/water interface by protons is not rate-limiting [64-67]: protons, being confined to the membrane ionizable groups [68], can apparently enter transient water clusters nested between the lipid hydrocarbon chains. The rate of proton transfer across the membrane is limited by proton "hopping" across the middle of the bilayer, with the protons transferred from one side of the membrane to the other via collisions between a protonated water cluster in one monolayer and a cluster in the opposite monolayer [65,66]. The rate of proton translocation, while independent of pH and insensitive to the H_2_O/D_2_O substitution [64-67], is determined by the frequency of these collisions which explains the dependence of the proton transfer rate on the overall bilayer dynamics and on temperature. Proton leakage can be suppressed by decreasing lipid mobility and/or increasing hydrocarbon density in the midplane of the bilayer, which can be achieved by either branching the ends of the lipid chains or incorporating hydrocarbons with a selective affinity to the cleavage plane of the bilayer [66].

In agreement with the hypothesis of independent evolution of proton energetics in multiple lineages, proton tightness of membranes appears to be achieved by radically different means in different organisms, namely, the mobility of side chains is restricted in distinct ways and diverse hydrocarbons are packed in the midplane of the H^+^-tight membranes [32,66]. Specifically, the fatty acid chains in the membrane of many bacteria have branched termini (see [66] and references therein). In some thermoacidophiles, the lipid chains terminate with alicyclics (cyclohexane and/or cycloheptane), resulting in additional crowding of the midplane of the bilayer [69]. In archaea, lipids consist of two phytanyl (isoprenoid) chains which are linked via a ether bond to glycerol or other alcohols such as nonitol [70]. Extreme thermophilic and acidophilic archaea possess membrane-spanning lipids in which the phytanyl chains of two diether lipids are fused to a C40 core [32,70]. Both the chain fusion and the involvement of a rigid ether bond restrict the hydrocarbon chain mobility. In addition, different organisms pack different hydrocarbons in the midplane of their H^+^-tight membranes, namely, phytosterols in plasma membranes of plants and protozoa, and hopanoids and squalane in bacteria (see [66] and references therein). In alkaliphilic bacteria, where H^+^-tightness is crucial, the squalane content is elevated as compared to mesophiles [71]. In eukaryotic mitochondria and chloroplasts, the middle of the bilayer is occupied by the ubiquinone and plastoquinone tails [72]. Remarkably, plasma membranes of animal cells, as emphasized in Fig. 4, have remained "sodium membranes" [28] inasmuch as they cannot maintain H^+ ^potential.

Taken together, the abundance of Na^+^-dependent thermophiles among deeply branching archaea and bacteria [30,32,73], the existence of unrelated mechanisms that make membranes H^+^-tight in different lineages [66], the discovery of opportunistic, chemically driven Na^+^-translocating enzymes in several deeply branching organisms [29,62,63,74], and the prevalence of the Na^+^-coupled membrane transport systems over H^+^-coupled ones in extremophiles [32] appear to provide strong support for the "Na^+^-translocating ATPase first" scenario. This scenario involves a gradual transition from the primordial membranes that were leaky to both Na^+ ^and H^+ ^to the currently predominant, more complex H^+^-tight membranes via Na^+^-tight, but H^+^-permeable membranes, which are still employed in animal cells, as well as, perhaps, in some bacteria and archaea with sodium energetics (Figure 7).

Considering the ubiquity of the F/V-ATPases in cellular life, their common ancestor is believed to have been present in the last universal common ancestor (LUCA) of cellular life forms [75]. Here we argue that this enzyme possessed a Na^+^-binding site. Thus, the LUCA either had leaky membranes such that the common ancestor of the F/V ATPases operated as polymer translocase with Na^+ ^ions performing a structural role, or the membrane of the LUCA was tight to sodium but permeable to protons and, accordingly, the LUCA would have sodium energetics (Figure 7). It seems highly unlikely that the LUCA had modern, proton-tight membranes: as discussed above, such membranes, probably, evolved later, independently in different lineages, a feature that might underline the fundamental difference in the lipid structure between bacteria and archaea [76].

As noted above, proton energetics has a major advantage over sodium energetics because proton transfer can be chemically coupled to redox reactions, thus, enabling the advent of efficient redox-driven generators of proton potential, such as cytochrome *bc*_1 _complex [55], cytochrome oxidase [54], or the oxygen-evolving photosystem II [77,78]. Once membranes capable of maintaining proton gradient have evolved, separately in bacteria and archaea, the sodium-binding sites of the F/V ATPases became dispensable and deteriorated independently in multiple lineages.

## Conclusion

The presence of essentially identical Na^+^-binding sites in the membrane-spanning *c/*K-oligomers of the F- and V-ATPases, combined with the scatter of Na^+^-dependent ATPases among the more common, H^+^-dependent ones in both the F and the V branches of the phylogenetic tree, leads to the unexpected conclusion that, during evolution, Na^+^-driven membrane bioenergetics preceded the proton-based energy conversion that is dominant in modern cells. This conclusion is further buttressed by the substantially greater conductivity of lipid bilayers to protons than to sodium cations and by the existence of distinct mechanisms that make membranes proton-tight in different lineages of cellular life. Under this scenario, the emergence of membrane bioenergetics was constrained by the evolution of the membranes themselves, not only the energy-converting enzymes.

## Methods

Multiple alignments of protein sequences were constructed using the MUSCLE program [79]. The consensus patterns of protein sequence alignments were derived using the SeqLogo format as implemented in the WebLogo tool {Crooks, 2004 #188;, 2007 #365}. Superposition of protein structures was performed using the VMD software [80]. Maximum-likelihood unrooted phylogenetic trees were constructed using the MOLPHY program (Jones-Taylor-Thornton model with uniform evolutionary rate across sites), and the same program was used to compute the RELL bootstrap probabilities from 10,000 replications [81,82]. Statistical analysis of tree topology was performed using the TreeFinder program [83], with the Whelan and Goldman (WAG) evolutionary model [84] and gamma-distributed site rates. Tree topologies were compared using the Approximately Unbiased (AU) test [85] implemented in the TreeFinder program.

## Competing interests

The author(s) declare that they have no competing interests.

## Authors' contributions

AYM contributed to the formulation of the original hypothesis and wrote the first draft of the manuscript; MYG contributed to the formulation of the original hypothesis and sequence analysis; KSM contributed to sequence analysis and performed phylogenetic analysis; YIW performed statistical analysis of phylogenetic trees; EVK designed the computational analyses and wrote the final version of the manuscript. All authors read, edited, and approved the final text.

## Reviewers' reports

### Reviewer's report 1

#### J. Peter Gogarten, Department of Molecular and Cell Biology, University of Connecticut, Storrs, Connecticut, USA

The manuscript by Mulkidjanian et al. puts forward the very interesting hypothesis that the V/F/A-ATPases first evolved as Na^+^-pumps and only later acquired specificity for protons several times independently. The main argument supporting this hypothesis is that the extant Na^+ ^translocating V/F/A-ATPases do not group together in phylogenetic reconstruction, and that it is unlikely that the more complex binding site in sodium translocating ATPases evolved multiple times independently. This hypothesis is also in agreement with the hypothesis, based on subunit stoichiometry, that these ATPases first evolved as ion pumps and that the switch to ATP synthases occurred more recently [86].

On page 10 the authors appear to overstate their case "The independent emergence of (nearly) identical ensembles of Na^+ ^ligands in at least 6 distinct lineages ... can be, effectively, ruled out". Indeed, 6 independent convergent events are *extremely *unlikely, but the number 6 seems to be derived from counting the groups of Na^+ ^pumping ATPases in Fig. 5. Cursory inspection shows that this tree is as unreliable as should be expected for a tree-of-life based on a single molecule. For example, *Nanoarchaeon *is grouped separately from all other archaeal homologs, whereas many multigene analyses [87,88] group Nanoarchaeon with the *Thermo*- and *Pyrococci*. If this were also true for the catalytic ATPases subunit, the number of independent convergent events is reduced by one. From work done in my lab [89] we know that the group constituted by ATPase catalytic subunits from *Chlamydia/Borrelia/Treponema/Bacteroides *is difficult to place, because these sequences are very divergent. These sequences can be attached almost anywhere within the A-ATPase part of the tree without significantly increasing the likelihood. I suspect that the situation for the F-ATPases is similar. In part this concern is addressed by the analysis depicted in Fig. 6, and this shows that a non Na^+ ^pumping origin of the V/F/A-ATPases necessitates the assumption of convergent evolution or gene transfer, but it is not "at least 6" independent events, but only at least 2 independent events that survive closer scrutiny.

##### Authors' response

*The questionable wording was modified. "At least 6 independent events" is indeed an (over)optimistic estimate whereas "at least 2 independent events" is a (overly) conservative one*.

I have one concern that the authors might be able to address experimentally: The phylogeny of the ATPases was calculated using the catalytic subunits of the ATPases. The authors' argument rests on the assumption that the ion translocating proteolipid subunit and the ATP hydrolyzing subunits evolved as a unit.

While this is a reasonable assumption, in ABC transporters the ATP binding cassette subunit not always co-evolves with the permease domains [90]. A similar situation might have occurred in the evolution of V/F/A-ATPases. While the proteolipid phylogeny is unresolved, using the AU test one could at least test if the proteolipid dataset is in significant conflict with the topology derived from the catalytic subunits.

##### Authors' response

*Given that the c/K subunits are short, highly diverged integral membrane proteins, we believe that any phylogenetic reconstruction and testing would produce, at best, unreliable results (not in the least due to the questionable applicability of the canonical evolutionary models to transmembrane segments). Thus, the evolutionary history of catalytic β/A subunit seems to be the best attainable approximation of evolution of the entire ATPase complex*.

##### Reviewer's reponse in a second review

I did not suggest to replace the analysis of catalytic subunits with that of the c/K subunits, but to test that the latter dataset is not in gross conflict with the phylogeny derived from the former.

##### Authors' response

*We appreciate this but even such tests hardly could be reliable given the unreliability of the c/*K *tree*.

##### Reviewer's report continued

I would advise more cautious interpretation in placing the evolution of ion specificity into the context of organismal evolution and in relation to the most recent common ancestor. As I pointed out previously, it can be misleading to only consider branches in the tree of life that have extant representatives [91]. Darwin's coral of life [92,93], in which the layer of extant organisms sits on the dead branches of their ancestors, might be a better picture to visualize evolutionary history [94]. One should consider the possibility that at the time of the organismal most recent common ancestor ATPases with different ion specificity already existed and that these were distributed into different extant lineages by horizontal gene transfer. These considerations do not impact the way the properties of the ancestral ATPase are reconstructed based on extant characteristics and phylogeny, but it changes the confidence with which we ascribe properties to the last universal common ancestor.

##### Authors' response

*Point well taken. We do not believe it necessary to modify the main text of the article but let this comment be the word of caution. We do not at all dispute the coral of life idea. Neither do we believe that the nature of LUCA is a resolved issue, so it is indeed a possibility, even if a non-parsimonious one, that, at this stage of evolution, both Na*^+^*-ATPases and H*^+^*-ATPases already existed. However, we would like to note that the devices for making a membrane proton-tight are much more complex than those that are required for sodium tightness, and that proton tightness seems to have evolved on multiple occasions during life's history (see main text). Therefore, it seems highly plausible that sodium bioenergetics antedated proton bioenergetics, even as we do not insist on our inference of the state of affairs in LUCA*.

The descriptions in the method section are very sparse. Was the tree depicted in Fig. 5 calculated using a model that takes among site rate variation into account? If not, this might explain the deep divergence of the Chlamydia/Borrelia/Treponema/Bacteroides sequences. These are fast evolving sequences, and their placement as the deepest branch within the V/A-ATPases likely is an artifact (see above).

##### Authors' response

*We did not find it necessary to expand the Methods section substantially given that the employed methods are well described in the literature. However, specifics on the evolutionary model used in our analysis were added. The tree depicted in *Fig. 5*was reconstructed with a model with a uniform evolutionary rate across sites. However, tree topology tests were performed using a model with gamma-distributed site rates. We found that taking into account rate variation across sites did not change the position of the V-type Chlamydia/Spirochetes/Bacteroidetes ATPase subunits. If this position is artifactual (which is a distinct possibility), the anomaly in the evolution of this branch is so substantial that its position is insensitive to the details of the evolutionary model used in phylogeny reconstruction*.

Last and least, the question of terminology addressed by the authors. I wholeheartedly agree that the primary split in the evolution of V/F/A-ATPases is between F-ATPases on one side and the V/A-ATPases on the other. I don't think that I ever said anything to the contrary. The classification into V-, F- and A-ATPases never was intended to result in co-equal categories, and in cladistic terms the A-ATPases likely represent a paraphyletic grouping. Nevertheless, I am not convinced that the distinction between V- and A-type ATPases should be abandoned.

##### Authors' response

*Currently, V-, F-, and A-ATPases are routinely treated as co-equal classes (e.g., references 5, 6, 40, and 41), which compelled us to make a clear distinction between the F-type and the V/A-type ATPases*.

While this split is less deep with respect to time (and less reflected in the primary sequence), with respect to function the A- and F-ATPases are more similar to each other than to the eukaryotic V-ATPases, which no-longer function as ATP synthases *in vivo*, but as dedicated proton pumps. Their function as an endomembrane and lytic compartment energizing ATPase the eukaryotes is distinct and different from that of the F- and A-ATPases. In my opinion this difference in physiological role justifies giving the eukaryotic vacuolar type ATPases a distinct name, and the term vacuolar-type seems appropriate, whereas labeling the archaeal counterpart as vacuolar type ATPases seems as strange as calling FtsZ a tubulin.

##### Authors' response

*In our opinion, the difference in the physiological roles of A-type and eukaryotic V-type ATPases, which is the ability of the former class, but not the latter one to function as ATP synthases is relatively minor, especially, given that bacterial V/A-type ATPases are capable of synthesizing ATP *[95,96]. *Whether the particular enzyme complex operates predominantly as an ATPase or ATP synthase, is determined by two factors. The first factor is the number of c-subunits per enzyme complex. The catalytic hexamer can always process 3 nucleotide molecules per full rotation. The number of ions that are translocated per rotation is directly determined by the number of c-subunits, which shows substantial variations. ATPases with 10 c-subunits, as in mitochondria, E. coli or E. hirae, can operate either as ATPases or as ATP synthases, depending on the conditions. If the number of c-subunits is large, e.g. 13–15, as in chloroplasts and cyanobacteria, then the driving force for ATP synthesis is stronger, so the enzyme preferentially operates as an ATP synthase. By contrast, if the number of c-subunits is small, e.g. six, as appears to be the case in eukaryotic V-ATPase, then the enzyme, on the one hand, can utilize the energy of ATP hydrolysis to generate a large pH-gradient of up to 4–5 pH units, but, on the other hand, would need an even larger ion-motive force to function as an ATP synthase. Apart from these thermodynamic considerations, these enzyme complexes are regulated. It has been shown that the conformation of the ε subunit in E. coli determines whether the enzyme complex operates as ATP synthase or as ATPase *[97,98]. *It might well be that such regulation also affects eukaryotic V-ATPases. Nevertheless, it cannot be ruled out that, when exposed to a sufficiently large ion gradient, even eukaryotic V-ATPases would be able to synthesize ATP. To the best of our knowledge, such experiments have never been reported*.

Also, in discussing this point the text seems a little unfair: as a criticism against the term A-ATPase, the authors argue that it "largely ignores the fact that V-type ATPase genes are encoded in many recently sequenced bacterial genomes", but on the following pages the authors themselves point out that these bacterial A-ATPases were likely acquired through HGT from archaeal donors. Clearly, A-ATPase is intended to stand for archaeal type ATPase, not ATPase found in Archaea.

##### Authors' response

*We could gladly agree with that distinction. However, ever since the first description in *Thermus thermophilus [99]*and *Enterococcus hirae [100], *bacterial non-F-type ATPases have been invariably referred to as V-type ATPases (whereas the reviewer has been using the term V/A-ATPase). The *E. hirae *ATPase has been referred to as V-ATPase in the recent structural paper by Murata et al*. [38]. *Thus, given the wide use of the term V-ATPase for bacterial enzymes, we felt compelled to note that there are no substantial differences between bacterial and archaeal enzymes of this class*.

##### Reviewer's response in a second review

The characteristic of eukaryotic V-ATPases is that their physiological role is to build up a proton gradient. This change is related to a change in proton/ATP stoichiometry which appears to be caused by the larger proteolipid size [101]; and appears to have occurred early in the evolution of eukaryotes [45]. The authors conjecture that given a sufficiently large driving force (electrochemical gradient for protons large as compared to the free energy of ATP hydrolysis) the eukaryotic V-ATPases can synthesize ATP; indeed, this was shown experimentally [102]. With respect to stoichiometry and quaternary structure the eukaryotic vacuolar type ATPases appear more uniform than the more diverse archaeal and bacterial V/A-ATPases. I don't think that those who use the term A-type ATPase imply that the A-, F- and V-ATPases are co-equal groups as suggested by the authors. The eukaryotic vacuolar type ATPases is clearly derived from its archaeal counterpart [103], and I don't think the authors of references *5, 6, 40, and 41 *suggest otherwise.

##### Authors' response

*We appreciate the pointer to the important work of Hirata et al.; their results indeed are quite compatible with the approach to classification of membrane ATPases taken here*.

### Reviewer's report 2

#### Martijn A. Huynen, Nijmegen Centre for Molecular Life Sciences, Radboud University, Nijmegen Medical Centre, Nijmegen, The Netherlands

The manuscript of Mulkidjanian et al presents a thoroughly documented and compelling argument for the primacy of an Na^+ ^dependent ATPase as the originator of both V-type and F-type ATPase. In general I find little argument with their thesis and I thoroughly enjoyed reading it.

Some points:

The lynch-pin of their argument is the very similar 3D structure of the residues that are involved in Na^+ ^binding in a V-type and in an F-type Na^+ ^ATPase. I do think that we actually also need 2 structures of H^+ ^transporting ATPases to make the case that the latter are less "conserved" and/or "constrained" in 3D structure. Although the results of Ballmoos and Dimroth, argue, of course, against a conserved mechanism of proton translocation.

##### Authors' response

*In order to assess structural constraints, at least, two structures seem necessary, indeed. However, this is not our argument here which is that, in H*^+^*-ATPases, the majority of Na*^+ ^*ligands are not conserved. Sequence analysis is sufficient to make this argument*.

As the point of the prevalence of sodium energetics among early branching is mentioned in the abstract, I would like to see this more explicitly in the manuscript. There are a lot of debates about which species are actually early branching, and this referee does not feel like reading that literature in order to figure out which species are referred to in this manuscript. Maybe it can also be mentioned more explicitly that although Na^+ ^transporters are widely distributed in life [28,62,63], it cannot be concluded form that literature that they are older than H^+ ^transporters.

##### Authors' response

*The issue with "early-branching" prokaryotes is, indeed, extremely difficult, and upon more careful deliberation, we decided to drop this argument. As for Na*^+ ^*transporters vs. H*^+ ^*transporters, it is not quite clear what H*^+ ^*transporters are meant*.

The "by virtue of its rotating scaffold" argument sounds logical, but is it also thermodynamically true? I thought that all reactions are, in principle, reversible.

##### Authors' response

*Of course, all biochemical reactions are, in principle, reversible. However, membrane ATPases are complex machines where one, in principle, reversible enzyme, the ATPase, is mechanistically coupled with another reversible enzyme, the membrane ion translocase. Whether the coupling between the chemical reaction and ion translocation is reversible or not, is determined by the coupling mechanism *[104]. *In particular, ATP synthesis by diverse ABC-transporters or P-type ATPases (in their theoretically possible reverse mode of operation) has never been reported. Apparently, the mechanics of these complexes are not compatible with ATP synthesis powered by the transmembrane ion gradient. Therefore, the rotating scaffold of the common ancestor of F/V-type ATPases was an innovation of substantial selective value, thanks to its ability to catalyze the ATP/ADP conversion in both directions by simply changing the direction of rotation. As already noted, at least some of the extant F-ATP synthases are not straightforwardly reversible owing to a fairly sophisticated regulatory mechanism *[97]. *However, this regulation is likely to have evolved after the emergence of the first rotary ion-translocating ATPase*.

### Reviewer's report 3

#### Igor B. Zhulin, Joint Institute for Computational Sciences, The University of Tennessee – Oak Ridge National Laboratory, Oak Ridge, Tennessee, USA

This work by Mulkidjanian *et al*. builds on their previous considerations of the evolution of the F-type and V-type ATPases [37]. They now provide a significant body of evidence pointing toward a sodium ion as the ancestral form of ion gradient-driven ATP synthesis. Historically, sodium bioenergetics was viewed as secondary to classical, more ubiquitous proton bioenergetics; therefore, the current study is paradigm-shifting. The paper is well-written and I do not have any specific concerns. Authors present independent lines of evidence to support their evolutionary scenario (Fig. 7). These involve several types of structural and sequence analyses, each carefully done and well presented. Phyletic distribution of sodium versus proton energetics provides an extra argument and logical reasoning rounds up the story. As a result, a usual dilemma – whether one favors gene loss over horizontal gene transfer (in this case, convergent evolution) – does not appear unsolvable. The probability of independent inventions is so much lower than gene loss that in this case it is almost prohibitive. Unless one provides strong counterarguments (I obviously do not have any), let sodium bioenergetics rule!

##### Authors' response

*We appreciate this positive review. We must point out, however, that, under the current scenario, evolution of proton bioenergetics involved independent loss of specific sets of amino acid residues (Na*^+ ^*ligands) rather than gene loss*.

## Supplementary Material

Additional file 1 Multiple alignment of the transmembrane segments of the c/K subunits of F- and V-type ATPases.p> Click here for file

Additional file 2 Multiple alignment of the catalytic subunits of F- and V-type ATPases used for the construction of the tree in Fig. 5 Click here for file
