# Supplementary material for: Evolutionary primacy of sodium bioenergetics
Source: Biol Direct. 2008 Apr 1;3:13. doi: 10.1186/1745-6150-3-13 (PMC2359735; doi:10.1186/1745-6150-3-13)
Supplement: Additional file 1 — Multiple alignment of the transmembrane segments of the c/K subunits of F- and V-type ATPases.p> [file 1745-6150-3-13-S1.doc]

**Additional File 1. Sequence alignment of 2TM-fragments of *c* subunits of F-type ATPases and c/K subunits of V-type ATPases**

**1. F- type ATPase c subunits**

| **Phylum, class** | **Organism name^a^** | **gi number^b^** | **Residues** | **2TM sequence (aligned)^c^** | **Ion^d^** | **Fig.4^e^** | **Fig.5^f^** |
| --- | --- | --- | --- | --- | --- | --- | --- |
| **PDB: 1YCE** | ***Ilyobacter tartaricus*** | [66360700](http://www.ncbi.nlm.nih.gov/entrez/viewer.fcgi?db=protein&id=66360700) | 14-85 | SAVGAGTAMIAGIG**P**GVG**Q**GYAAGKAVESVARQPEAKGDIISTMVLGQAVA**EST**GI**Y**SLVIALILLYANPFV | **Na^+^** | Y | Y |
| **Archaea** |  |  |  |  |  |  |  |
| **Euryarchaeota** | *Methanosarcina acetivorans* | [20091267](http://www.ncbi.nlm.nih.gov/entrez/query.fcgi?cmd=Retrieve&db=Protein&list_uids=20091267&dopt=GenPept) | 15-86 | IATSGLTIGIGVLG**P**AIG**E**GRAVATALSSLAQQPDASATITRTLFVGLAMI**ES**LSI**Y**CFVVSMILIFANPFW | H^+^ | Y | Y |
|  | *Methanosarcina barkeri* | [73670553](http://www.ncbi.nlm.nih.gov/entrez/query.fcgi?cmd=Retrieve&db=Protein&list_uids=73670553&dopt=GenPept) | 15-86 | IATAGITIGIGVIG**P**AIG**E**GRAVATALSSLAQQPDASATITRTLFVGLAMI**ES**LAI**Y**CFVVSMILIFANPFW | H^+^ | Y | Y |
| **Bacteria** |  |  |  |  |  |  |  |
| **Acidobacteria** | *Acidobacteria bacterium* 345 | [94968328](http://www.ncbi.nlm.nih.gov/entrez/viewer.fcgi?val=94968328) | 38-104 | PIAAGIGFGIAVGL**A**ALG**Q**GRVAASACESMARNPAGRAGIQLFLIFGLAFI**ES**LVLFAFVIVFIKVV----- | H^+^ | Y | ─ |
|  | *Solibacter usitatus* | [116619895](http://www.ncbi.nlm.nih.gov/entrez/viewer.fcgi?val=116619895) | 35-101 | PISAGFSMAIASGM**C**GLA**Q**AKAVAAAAEGMARNPGAAAAIRFALLLGLVLI**ES**LAL**Y**TLVIIFVKVT----- | H^+^ | Y | ─ |
| **Actinobacteria** | *Mycobacterium tuberculosis* | [15608445](http://www.ncbi.nlm.nih.gov/entrez/viewer.fcgi?db=protein&id=15608445) | 10-81 | LIGGGLIMAGGAIG**A**GIGDGVAGNALISGVARQPEAQGRLFTPFFITVGLV**E**AAYFINLAFMALFVFATPVK | H^+^ | Y | Y |
|  | *Corynebacterium diphtheriae* | [38233641](http://www.ncbi.nlm.nih.gov/entrez/query.fcgi?cmd=Retrieve&db=Protein&list_uids=38233641&dopt=GenPept) | 15-79 | GSIATVGYGIATIG**P**GLGIGILVGKALEGMARQPEMAGQLRTTMFLGIAFV**E**ALALIGLVAGFIL------- | H^+^ | Y | ─ |
|  | *Streptomyces lividans* | [405796](http://www.ncbi.nlm.nih.gov/entrez/viewer.fcgi?val=405796) | 10-76 | GSLGSIGYGLAAIG**P**GVGVGIIFGNGTQAMARQPEAAGLIRANQILGFAFC**E**ALALIGLVMPFVYGY----- | **H^+^** | Y | Y |
| **Aquificae** | *Aquifex aeolicus* | [3913149](http://www.ncbi.nlm.nih.gov/entrez/viewer.fcgi?val=3913149) | 32-100 | YLGAGLAIGLAGLG**A**GVGMGHAVRGTQEGVARNPNAGGRLQTLMFIGLAFI**ET**IAL**Y**GLLIAFILLFVV--- | H^+^ | Y | Y |
| **Bacteroidetes** | *Bacteroides fragilis* | [53713464](http://www.ncbi.nlm.nih.gov/entrez/viewer.fcgi?val=53713464) | 18-85 | KLGAALGAGLAVIG**A**GIGIGKIGGSAMEGIARQPEASGDIRMNMIIAAALV**E**GVALLALVVCLLVLFL---- | H^+^ | Y | Y |
|  | *Cytophaga hutchinsonii* | [110279286](http://www.ncbi.nlm.nih.gov/entrez/viewer.fcgi?val=110279286) | **12-79** | LAGAGIGAGVAALA**A**GLGIGRIGSSAVESIARQPSESGKIQTAMLIAAALI**E**GVALFGVVVCLLIALA---- | H^+^ | Y | ─ |
|  | *Salinibacter ruber* | [83758190](http://www.ncbi.nlm.nih.gov/entrez/viewer.fcgi?val=83758190) | 8-76 | YLAAGLGAGISAVG**A**AIGIGRLASSSMDGAARQPEAAGDIRGLMIVSAGLI**E**GVALFALIICLLLVLFV--- | H^+^ | Y | ─ |
| **Chlamydiae** | *Protochlamydia amoebophila* | [46447307](http://www.ncbi.nlm.nih.gov/entrez/query.fcgi?cmd=Retrieve&db=Protein&list_uids=46447307&dopt=GenPept) | 29-98 | ALSAPFAVGLAALG**S**GLGLGRAVSSAMEAIGRQPEASGKILTTMIIGAALI**E**ALTI**Y**ALIVFFVVLEKMA-- | H^+^ | Y | ─ |
| **Chlorobi** | *Chlorobium tepidum* | [21672861](http://www.ncbi.nlm.nih.gov/entrez/viewer.fcgi?val=21672861) | 7-73 | YLGAGIGAGLAAIG**A**GLGIGNAAASAAEGTARQPEAASDIRTTMIIAAALI**E**GVALFGEVICVLLALK---- | H^+^ | Y | Y |
|  | *Pelodictyon luteolum* | [78187939](http://www.ncbi.nlm.nih.gov/entrez/viewer.fcgi?val=78187939) | 8-75 | YLGAGLGAGLAVIG**A**GLGIGNIAASAAEGTARQPEATSDIRTTMIIAAALI**E**GVALFGEVICVLLALK---- | H^+^ | Y | ─ |
|  | *Prosthecochloris vibrioformis* | [145220543](http://www.ncbi.nlm.nih.gov/entrez/viewer.fcgi?val=145220543) | 8-75 | YLGAGIGAGLAVIG**A**GLGIGNIAASAAEGTARQPEATSDIRTTMIIAAALI**E**GVALFGEVICVLLALK---- | H^+^ | Y | ─ |
| **Chloroflexi** | *Chloroflexus aurantiacus* | [163848590](http://www.ncbi.nlm.nih.gov/entrez/viewer.fcgi?val=163848590) | 6-76 | LVATALAVGLGAIG**P**GVGIGIIVSGAVQAIGRNPEIENRVVTYMFIGIAFT**E**ALAIFGLVIAFLIGFGVLQ- | H^+^ | Y | ─ |
|  | *Dehalococcoides* sp. CBDB1 | [73660114](http://www.ncbi.nlm.nih.gov/entrez/viewer.fcgi?val=73660114) | 8-76 | LLAAGLAMGLGAIG**P**GIGVGILGFGALQAIGRNPEAKGSIFTNMILLVAFA**ES**IAIFALVISIVLIFVA--- | H^+^ | Y | ─ |
|  | *Herpetosiphon aurantiacus* | [159900580](http://www.ncbi.nlm.nih.gov/entrez/viewer.fcgi?val=159900580) | 8-77 | LLAAALAIGLAAIG**P**GIGVGLLVAGALQAIARNPETEGSIRTNMFVGIALT**E**GLAIFGLVISLLIGFGVL- | H^+^ | Y | ─ |
| **Cyanobacteria** | *Gloeobacter violaceus* | [37522478](http://www.ncbi.nlm.nih.gov/entrez/query.fcgi?cmd=Retrieve&db=Protein&list_uids=37522478&dopt=GenPept) | 10-81 | VIAAALAVGLAAIG**P**GIG**Q**GNAASKAAEGIARQPEAEGKIRGTLLLSLAFM**ES**LTI**Y**GLLVSIVLLFANPFR | H^+^ | Y | ─ |
|  | *Prochlorococcus marinus* | [33241057](http://www.ncbi.nlm.nih.gov/entrez/query.fcgi?cmd=Retrieve&db=Protein&list_uids=33241057&dopt=GenPept) | 10-81 | VVAAGLAVGLGAIG**P**GIG**Q**GSAAQGAVEGIARQPEAEGKIRGTLLLSFAFM**ES**LTI**Y**GLVVALVLLFANPFA | H^+^ | Y | ─ |
|  | *Synechocystis* sp. PCC6803 | [114677](http://www.ncbi.nlm.nih.gov/entrez/viewer.fcgi?db=protein&id=114677) | 10-81 | VIAAALAVGLGAIG**P**GIG**Q**GNASGQAVSGIARQPEAEGKIRGTLLLTLAFM**ES**LTI**Y**GLVIALVLLFANPFA | **H^+^** | Y | ─ |
| **Firmicutes** |  |  |  |  |  |  |  |
| ***Bacilli*** | *Bacillus subtilis* | [16080739](http://www.ncbi.nlm.nih.gov/entrez/viewer.fcgi?db=protein&id=16080739) | 3-70 | LIAAAIAIGLGALG**A**GIGNGLIVSRTVEGIARQPEAGKELRTLMFMGIALV**E**ALPIIAVVIAFLAFFG---- | H^+^ | Y | ─ |
|  | *Bacillus pseudofirmus* | [114665](http://www.ncbi.nlm.nih.gov/entrez/viewer.fcgi?db=protein&id=114665) | 3-69 | FLGAAIAAGLAAVA**G**AIAVAIIVKATIEGTTRQPELRGTLQTLMFIGVPLA**E**AVPIIAIVISLLILF----- | **H^+^** | N | Y |
|  | *Bacillus PS3* | [114678](http://www.ncbi.nlm.nih.gov/entrez/viewer.fcgi?db=protein&id=114678) | 5-72 | VLAAAIAVGLGALG**A**GIGNGLIVSRTIEGIARQPELRPVLQTTMFIGVALV**E**ALPIIGVVFSFIYLGR---- | **H^+^** | N | Y |
|  | *Enterococcus faecalis* | [29377098](http://www.ncbi.nlm.nih.gov/entrez/viewer.fcgi?db=protein&id=29377098) | 6-73 | FIAAAIAIFGSAIG**A**AIGNGQVISKTIESMTRQPEMSGQLRTTMFIGVALI**E**AVPILGVVVSLLLLFR---- | **H^+^** | N | Y |
|  | *Enterococcus hirae* | [114669](http://www.ncbi.nlm.nih.gov/entrez/query.fcgi?cmd=Retrieve&db=Protein&list_uids=114669&dopt=GenPept) | 3-71 | YIAAAIAIMGAAIG**A**GYGNGQVISKTIESMARQPEMSGQLRTTMFIGVALV**E**AVPILGVVIALILVFAV--- | **H^+^** | Y | Y |
|  | *Lactobacillus plantarum* | [28378943](http://www.ncbi.nlm.nih.gov/entrez/query.fcgi?cmd=Retrieve&db=Protein&list_uids=28378943&dopt=GenPept) | 3-70 | AIAAGIAMFGAALG**A**GIGNGLVISKMLEGMARQPELSGQLRTNMFIGVGLI**ES**MPIISFVVALMVMNK---- | H^+^ | Y | ─ |
|  | *Lactococcus lactis* | [15674249](http://www.ncbi.nlm.nih.gov/entrez/viewer.fcgi?db=protein&id=15674249) | 6-71 | AALGAIAIGLAALG**A**AIGDGLIVSNFLQAVARQPELEGKLRGSMFMGIAFV**E**G**T**FFIALAMAFLFR------ | **H^+^** | N | Y |
| ***Clostridia*** | *Acetobacterium woodii* | [4713918](http://www.ncbi.nlm.nih.gov/entrez/viewer.fcgi?db=protein&id=4713918) | 11-82 | SAIGAGIAMIAGVG**P**GIG**Q**GFAAGKGAEAVGRQPEAQSDIIRTMLLGAAVA**ETT**GI**Y**GLIVALILLFANPFF | **Na^+^** | Y | Y |
|  | *Acetobacterium woodii* | [6014714](http://www.ncbi.nlm.nih.gov/entrez/viewer.fcgi?db=protein&id=6014714)_1 | 23-99 | SALGIGLAMVAGVG**P**GIG**Q**GFAAGKGAEAVGKNPTKSNDIVMIMLLGAAVA**ETS**GIFSLVIALILLFANPFI | **Na^+^** | N | Y |
|  | *Acetobacterium woodii* | [6014714](http://www.ncbi.nlm.nih.gov/entrez/viewer.fcgi?db=protein&id=6014714)_2 | 110-182 | SAMASGIAMIAGIG**P**GTG**Q**GYAAGKGAEAVGIRPEMKSAILRVMLLGQAVA**QTT**GI**Y**ALIVALILMYANPFL |  | N |  |
|  | *Alkaliphilus metalliredigens* | [150388188](http://www.ncbi.nlm.nih.gov/entrez/query.fcgi?cmd=Retrieve&db=Protein&list_uids=150388188&dopt=GenPept) | 14-85 | SAIGAGLAMIAGIG**P**GIG**Q**GYAAGKGAEGVGRQPEAQGDIVRTMLLGAAVA**ETT**GI**Y**GLIIALILLFANPLV | Na^+^ | N | ─ |
|  | *Alkaliphilus metalliredigens* | [150388187](http://www.ncbi.nlm.nih.gov/entrez/viewer.fcgi?db=protein&id=150388187)_1 | 27-98 | TAIAAGLAMIAGIG**P**GIG**Q**GFAAGKGAEAASLNPKSAKSASMVMLLGAAVA**ETS**GILSLVVALIMLYANPLV | Na^+^ | N | ─ |
|  | *Alkaliphilus metalliredigens* | [150388187](http://www.ncbi.nlm.nih.gov/entrez/viewer.fcgi?db=protein&id=150388187)_2 | 110-181 | SVIGAGLAMIAGIG**P**GIG**Q**GYAAGKGTEMVGKRPQYQPMIVRTMFLGQAVA**QTT**GI**Y**ALIIALVLMFANPLV |  | N |  |
|  | *Carboxydothermus hydrogenof.* | [78043573](http://www.ncbi.nlm.nih.gov/entrez/query.fcgi?cmd=Retrieve&db=Protein&list_uids=78043573&dopt=GenPept) | 13-82 | AIGAGIAVGFGAIG**S**GIG**Q**GIAAGKAFEAMARQPEVRGTVQTFLIIALAFM**ET**LTI**Y**GLVIAFMLLNKMS-- | H^+^ | Y | ─ |
|  | *Clostridium acetobutylicum* | [5915735](http://www.ncbi.nlm.nih.gov/entrez/viewer.fcgi?db=protein&id=5915735) | 13-81 | QYLGAGLAAIGCIG**G**GVGIGTVTGKAVEAIGRQPESASKVMPTMIMGLAFA**E**V**T**SL**Y**ALFVAIMLLFVK--- | H^+^ | N | Y |
|  | *Clostridium difficile* | [115252533](http://www.ncbi.nlm.nih.gov/entrez/viewer.fcgi?db=protein&id=115252533) | 10-81 | SAIGAGIAVATGIG**A**GIG**Q**GIAAAKAAEAVGNQPEAKGDITSTLLLGVAIA**ESS**AI**Y**GLVISIILLFVNPFF | Na^+^ | Y | ─ |
|  | *Clostridium paradoxum* | [77964176](http://www.ncbi.nlm.nih.gov/entrez/viewer.fcgi?db=protein&id=77964176) | 10-81 | SAIGAGLAMIAGIG**P**GIG**Q**GFAAGKGAEAVGKQPEAQGDILRTMLLGAAVA**EST**GI**Y**ALVVALILLFANPLL | **Na^+^** | Y | Y |
|  | *Ruminococcus albus* | [2662061](http://www.ncbi.nlm.nih.gov/entrez/viewer.fcgi?db=protein&id=2662061) | 12-83 | SALGAGLAMIAGIG**P**GIG**E**GYAVGKTIESIARQPEAQGDCTRTMFIGVAMA**EST**GI**Y**AFVVALILMFGNPFI | Na^+^ | Y | Y |
|  | *Symbiobacterium thermophilum* | [51891224](http://www.ncbi.nlm.nih.gov/entrez/query.fcgi?cmd=Retrieve&db=Protein&list_uids=51891224&dopt=GenPept) | 8-77 | ALAAALSISVAAIG**A**TVA**Q**GKATTAAMDAIWRQPEAANDVRGALIVSLALM**E**AIAI**Y**GLLIGLLIIFMLG-- | H^+^ | Y | ─ |
| ***Mollicutes*** | *Mycoplasma genitalium* | [12045266](http://www.ncbi.nlm.nih.gov/entrez/viewer.fcgi?db=protein&id=12045266) | 32-102 | AYIGAGVTMIAGSTVGIG**Q**GYIFGKAVEAIARNPEVEKQVFKLIFIGSAVS**EST**AI**Y**GLLISFILIFVAGA- | Na^+^ | N | ─ |
|  | *Spiroplasma citri* | [110004061](http://www.ncbi.nlm.nih.gov/entrez/query.fcgi?cmd=Retrieve&db=Protein&list_uids=110004061&dopt=GenPept) | 31-109 | SLLGAGLAAIGCCG**S**GIG**Q**GYTGGKAVEAIARNPEVESKVRTQYIIAAAIT**ES**GSI**Y**ALVIAIILAFVTG-- | H^+^ | Y | ─ |
|  | *Ureaplasma parvum* | [13357693](http://www.ncbi.nlm.nih.gov/entrez/viewer.fcgi?db=protein&id=13357693) | 41-109 | KYIGTGITMLAAGAVGLM**Q**GFSTANAVQAVARNPEAQPKILSTMIVGLALA**E**AVAI**Y**ALIVSILIIFVA--- | H^+^ | Y | ─ |
| **Fusobacteria** | *Fusobacterium nucleatum* | [19703705](http://www.ncbi.nlm.nih.gov/entrez/query.fcgi?cmd=Retrieve&db=Protein&list_uids=19703705&dopt=GenPept) | 14-85 | SAVGAGLAMIAGLG**P**GIG**E**GYAAGKAVESVARQPEARGSIISTMILGQAVA**EST**GI**Y**SLVIALILLYANPFL | Na^+^ | Y | ─ |
|  | *Propionigenium modestum* | [114672](http://www.ncbi.nlm.nih.gov/entrez/viewer.fcgi?db=protein&id=114672) | 14-85 | SAVGAGLAMIAGLG**P**GIG**E**GYAAGKAVESVARQPEARGSIISTMILGQAVA**EST**GI**Y**SLVIALILLYANPFL | **Na^+^** | Y | Y |
| **Planctomycetes** | *Rhodopirellula baltica* | [32473397](http://www.ncbi.nlm.nih.gov/entrez/viewer.fcgi?db=protein&id=32473397) | 33-104 | IIMAGLTTAIGSIG**P**AFA**E**GRAVAQALNSIAQQPDSSNTITRTLFVGLAMI**EST**AI**Y**CFVVSMILLFANPFW | Na^+^ | Y | ─ |
|  | *Kuenenia stuttgartiensis* | [91201494](http://www.ncbi.nlm.nih.gov/entrez/query.fcgi?cmd=Retrieve&db=Protein&list_uids=91201494&dopt=GenPept) | 7-78 | LAIAVSLLAIAAFG**C**GIG**Q**GIAVYGAANGMARQPDMAGKIQLVMFVGLAFI**ES**LTI**Y**SLMVSFILLGKLPKT | H^+^ | Y | ─ |
|  | *Kuenenia stuttgartiensis* | [91202299](http://www.ncbi.nlm.nih.gov/entrez/query.fcgi?cmd=Retrieve&db=Protein&list_uids=91202299&dopt=GenPept) | 12-83 | IIVAGFTIAVGSIG**P**ALG**E**ARAAAQALSSIAQQPDEANTITRTLFVSMAMI**EST**AI**Y**CFVVAMIVIFANPFW | Na^+^ | Y | ─ |
| **Proteobacteria** |  |  |  |  |  |  |  |
| ***Alpha*** | *Bradyrhizobium* sp. BTAi1 | [148252982](http://www.ncbi.nlm.nih.gov/entrez/query.fcgi?cmd=Retrieve&db=Protein&list_uids=148252982&dopt=GenPept) | 8-79 | IIGAVIAVSIGSIG**P**ALA**E**GRSVAAAMDAIARQPEAAGTISRTLFVGLAMI**ET**MAI**Y**CLVVALLLLFANPYA | H^+^ | Y | ─ |
|  | *Gluconobacter oxydans* | [58040600](http://www.ncbi.nlm.nih.gov/entrez/query.fcgi?cmd=Retrieve&db=Protein&list_uids=58040600&dopt=GenPept) | 9-80 | IVSAAMAVSFGSIG**P**ALA**E**GRAVAAAMDAIARQPESAGTISRTLFVGLAMI**ET**MAIYCLVIALLLLFANPLL | H^+^ | Y | ─ |
|  | *Paracoccus denitrificans* | [119385603](http://www.ncbi.nlm.nih.gov/entrez/viewer.fcgi?db=protein&id=119385603) | 9-77 | QYLGAGLACVGMAG**A**AMGVGNVAGNYLAGALRNPSAAASQTATLFIGMAFA**E**ALGIFSFLVALLLLFAV--- | **H^+^** | N | Y |
|  | *Rhodobacter capsulatus* | [75340080](http://www.ncbi.nlm.nih.gov/entrez/viewer.fcgi?db=protein&id=75340080) | 10-78 | AYIGAGLACTGMGG**A**AVGVGHVVGNFISGALRNPSAAASQTATMFIGIAFA**E**ALGIFSFLVALLLMFAV--- | H^+^ | N | ─ |
|  | *Rhodobacter sphaeroides* | [77464618](http://www.ncbi.nlm.nih.gov/entrez/viewer.fcgi?db=protein&id=77464618) | 10-78 | KFIGAGLATIGLGG**A**GIGVGHVAGNFLAGALRNPSAAPGQMANLFVGIAFA**E**ALGIFSFLIALLLMFAV--- | H^+^ | Y | ─ |
|  | *Rhodobacter sphaeroides* | [126464839](http://www.ncbi.nlm.nih.gov/entrez/query.fcgi?cmd=Retrieve&db=Protein&list_uids=126464839&dopt=GenPept) | 11-79 | ILGAAFAVGIGSLG**P**ALG**E**GRAVAAAMEAIARQPEAAGTLSRTLFVGLAMI**ET**MAI**Y**CLVIALLLLFANPFT | H^+^ | Y | ─ |
|  | *Rhodospirillum rubrum* | [114673](http://www.ncbi.nlm.nih.gov/entrez/viewer.fcgi?db=protein&id=114673) | 7-75 | KMIGAGLAAIGMIGSGIGVGNIWANLIATVGRNPAAKSTVELYGWIGFAVT**E**AIALFALVVALILLFAA--- | **H^+^** | N | Y |
| ***Beta*** | *Burkholderia mallei* | [53716788](http://www.ncbi.nlm.nih.gov/entrez/query.fcgi?cmd=Retrieve&db=Protein&list_uids=53716788&dopt=GenPept) | 10-81 | IAAAALAVSFGAIG**P**ALA**E**GRAVGAAMDAIARQPDASGTVSRTLFVGLAMI**ET**MAI**Y**CLVVALLLLFANPFV | H^+^ | Y | ─ |
|  | *Neisseria meningitidis* | [15793512](http://www.ncbi.nlm.nih.gov/entrez/viewer.fcgi?db=protein&id=15793512) | 5-76 | AIACGLIVALGALG**A**SIGIAMVGSKYLESSARQPELIGPLQTKLFLIAGLI**D**AAFLIGVAIALLFAFVNPFA | H^+^ | N | Y |
|  | *Nitrosomonas eutropha* | [114331984](http://www.ncbi.nlm.nih.gov/entrez/query.fcgi?cmd=Retrieve&db=Protein&list_uids=114331984&dopt=GenPept) | 12-83 | IFTAGLTIAIGSLG**P**ALG**E**GRAAAAAIAAIAQQPDAAPTLSRTLFVSLAMI**EST**AI**Y**CFVVAMILIFANPFW | Na^+^ | Y | ─ |
|  | *Nitrosospira multiformis* | [82702780](http://www.ncbi.nlm.nih.gov/entrez/query.fcgi?cmd=Retrieve&db=Protein&list_uids=82702780&dopt=GenPept) | 12-83 | ILTAGLTISIGVIG**P**ALG**E**GKAVATALTSLAQQPDVAGTIARTLFVGLAII**ES**LAI**Y**CFVVSMILIFANPFW | H^+^ | Y | ─ |
|  | *Polaromonas naphthaleni* | [121605230](http://www.ncbi.nlm.nih.gov/entrez/query.fcgi?cmd=Retrieve&db=Protein&list_uids=121605230&dopt=GenPept) | 12-83 | IVIAGLTTGFGCMG**P**AFA**E**GRAVATALTALAQQPDASATITRTLFVGLAMI**EST**AI**Y**CFVVSMILIFANPFW | Na^+^ | Y | ─ |
|  | *Rhodoferax ferrireducens* | [89899962](http://www.ncbi.nlm.nih.gov/entrez/query.fcgi?cmd=Retrieve&db=Protein&list_uids=89899962&dopt=GenPept) | 12-83 | IVIAGITTGFGCMG**P**ALA**E**GRAVATALTALSQQPDASATITRTLFVGLAMI**EST**AI**Y**CFVVSMILIFANPFW | Na^+^ | Y | ─ |
| ***Gamma*** | *Escherichia coli* | [16131605](http://www.ncbi.nlm.nih.gov/entrez/viewer.fcgi?db=protein&id=16131605) | 10-79 | YMAAAVMMGLAAIG**A**AIGIGILGGKFLEGAARQPDLIPLLRTQFFIVMGLV**D**AIPMIAVGLGLYVMFAVA-- | **H^+^** | Y | Y |
|  | *Azotobacter vinelandii* | [67157244](http://www.ncbi.nlm.nih.gov/entrez/query.fcgi?cmd=Retrieve&db=Protein&list_uids=67157244&dopt=GenPept) | 10-81 | ILGAALAVSFGALG**P**ALA**E**GRAVAAAMDAIARQPEAAGTLSRTLFVGLAMI**ET**MAI**Y**CLVVAVLLLFANPFV | H^+^ | Y | ─ |
|  | *Hahella chejuensis* | [83643799](http://www.ncbi.nlm.nih.gov/entrez/query.fcgi?cmd=Retrieve&db=Protein&list_uids=83643799&dopt=GenPept) | 12-83 | TVAAIIGIALGAML**P**ALAMGKAISSALDALARQPEAEKSITRTLFIGLAMI**ES**LAI**Y**VLVIVLIVLFRNPLL | H^+^ | Y | ─ |
|  | *Legionella pneumophila* | [54298274](http://www.ncbi.nlm.nih.gov/entrez/query.fcgi?cmd=Retrieve&db=Protein&list_uids=54298274&dopt=GenPept) | 12-83 | TVIAAIAIAIGTIG**P**ALAMGRAISHALDALARQPEAEKSITRTLFIGLAMI**ES**LAI**Y**CLVIVLIILFRNPLL | H^+^ | Y | ─ |
|  | *Pseudoalteromonas atlant* | [109898984](http://www.ncbi.nlm.nih.gov/entrez/query.fcgi?cmd=Retrieve&db=Protein&list_uids=109898984&dopt=GenPept) | 12-83 | IITAGLTIGIGVLG**P**SLA**E**GSAVASALKALAQQPDASSTITRTLFVGLAMI**EST**AI**Y**CFVVSMILLFSNPFW | Na^+^ | Y | ─ |
|  | *Psychromonas ingrahamii* | [119944244](http://www.ncbi.nlm.nih.gov/entrez/query.fcgi?cmd=Retrieve&db=Protein&list_uids=119944244&dopt=GenPept) | 12-83 | IIIAGLTTGFGTMG**P**ALA**E**GRAVAAAMASLAQQPDASSTITRTLFVGLAMI**EST**AI**Y**CFVVSMIILFANPFW | Na^+^ | Y | ─ |
|  | *Shewanella frigidimarina* | [114564217](http://www.ncbi.nlm.nih.gov/entrez/query.fcgi?cmd=Retrieve&db=Protein&list_uids=114564217&dopt=GenPept) | 12-83 | IITAGFTITIGVIG**P**SLG**E**GKAVATALSSLAQQPDASATITRTLFVGLAMI**EST**AI**Y**CFVVTMILLFANPFW | Na^+^ | Y | ─ |
|  | *Vibrio alginolyticus* | [60391833](http://www.ncbi.nlm.nih.gov/entrez/viewer.fcgi?db=protein&id=60391833) | 9-80 | AIAVGIIVGLASLGTAIGFALLGGKFLEGAARQPEMAPMLQVKMFIIAGLL**D**AVPMIGIVIALLFTFANPFV | **H^+^** | N | Y |
|  | *Vibrio cholerae* | [9657371](http://www.ncbi.nlm.nih.gov/entrez/viewer.fcgi?db=protein&id=9657371) | 9-80 | AIAVAIIVGLCAVGTAIGFAVLGGKFLEGAARQPEMAPMLQVKMFIIAGLL**D**AVPMIGIVIALLFTFANPFV | **H^+^** | Y | Y |
| ***Delta*** | *Desulfovibrio vulgaris* | [46579330](http://www.ncbi.nlm.nih.gov/entrez/query.fcgi?cmd=Retrieve&db=Protein&list_uids=46579330&dopt=GenPept) | 10-81 | CLAAAIGMAIAAAG**C**GIG**Q**GMGLKAACEGTARNPEAGGKIMVTLILGLAFV**ES**LAI**Y**ALVVNLILLFANPFM | H^+^ | Y | Y |
|  | *Geobacter sulfurreducens* | [39995442](http://www.ncbi.nlm.nih.gov/entrez/query.fcgi?cmd=Retrieve&db=Protein&list_uids=39995442&dopt=GenPept) | 8-79 | MLAAGFGMAIGAFGTGIG**Q**GLAVKNAVEGVSRNPGASGKILTTMMIGLAMI**ES**LAI**Y**VLVVCLIILFANPYK | H^+^ | Y | Y |
|  | *Lawsonia intracellularis* | [94987500](http://www.ncbi.nlm.nih.gov/entrez/viewer.fcgi?val=94987500) | 44-115 | IFGCAIGMALAALG**C**GIG**Q**GLGLKGACEGIARNPEASGKIQVALILGLAFI**ES**LAI**Y**ALVINLIILFANPFV | H^+^ | Y | ─ |
|  | *Syntrophus aciditrophicu* | [85860963](http://www.ncbi.nlm.nih.gov/entrez/query.fcgi?cmd=Retrieve&db=Protein&list_uids=85860963&dopt=GenPept) | 12-83 | IVTAGLCMAVGSIG**P**ALG**E**GNAVKQALTAIAQQPDERNSITRTLFVGLAMI**ES**IAI**Y**CFVISMILIFANPFW | H^+^ | Y | ─ |
| ***Epsilon*** | *Campylobacter jejuni* | [15792265](http://www.ncbi.nlm.nih.gov/entrez/viewer.fcgi?db=protein&id=15792265) | 38-109 | VLAAGLGLGVAALG**G**AIGMGNTAAATIAGTARNPGLGPKLMTTMFIALAMI**E**A**Q**VI**Y**ALVIALIALYANPFI | H^+^ | Y | ─ |
|  | *Helicobacter pylori* | [15645826](http://www.ncbi.nlm.nih.gov/entrez/viewer.fcgi?db=protein&id=15645826) | 32-103 | ILGAMIGLGIAAFG**G**AIGMGNAAAATITGTARNPGVGGKLLTTMFVAMAMI**E**A**Q**VI**Y**TLVFAIIAIYSNPFL | H^+^ | Y | Y |
|  | *Sulfurimonas denitrificans* | [78776630](http://www.ncbi.nlm.nih.gov/entrez/viewer.fcgi?db=protein&id=78776630) | 32-103 | MIAAGLGLGLAALG**G**AIGMGHTAAATIAGTARNPGLGAKLMTTMFIALAMI**E**A**Q**VI**Y**ALVIALIALYANPYL | H^+^ | Y | ─ |
| **Spirochaetes** | *Leptospira borgpetersenii* | [116328618](http://www.ncbi.nlm.nih.gov/entrez/query.fcgi?cmd=Retrieve&db=Protein&list_uids=116328618&dopt=GenPept) | 7-78 | YIGVGIAAGVAILG**A**ALGIGRIGGSATEGISRQPEAGGKIQTAMIIAAALI**E**GAALFALVIAFQAAGTLNEG | H^+^ | Y | ─ |
|  | *Leptospira interrogans* | [24215482](http://www.ncbi.nlm.nih.gov/entrez/viewer.fcgi?db=protein&id=24215482) | 16-87 | YIGVGIAAGVAILG**A**ALGIGRIGGSATEGISRQPEAGGKIQTAMIIAAALI**E**GVSLFALVIAFQAAGTLNEG | H^+^ | Y | Y |
| **Thermotogae** | *Thermotoga maritima* | [15644363](http://www.ncbi.nlm.nih.gov/entrez/viewer.fcgi?val=15644363) | 17-85 | YLGAGLCMGIGAIG**P**GIG**E**GNIGAHAMDAMARQPEMVGTITTRMLLADAVA**ETT**GI**Y**SLLIAFMILLVV--- | Na^+^ | Y | ─ |
|  | *Thermotoga neapolitana* | [24298785](http://www.ncbi.nlm.nih.gov/entrez/viewer.fcgi?val=24298785) | 17-85 | YLGAGLCMGIGAIG**P**GIG**E**GNIGAHAMDAMARQPEMVGTITTRMLLADAVA**ETT**GI**Y**SLLIAFMILLVV--- | Na^+^ | Y | ─ |
|  | *Petrotoga mobilis* | [145622881](http://www.ncbi.nlm.nih.gov/entrez/query.fcgi?cmd=Retrieve&db=Protein&list_uids=145622881&dopt=GenPept) | 27-96 | LLGAGVAMGIGAIG**P**GVG**E**GNIGAHAMDAMARQPEMSGNLTTRMLLAMAVT**EST**GL**Y**SLVVALILLFVLP-- | Na^+^ | Y | ─ |
| **Eukaryota** |  |  |  |  |  |  |  |
| **Fungi** | *Saccharomyces cerevisiae* M | [48428795](http://www.ncbi.nlm.nih.gov/entrez/viewer.fcgi?db=protein&id=48428795) | 8-76 | KYIGAGISTIGLLG**A**GIGIAIVFAALINGVSRNPSIKDTVFPMAILGFALS**E**A**T**GLFCLMVSFLLLFGV--- | H^+^ | N | ─ |
| **Mycetozoa** | *Dictyostelium discoideum* M | [5915722](http://www.ncbi.nlm.nih.gov/entrez/viewer.fcgi?db=protein&id=5915722) | 21-88 | KKVGAGLAAIGLTG**A**GAGVGIVFAAFILAVGMNPNLRGELFKLAMLGFALS**E**AVGLLALMMSFLILYS---- | H^+^ | N | Y |
| **Metazoa** | *Drosophila melanogaster* M | [23172756](http://www.ncbi.nlm.nih.gov/entrez/viewer.fcgi?db=protein&id=23172756) | 70-138 | KFIGAGAATVGVAG**S**GAGIGTVFGSLIIGYARNPSLKQQLFSYAILGFALS**E**AMGLFCLMMAFLLLFAF--- | **H^+^** | N | Y |
|  | *Bos taurus* M | [416684](http://www.ncbi.nlm.nih.gov/entrez/viewer.fcgi?db=protein&id=416684) | 68-136 | KFIGAGAATVGVAG**S**GAGIGTVFGSLIIGYARNPSLKQQLFSYAILGFALS**E**AMGLFCLMVAFLILFAM--- | **H^+^** | N | Y |
| **Viridiplantae** | *Arabidopsis thaliana* C | [6685247](http://www.ncbi.nlm.nih.gov/entrez/viewer.fcgi?db=protein&id=6685247) | 10-81 | VIAAGLAVGLASIG**P**GVG**Q**GTAAGQAVEGIARQPEAEGKIRGTLLLSLAFM**E**ALTI**Y**GLVVALALLFANPFV | **H^+^** | N | Y |
|  | *Arabidopsis thaliana* M | [114152776](http://www.ncbi.nlm.nih.gov/entrez/viewer.fcgi?db=protein&id=114152776) | 17-85 | KLIGAGAATIALAG**A**AIGIGNVFSSLIHSVARNPSLAKQLFGYAILGFALT**E**AIALFALMMAFLILFVF--- | H^+^ | N | Y |
|  | *Porphyra purpurea* C | [1703750](http://www.ncbi.nlm.nih.gov/entrez/viewer.fcgi?db=protein&id=1703750) | 10-81 | VIAAGLAVGLAAIG**P**GIG**Q**GSAAANAVEGIARQPEVEGKIRGTLLLSLAFM**ES**LTI**Y**GLVVALSLLFANPYV | H^+^ | N | ─ |

^a^ Eukaryotic F-ATPase subunits are indicated as M (mitochondrial) or C (chloroplast).

^b^ Gene identification numbers are linked to the corresponding sequences in the NCBI protein database

^c^ Active site residues are indicated indicated in boldface and colored as follows: conserved ion-binding acidic (Glu/Asp) residue - red; other Na^+^ ligands (see Fig. 3) are in purple.. The hydrophobic residue corresponding to Val63 of *Ilyobacter tartaricus* c subunit is shaded yellow. The conserved small (Pro, Gly, Ala, Ser) residue, corresponding to Pro28 of *I. tartaricus* c subunit (see text) is shaded green.

^d^ Predicted cation specificity of the c/K subunit. Ions whose binding has been experimentally studied are shown in bold and colored as in Fig. 5.

^e^ Sequences used to construct sequence logos (Fig. 4) are marked with Y.

^f^ Subunits that have been used to assign cation specificity on Fig. 5 are marked with Y.

**2. V- type ATPase c/K subunits**

| **Phylum, *class*** | **Organism name^a^** | | **gi number^b^** | **Residues** | **2TM sequence (aligned)^c^** | **Ion^d^** | **Fig.4^e^** | **Fig.5^f^** |
| --- | --- | --- | --- | --- | --- | --- | --- | --- |
| **PDB: 2BL2** | ***Enterococcus hirae*** | | [66361544](http://www.ncbi.nlm.nih.gov/entrez/viewer.fcgi?db=protein&id=66361544)_1 | 16-83 | VLAMATATIFSGIG**S**AKGVGMTGEAAAALTTSQPE----KFGQALILQLLPG**TQ**GL**Y**GFVIAFLIFINLGSD | **Na^+^** | ─ | Y |
| **PDB: 2BL2** | ***Enterococcus hirae*** | | [66361544](http://www.ncbi.nlm.nih.gov/entrez/viewer.fcgi?db=protein&id=66361544)_2 | 92-156 | FLGASLPIAFTGLF**S**GIA**Q**GKVAAAGIQILAKKPE----HATKGIIFAAMV**ETY**AILGFVISFLLVLNA--- |  | ─ |  |
| **Archaea** |  | |  |  |  |  |  |  |
| **Nanoarchaeota** | *Nanoarchaeum equitans* | | [41615011](http://www.ncbi.nlm.nih.gov/entrez/viewer.fcgi?val=41615011) | 3-69 | ALASALAIGLAAFG**S**AIA**Q**GLAASAAAAATSEKPD----LFGKMLIFAALP**ETQ**AI**Y**GLVIAYLLSKIV--- | Na^+^ | Y | Y |
| **Crenarchaeota** | *Aeropyrum pernix* | | [118431830](http://www.ncbi.nlm.nih.gov/entrez/viewer.fcgi?db=protein&id=118431830) | 38-102 | AIGAGLAVGLAGIG**G**GYAVGVAGAAATSSITEKPE----MFGRSLLFVVLG**E**GIAI**Y**GLLIALLLLLVV--- | H^+^ | Y | Y |
|  | *Caldivirga maquilingensis* | | [159042457](http://www.ncbi.nlm.nih.gov/entrez/viewer.fcgi?db=protein&id=159042457) | 43-103 | YLGAGLAFGLAAGG**A**GIGMGIAGAAIASASIEKR-----DLLIFFLVLAFV**ET**IAL**Y**GFVALILLR------ | H^+^ | Y | Y |
|  | *Cenarchaeum symbiosum* | | [118195146](http://www.ncbi.nlm.nih.gov/entrez/viewer.fcgi?db=protein&id=118195146) | 36-99 | LLGAGLAFGLAAGG**A**GIGLGYVGSAGLAVISENPA----LQSKVFIFIGMV**ES**IAI**Y**GIVMMFIILGQ---- | H^+^ | Y | Y |
|  | *Hyperthermus butylicus* | | [124027658](http://www.ncbi.nlm.nih.gov/entrez/viewer.fcgi?db=protein&id=124027658) | 55-119 | AIAAALAMGLSAIG**A**GIALGRTGSAASAAVAEKPE----VSGKLLIYLVLG**E**GIAI**Y**GLLVAILIIFTG--- | H^+^ | Y | ─ |
|  | *Ignicoccus hospitalis* | | [156937475](http://www.ncbi.nlm.nih.gov/entrez/viewer.fcgi?db=protein&id=156937475) | 49-113 | AVGAGLALLGGTIG**A**GYALGATGAAGIAVISEKPE----EFGRVLLFIGIA**ET**PAI**Y**GIAIAIVILFAI--- | H^+^ | Y | ─ |
|  | *Pyrobaculum aerophilum* | | [18312157](http://www.ncbi.nlm.nih.gov/entrez/viewer.fcgi?db=protein&id=18312157) | 26-87 | YIGAGLAVGLAGLG**A**GIGVGIAGAAAMSALVEKPQ----ERVWYLIFLALA**E**AIAI**Y**GLLVSILLI------ | H^+^ | Y | Y |
|  | *Staphylothermus marinus* | | [126466069](http://www.ncbi.nlm.nih.gov/entrez/viewer.fcgi?db=protein&id=126466069)_1 | 10-77 | YAGAAFALMGGLIG**S**SIGMGKAGSAGSATLAEDPK----QFRNVFLLASLPM**TQ**TF**Y**GLIILIQYIGYINGH | H^+^ | ─ | ─ |
|  | *Staphylothermus marinus* | | [126466069](http://www.ncbi.nlm.nih.gov/entrez/viewer.fcgi?db=protein&id=126466069)_2 | 89-155 | ILGLGLAVAGAELF**S**AWF**Q**GVICASGISELPRTKGA---VTFSTMILAVYV**E**LIGILGMVFGFLGLSLIG-- |  | ─ |  |
|  | *Sulfolobus acidocaldarius* | | [74053555](http://www.ncbi.nlm.nih.gov/entrez/viewer.fcgi?db=protein&id=74053555) | 36-101 | NIGAGLAVGLAAIG**A**GVAVGTAAAAGIGVLTEKRE----MFGTVLIFVAIG**E**GIAV**Y**GIIFAVLMLFAGI-- | H^+^ | Y | Y |
| **Euryarchaeota** |  | |  |  |  |  |  |  |
| ***Archaeoglobi*** | *Archaeoglobus fulgidus* | | [11498760](http://www.ncbi.nlm.nih.gov/entrez/viewer.fcgi?db=protein&id=11498760) | 11-75 | AVGAGLAVGLAGIG**A**GLG**E**SGIGAAAVGATAEDRG----FFGLGILFTVIP**ET**IVIFGLVIAFILMFAF--- | H^+^ | Y | Y |
| ***Halobacteria*** | *Haloarcula marismortui* | | [55379717](http://www.ncbi.nlm.nih.gov/entrez/viewer.fcgi?db=protein&id=55379717) | 26-86 | SAGAALAVGLAALG**S**GYA**E**RGIGAAAVGAIAEDES----MFGRGLILTVLP**ET**LVILALVVVFVV------- | H^+^ | Y | ─ |
|  | *Halobacterium salinarum* | | [1487875](http://www.ncbi.nlm.nih.gov/entrez/viewer.fcgi?db=protein&id=1487875) | 25-89 | KAAAALAVGLAALA**A**GYA**E**RGIGSAAVGAIAEDPD----LFGTGLILTVLP**ET**LVILALVVVFVVPTPF--- | H^+^ | Y | ─ |
| ***Methanobacteria*** | *Methanobrevibacter smithii* | | [148642499](http://www.ncbi.nlm.nih.gov/entrez/query.fcgi?cmd=Retrieve&db=Protein&list_uids=148642499&dopt=GenPept)_1 | 12-79 | AIGAGVAIGFAGLG**S**GLG**Q**GMAAAGSVGAVAEDND----MFARGIIFSALP**ETQ**AI**Y**GFLIAILLLVFSGLL | Na^+^ | ─ | ─ |
|  | *Methanobrevibacter smithii* | | [148642499](http://www.ncbi.nlm.nih.gov/entrez/query.fcgi?cmd=Retrieve&db=Protein&list_uids=148642499&dopt=GenPept)_2 | 93-160 | AIGVGAAIGFAGLG**S**GMG**Q**GIAASSSVGAIVEDND----MFARGIIFSALP**ETQ**AI**Y**GFLIAILLMVFGGIL |  | ─ |  |
|  | *Methanosphaera stadtmanae* | | [84489931](http://www.ncbi.nlm.nih.gov/entrez/query.fcgi?cmd=Retrieve&db=Protein&list_uids=84489931&dopt=GenPept)_1 | 13-80 | AIGAGVAVGFAALG**S**GIG**Q**GIASSASVGAVAEDSS----MFAQGLVFTAIP**ETQ**AI**Y**GFLIAILLLVFSGIM | Na^+^ | ─ | Y |
|  | *Methanosphaera stadtmanae* | | [84489931](http://www.ncbi.nlm.nih.gov/entrez/query.fcgi?cmd=Retrieve&db=Protein&list_uids=84489931&dopt=GenPept)_2 | 93-160 | AIGAGAAVGFGGLG**S**GMG**Q**GIASSASVGAVVEEPG----MFAQGLVFTAIP**ETQ**AI**Y**GFLIAILLLVFGGIL |  | ─ |  |
|  | *Methanothermobacter thermauto.* | | [15678977](http://www.ncbi.nlm.nih.gov/entrez/query.fcgi?cmd=Retrieve&db=Protein&list_uids=15678977&dopt=GenPept)_1 | 12-79 | AIGAGVAVGFAGLG**S**GLG**Q**GIAAAESVGAVAENSD----MFARGIIFSTLP**ETQ**AI**Y**GFLIAILLLMVFSGL | **Na^+^** | ─ | Y |
|  | *Methanothermobacter thermauto.* | | [15678977](http://www.ncbi.nlm.nih.gov/entrez/query.fcgi?cmd=Retrieve&db=Protein&list_uids=15678977&dopt=GenPept)_2 | 93-159 | AVGAGAAIGFAGLG**S**GMG**Q**GITSASSVGAVVEDPD----MFARGIIFSALS**ETQ**AI**Y**GFLIAILL-MVFGGI |  | ─ |  |
| ***Methanococci*** | *Methanocaldococcus jannaschii* | | [15668394](http://www.ncbi.nlm.nih.gov/entrez/query.fcgi?cmd=Retrieve&db=Protein&list_uids=15668394&dopt=GenPept)_1 | 9-76 | AVGAGLAVGIAGLG**S**GIGAGITGASGAGVVAEDPN----KFGTAIVFQALP**QTQ**GL**Y**GFLVAILILFVFKTV | **Na^+^** | ─ | Y |
|  | *Methanocaldococcus jannaschii* | | [15668394](http://www.ncbi.nlm.nih.gov/entrez/query.fcgi?cmd=Retrieve&db=Protein&list_uids=15668394&dopt=GenPept)_2 | 80-147 | AMFAAGLAAGLAGL**S**AIG**Q**GIAASAGLGAVAEDNS----IFGKAMVFSVLP**ETQ**AI**Y**GLLIAILLLVGVFKG |  | ─ |  |
|  | *Methanocaldococcus jannaschii* | | [15668394](http://www.ncbi.nlm.nih.gov/entrez/query.fcgi?cmd=Retrieve&db=Protein&list_uids=15668394&dopt=GenPept)_3 | 155-220 | AALGAGFAVGFAGL**S**GIG**Q**GITAAGAIGATARDPD----AMGKGLVLAVMP**ET**FAIFGLLIAILIMLMIK-- |  | ─ |  |
|  | *Methanococcus maripaludis* | | [45358603](http://www.ncbi.nlm.nih.gov/entrez/query.fcgi?cmd=Retrieve&db=Protein&list_uids=45358603&dopt=GenPept)_1 | 11-78 | AIGAGLAVGIAGLG**S**GIGAGITGASGAGVVAEDPN----KFGTAIVFQALP**QTQ**GL**Y**GFLVAILILFVFKSA | Na^+^ | ─ | Y |
|  | *Methanococcus maripaludis* | | [45358603](http://www.ncbi.nlm.nih.gov/entrez/query.fcgi?cmd=Retrieve&db=Protein&list_uids=45358603&dopt=GenPept)_2 | 82-149 | AMLAAGIGTGLAGL**S**AIG**Q**GIASAAGLGAVAEDDG----IFGKAMVFSVLP**ETQ**AI**Y**GLLVAILLLVGVFAS |  | ─ |  |
|  | *Methanococcus maripaludis* | | [45358603](http://www.ncbi.nlm.nih.gov/entrez/query.fcgi?cmd=Retrieve&db=Protein&list_uids=45358603&dopt=GenPept)_3 | 156-222 | AALGAGLAVGFAGL**S**GIG**Q**GITAAGAIGATARDPD----AMGKGLVLAVMP**ET**FAIFGLLIAILIMLGIMF- |  | ─ |  |
| ***Methanomicrobia*** | | *Methanococcoides burtonii* | [91773219](http://www.ncbi.nlm.nih.gov/entrez/viewer.fcgi?db=protein&id=91773219) | 18-79 | AIGAGLAVGLTGLA**S**GIA**E**KDIGAAAIGAMAENES----LFGKGLILTVIP**ET**IVIFGLVVALLIK------ | H^+^ | Y | Y |
|  | *Methanosaeta thermophila* | | [116754902](http://www.ncbi.nlm.nih.gov/entrez/viewer.fcgi?db=protein&id=116754902) | 14-81 | AVGAGLATGLAGIG**A**GVG**E**QGIGAAVVGVVAEEPG----FLGKGLFLMLLP**ET**LIIFGLAVSLILMFAWSPF | **H^+^** | Y | Y |
|  | *Methanosarcina acetivorans* | | [20092947](http://www.ncbi.nlm.nih.gov/entrez/viewer.fcgi?val=20092947) | 19-82 | ALGAALAITVTGLA**S**AWA**E**KEIGTAAIGAMAENEG----LFGKGLILTVIP**ET**IVIFGLVVALLINSA---- | H^+^ | Y |  |
|  | *Methanosarcina barkeri* | | [72395089](http://www.ncbi.nlm.nih.gov/entrez/viewer.fcgi?db=protein&id=72395089) | 18-81 | AVGASIAIALTGIA**S**AIA**E**KDIGTAAIGAMAENEG----LFGKGLILTVIP**ET**IVIFGLVVALLINSA---- | **H^+^** | ─ | Y |
|  | *Methanosarcina mazei* | | [21226886](http://www.ncbi.nlm.nih.gov/entrez/viewer.fcgi?db=protein&id=21226886) | 18-80 | ALGAAIAIAVTGLA**S**AIA**E**KDIGTAAIGAMAENEG----LFGKGLILTVIP**ET**IVIFGLVVALLINQ----- | **H^+^** | ─ | Y |
|  | *Methanospirillum hungatei* | | [88603036](http://www.ncbi.nlm.nih.gov/entrez/query.fcgi?cmd=Retrieve&db=Protein&list_uids=88603036&dopt=GenPept)_1 | 2-69 | AIGAGIAVGCSAIG**S**GIGVGIVGSAASGVISERSE----KFGMALVFTAIP**QTQ**AI**Y**GLLIAILILQAGGFL | Na^+^ | ─ | ─ |
|  | *Methanospirillum hungatei* | | [88603036](http://www.ncbi.nlm.nih.gov/entrez/query.fcgi?cmd=Retrieve&db=Protein&list_uids=88603036&dopt=GenPept)_2 | 83-150 | VAVAAGLAVGLAGF**S**AIG**Q**GIAASSGVANTAEKPE----MFGKGVVFSAVC**ETQ**AI**Y**GLLIAVLMLALTGIL |  | ─ |  |
|  | *Methanospirillum hungatei* | | [88603036](http://www.ncbi.nlm.nih.gov/entrez/query.fcgi?cmd=Retrieve&db=Protein&list_uids=88603036&dopt=GenPept)_3 | 162 230 | GLVGAGLAVGLAGF**S**AIG**Q**GITCSSGIAATARNPG----AIGRSLVFAAMS**ET**FAIFGLLVAILILFGLGLF |  | ─ |  |
| ***Methanopyri*** | *Methanopyrus kandleri* | | [20094449](http://www.ncbi.nlm.nih.gov/entrez/query.fcgi?cmd=Retrieve&db=Protein&list_uids=20094449&dopt=GenPept)_1 | 9-77 | AIGAGLAAGVAGVG**S**GIG**Q**GIAAAAGAGAVAEDEA----TFGKAIVFSVLP**ETQ**AI**Y**GLLTAILIMVGIGLL | Na^+^ | Y | ─ |
|  | *Methanopyrus kandleri* | | [20094449](http://www.ncbi.nlm.nih.gov/entrez/query.fcgi?cmd=Retrieve&db=Protein&list_uids=20094449&dopt=GenPept)_2 | 90-157 | AALGAGLAVGLAGI**S**GIG**Q**GIAAASGIGAVLKDEA----LFGRAIVYAVLP**ETQ**AI**Y**GLLVAIIIMVGSGLL |  | Y |  |
|  | *Methanopyrus kandleri* | | [20094449](http://www.ncbi.nlm.nih.gov/entrez/query.fcgi?cmd=Retrieve&db=Protein&list_uids=20094449&dopt=GenPept)_3 | 171-238 | AAMGAGLAVGLAGT**S**GIG**Q**GIAAASGIHGVLRKEE----LFGRLIVFSVLP**ETQ**AI**Y**GLLTAILIANFVGLL |  | Y |  |
|  | *Methanopyrus kandleri* | | [20094449](http://www.ncbi.nlm.nih.gov/entrez/query.fcgi?cmd=Retrieve&db=Protein&list_uids=20094449&dopt=GenPept)_4 | 251-318 | AAMGAGLAVGLAGT**S**GIG**Q**GIAAASGIKSLIEEEG----VFGRAIVFSVLP**ETQ**AI**Y**GLLVAILTLFSLLKP |  | Y |  |
|  | *Methanopyrus kandleri* | | [20094449](http://www.ncbi.nlm.nih.gov/entrez/query.fcgi?cmd=Retrieve&db=Protein&list_uids=20094449&dopt=GenPept)_5 | 327-394 | AALGMGLAVGIAGT**S**GIG**Q**GIAAASGIAGVLRKEE----LFGRLIVFSVLP**ETQ**AI**Y**GLLTAILAMFFLGAG |  | Y |  |
|  | *Methanopyrus kandleri* | | [20094449](http://www.ncbi.nlm.nih.gov/entrez/query.fcgi?cmd=Retrieve&db=Protein&list_uids=20094449&dopt=GenPept)_6 | 402-470 | AAVGAGLAVGFGGT**S**GIG**Q**GIAAASGIRAMIERAE----LFVRGMVLSVLP**ETR**AI**Y**GLLIAILALFMMKSG |  | Y |  |
|  | *Methanopyrus kandleri* | | [20094449](http://www.ncbi.nlm.nih.gov/entrez/query.fcgi?cmd=Retrieve&db=Protein&list_uids=20094449&dopt=GenPept)_7 | 477-544 | ALIGAGLAVGLVGV**S**GIG**Q**GFTAATGAATLVKNEG----FFGRAIIFSVLP**ETQ**AI**Y**GLLTAILIMMFAGIL |  | Y |  |
|  | *Methanopyrus kandleri* | | [20094449](http://www.ncbi.nlm.nih.gov/entrez/query.fcgi?cmd=Retrieve&db=Protein&list_uids=20094449&dopt=GenPept)_8 | 558-625 | AAVGAGLAVGLAGS**S**AIG**Q**GIAAAAGVGASAEKEE----LFGRSVVFSILP**ETQ**SI**Y**GLLIGILLAVFAMKA |  | Y |  |
|  | *Methanopyrus kandleri* | | [20094449](http://www.ncbi.nlm.nih.gov/entrez/query.fcgi?cmd=Retrieve&db=Protein&list_uids=20094449&dopt=GenPept)_9 | 634-701 | AALGAGLAVGIAGF**S**GIG**Q**GIAAAAGIGALKRDPG----SFGRSLIFSILP**ETR**SI**Y**GLLVAILVMVGLGLM |  | Y |  |
|  | *Methanopyrus kandleri* | | [20094449](http://www.ncbi.nlm.nih.gov/entrez/query.fcgi?cmd=Retrieve&db=Protein&list_uids=20094449&dopt=GenPept)_10 | 714-781 | AALGAGLAIGLAGL**S**GVG**Q**GVTAATGISNVVKDPG----MFGRSLLFSVFP**ETQ**AI**Y**GLLIAILIMMFAGIL |  | Y |  |
|  | *Methanopyrus kandleri* | | [20094449](http://www.ncbi.nlm.nih.gov/entrez/query.fcgi?cmd=Retrieve&db=Protein&list_uids=20094449&dopt=GenPept)_11 | 794-861 | AALGAGIAVGMAGT**S**GIG**Q**GISAAAGARATAEDPG----NFGRSIVFSILP**ETQ**SI**Y**GLLAGILALTPVLTG |  | Y |  |
|  | *Methanopyrus kandleri* | | [20094449](http://www.ncbi.nlm.nih.gov/entrez/query.fcgi?cmd=Retrieve&db=Protein&list_uids=20094449&dopt=GenPept)_12 | 873-940 | IGIGAGLAVGVAGT**S**GIG**Q**GIAAAGGTGALAERTE----MFARSLILSILP**ETR**SI**Y**GLLIAILSMSLTGVL |  | Y |  |
|  | *Methanopyrus kandleri* | | [20094449](http://www.ncbi.nlm.nih.gov/entrez/query.fcgi?cmd=Retrieve&db=Protein&list_uids=20094449&dopt=GenPept)_13 | 953-1020 | AAVAAGIAVGFAGL**S**GIG**Q**GITAARGSASMVRREQ----VFGKSLVFSVLP**ETQ**AI**Y**GLLTAILIVFAALAA |  | Y |  |
| ***Thermococci*** | *Pyrococcus horikoshii* | | [3258424](http://www.ncbi.nlm.nih.gov/sutils/blink.cgi?pid=3258424&quq=1171791)_1 | 11-79 | SLGMALGAGLAGAA**S**SFGVGIAGAAAAGAVAEDER----NFRNALILEGLPM**TQ**SI**Y**GLITLFLIGMAAGII | Na^+^ | ─ | Y |
|  | *Pyrococcus horikoshii* | | [3258424](http://www.ncbi.nlm.nih.gov/sutils/blink.cgi?pid=3258424&quq=1171791)_2 | 98-162 | ILFGAGLLVGLTGF**S**AIP**Q**GIIAGSGIGAVSKNPR----TFTQNLIFAAMA**ET**MAIFGLVGAIILIMSL--- |  | ─ |  |
|  | *Thermococcus kodakarensis* | | [57641533](http://www.ncbi.nlm.nih.gov/entrez/viewer.fcgi?val=57641533)_1 | 8-75 | VLGAALAAGIAGIA**S**AFGIGIAGAAAAGAVAEDEK----NFKNALILEGLPM**TQ**SI**Y**GLITLFLIMLSAGII | Na^+^ | ─ | Y |
|  | *Thermococcus kodakarensis* | | [57641533](http://www.ncbi.nlm.nih.gov/entrez/viewer.fcgi?val=57641533)_2 | 90-161 | ILFGAGLTVGLTGL**S**AIP**Q**GIIASAGIGAAAKNPK----TFTQGVIFAAMA**ETT**AIFGLVGALIMIATGVGF |  | ─ |  |
| ***Thermoplasmata*** | *Ferroplasma acidarmanus* | | [126008295](http://www.ncbi.nlm.nih.gov/entrez/viewer.fcgi?db=protein&id=126008295) | 10-75 | ALAASIAIAGGLIGTGMA**Q**QGIGAAGMGIIAEKPE----KFGQVLFFFVIP**ET**LWIIGFVLGIILLLHVI-- | H^+^ | ─ | Y |
|  | *Picrophilus torridus* | | [48477566](http://www.ncbi.nlm.nih.gov/entrez/viewer.fcgi?db=protein&id=48477566) | 11-76 | AVAASIAIAGGLIGTGMA**Q**QGIGAAGMGIIAEKPE----KFGQVLFFFVIP**ET**LWIIGFILGIILLLHVI-- | H^+^ | ─ | Y |
| **Bacteria** |  | |  |  |  |  |  |  |
| ***Bacteroidetes*** | *Bacteroides fragilis* | | [52216885](http://www.ncbi.nlm.nih.gov/entrez/viewer.fcgi?val=52216885)_1 | 9-76 | YIGIAVMVGLSGIG**S**AYGVTIAGNAAIGALKKNDS----AFGNFLVLTALPG**TQ**GL**Y**GFAGYFMFQTIFGIL | Na^+^ | ─ | Y |
|  | *Bacteroides fragilis* | | [52216885](http://www.ncbi.nlm.nih.gov/entrez/viewer.fcgi?val=52216885)_2 | 88-153 | VLGAGIALGLVALF**S**AIR**Q**GQVCANGIAAIGQGHN----VFSNTLILAVFP**E**L**Y**AIVALAATFLIGSALA-- |  | ─ |  |
|  | *Porphyromonas gingivalis* | | [34541427](http://www.ncbi.nlm.nih.gov/entrez/query.fcgi?cmd=Retrieve&db=Protein&list_uids=34541427&dopt=GenPept)_1 | 7-74 | YLGIALMVALTGIG**S**AIGVTICGNTTVGAMKKNPD----SLGLYIGLSALPS**SQ**GL**Y**GFVGFFMASGLITKL | Na^+^ | ─ | ─ |
|  | *Porphyromonas gingivalis* | | [34541427](http://www.ncbi.nlm.nih.gov/entrez/query.fcgi?cmd=Retrieve&db=Protein&list_uids=34541427&dopt=GenPept)_2 | 88-155 | IFFAGLALGVVGLM**S**AIR**Q**AQVCANGIQAIGGGHN----VFGATMVMAVFP**E**L**Y**AILALLVSILIFGSVPGM |  | ─ |  |
| ***Chlamydiae*** | *Chlamydia trachomatis* | | [15605025](http://www.ncbi.nlm.nih.gov/entrez/query.fcgi?cmd=Retrieve&db=Protein&list_uids=15605025&dopt=GenPept)_1 | 6-73 | VVGPVLAMALAMIG**S**AVGCGMAGVASHAVMSRIDE----GHGKIIGLSAMPS**SQ**SI**Y**GLIFMLLLNDAIKDG | Na^+^ | ─ | Y |
|  | *Chlamydia trachomatis* | | [15605025](http://www.ncbi.nlm.nih.gov/entrez/query.fcgi?cmd=Retrieve&db=Protein&list_uids=15605025&dopt=GenPept)_2 | 80-141 | GIVMGIAVGSALLL**S**AFM**Q**GKCCVSAIQAYARSSA----IYGKSFASIGIV**ES**FALFAFVFALLLF------ |  | ─ |  |
|  | *Chlamydophila pneumoniae* | | [15835627](http://www.ncbi.nlm.nih.gov/entrez/viewer.fcgi?val=15835627)_1 | 6-73 | VVGPALVLGLAMIG**S**AIGCGMAGVASHAVMSRIDE----GHGKLIGMSAMPS**SQ**SI**Y**GFILMLLMQAAIKNG | Na^+^ | ─ | ─ |
|  | *Chlamydophila pneumoniae* | | [15835627](http://www.ncbi.nlm.nih.gov/entrez/viewer.fcgi?val=15835627)_2 | 80-141 | GIAIGLSVGAALLV**S**SVM**Q**GKCCVSGIQAYARSSS----IYGKCYAAIGIV**ES**FSLFAVVFALLLL------ |  | ─ |  |
|  | *Protochlamydia amoebophila* | | [46447310](http://www.ncbi.nlm.nih.gov/entrez/query.fcgi?cmd=Retrieve&db=Protein&list_uids=46447310&dopt=GenPept)_1 | 5-72 | MVGPAMALGLSSMG**C**SIGCWIAGSASHAAMSRTEE----GHGKFIGMAAAPS**SQ**VI**Y**GFLLMLQMSRAIQAG | Na^+^ | ─ | ─ |
|  | *Protochlamydia amoebophila* | | [46447310](http://www.ncbi.nlm.nih.gov/entrez/query.fcgi?cmd=Retrieve&db=Protein&list_uids=46447310&dopt=GenPept)_2 | 79-140 | AIAIGIFSGLAIGL**S**SIY**Q**GKVCATGIQASLKQPS----VYGKCFAAIGII**ES**FALFAFVFALLII------ |  | ─ |  |
| ***Deinococcus-Thermus*** | *Deinococcus geothermalis* | | [94986150](http://www.ncbi.nlm.nih.gov/entrez/viewer.fcgi?db=protein&id=94986150) | 41-102 | AIGAGLALGLGALGTGVA**Q**ARIGSSLVGAVAEDPS----KAGSLLLYFLIP**ET**LVIFGFLALFILA------ | H^+^ | Y | ─ |
|  | *Deinococcus radiodurans* | | [15805723](http://www.ncbi.nlm.nih.gov/entrez/viewer.fcgi?val=15805723) | 40-101 | AIGKGLALGLGALGTGVA**Q**ARIGSSLVGAAAEDPS----KLGQLLLVFLLP**ET**LVIFGFLALFLIR------ | H^+^ | Y | Y |
|  | *Thermus thermophilus* | | [1510114](http://www.ncbi.nlm.nih.gov/entrez/viewer.fcgi?val=1510114) | 35-99 | AVGMGLAVGLAALGTGVA**Q**ARIGAAGVGAIAEDRS----NFGTALIFLLLP**ET**LVIFGLLIAFILNGRL--- | H^+^ | Y | Y |
| ***Firmicutes*** |  | |  |  |  |  |  |  |
| ***Bacilli*** | *Enterococcus faecalis* | | [29376061](http://www.ncbi.nlm.nih.gov/entrez/viewer.fcgi?val=29376061)_1 | 16-83 | VLGMAMATIFAGIG**S**AKGVGFTGEAAAALTTEQPE----KFGQALILQLLPG**TQ**GL**Y**GFVIAFLIYINLGND | **Na^+^** | ─ | Y |
|  | *Enterococcus faecalis* | | [29376061](http://www.ncbi.nlm.nih.gov/entrez/viewer.fcgi?val=29376061)_2 | 92-157 | YFVAALPIAFAGLF**S**GIA**Q**GRVAAAGIQILAKKPE----HATKGIIYAAMV**ET**YAILGFVISFLLVLNVK-- |  | ─ |  |
|  | *Streptococcus pneumoniae* | | [15901175](http://www.ncbi.nlm.nih.gov/entrez/viewer.fcgi?val=15901175)_1 | 18-85 | ALGIVLAVGLSGMG**S**AYGVGKAGQSAAALLKEQPE----KFASALILQLLPG**TQ**GL**Y**GFVIGILIWLQLTPE | Na^+^ | ─ | ─ |
|  | *Streptococcus pneumoniae* | | [15901175](http://www.ncbi.nlm.nih.gov/entrez/viewer.fcgi?val=15901175)_2 | 94-158 | YFFVALPIAIVGYF**S**AKH**Q**GNVAVAGMQILAKRPK----EFMKGAILAAMV**ETY**AILAFVVSFILTLRV--- |  | ─ |  |
| ***Clostridia*** | *Caloramator fervidus* | | [87082843](http://www.ncbi.nlm.nih.gov/entrez/viewer.fcgi?db=protein&id=87082843)_1 | 21-88 | LLGAALAALMAGIG**S**AKGVGIVGQSAAGLITEEPE----KFGQSLILQVIPG**TQ**GF**Y**GFITALIVLSRIGLL | **Na^+^** | ─ | Y |
|  | *Caloramator fervidus* | | [87082843](http://www.ncbi.nlm.nih.gov/entrez/viewer.fcgi?db=protein&id=87082843)_2 | 103-169 | LLMACLPMAFVGYS**S**AIS**Q**GKTAAAGIQILAKRPE----KMFNGVIYAVMV**ET**YAVVALITSILMIVNIKI- |  | ─ |  |
|  | *Clostridium perfringens* | | [18310624](http://www.ncbi.nlm.nih.gov/entrez/query.fcgi?cmd=Retrieve&db=Protein&list_uids=18310624&dopt=GenPept)_1 | 19-86 | AFGIALAVGMSGIG**S**AKGVGIVGEAAAGLVTEEPE----KFGKALVLELLPG**TQ**GL**Y**GFVIGFLVFNQISNG | Na^+^ | ─ | Y |
|  | *Clostridium perfringens* | | [18310624](http://www.ncbi.nlm.nih.gov/entrez/query.fcgi?cmd=Retrieve&db=Protein&list_uids=18310624&dopt=GenPept)_2 | 96-163 | LLFACLPIAIAGLW**S**GIS**Q**GKAAAAGIQILAKRPE----HNTKGIIFAAMV**ETY**ALLGFVISFLLVNGASVI |  | ─ |  |
|  | *Thermoanaerobacter ethanolicus* | | [76588877](http://www.ncbi.nlm.nih.gov/entrez/viewer.fcgi?val=76588877)_1 | 11-78 | LLGAAMAVFLPGIG**S**AKGVGMVGEAAAGVVTEDPS----KFSQTLILQALPG**TQ**GI**Y**GLLTGFVVMQRIGIL | Na^+^ | ─ | Y |
|  | *Thermoanaerobacter ethanolicus* | | [76588877](http://www.ncbi.nlm.nih.gov/entrez/viewer.fcgi?val=76588877)_2 | 93-160 | VFAACLPIAIVGLL**S**AIS**Q**ARAAAAGVGIVAKRPE----ELAKGITYAAMV**ETY**AVLALLASILMLFGLKLA |  | ─ |  |
| ***Mollicutes*** | *Acholeplasma laidlawii* | | [162448015](http://www.ncbi.nlm.nih.gov/entrez/viewer.fcgi?val=162448015) | 82-149 | YLGAAISTGASALG**A**GIAVAAAAPAAIGAISENPK----NLGKSLIFVALG**E**GVAI**Y**GMLISILILNTIPAI | H^+^ | Y | ─ |
| **Fusobacteria** | *Fusobacterium nucleatum* | | [19705061](http://www.ncbi.nlm.nih.gov/entrez/query.fcgi?cmd=Retrieve&db=Protein&list_uids=19705061&dopt=GenPept)_1 | 18-85 | VLGAVIAVLLSGIG**S**AKGVGIAGQAAAGLIIDEPE----KFGKAMVLQLLPG**TQ**GL**Y**GFVIGLLIMFRLTSQ | Na^+^ | ─ | ─ |
|  | *Fusobacterium nucleatum* | | [19705061](http://www.ncbi.nlm.nih.gov/entrez/query.fcgi?cmd=Retrieve&db=Protein&list_uids=19705061&dopt=GenPept)_2 | 94-160 | LLMAGLPVGLVGLK**S**ALY**Q**GQVAVAGINILAKNEA----HQTKGIVLAVMV**ETY**AVLAFVMSLLLLNQVQF- |  | ─ |  |
| **Proteobacteria** |  | |  |  |  |  |  |  |
| ***Alpha*** | *Stappia aggregata* | | [118434482](http://www.ncbi.nlm.nih.gov/entrez/viewer.fcgi?db=protein&id=118434482)_1 | 9-77 | WFGLFAPVALGAIG**S**AWGCALGGSAAIGAMLDSDG----GYGRFIGVSLMPS**SQ**VI**Y**GIVIMFSLQQPTIDA | H^+^ | ─ | ─ |
|  | *Stappia aggregata* | | [118434482](http://www.ncbi.nlm.nih.gov/entrez/viewer.fcgi?db=protein&id=118434482)_2 | 83-149 | LFGIGLLSGVTMLYTGIR**Q**GEVLASAIHASKAKPE----IFGISLAPAAVL**E**GFSVFALVFALVLSGSIPA- |  | ─ |  |
| ***Gamma*** | *Beggiatoa* sp. PS | | [153870236](http://www.ncbi.nlm.nih.gov/entrez/viewer.fcgi?db=protein&id=153870236) | 84-148 | IIGIGIPTAFASLG**A**AIAVGPVGAASLAVIAEKPE----MFGRTLVYLGLA**E**GIAI**Y**GLVVTILLLGKL--- | H^+^ | Y | ─ |
|  | *Nitrosococcus oceani* | | [77165551](http://www.ncbi.nlm.nih.gov/entrez/viewer.fcgi?db=protein&id=77165551) | 87-148 | LLAIGLPTAVATVA**A**GLAVGAVGSSALAAISEKPE----LFGRTLIYLGLA**E**GIAI**Y**GVVVTILMLGKI--- | H^+^ | Y | ─ |
| ***Delta*** | *Anaeromyxobacter dehalogenans* | | [86157624](http://www.ncbi.nlm.nih.gov/entrez/viewer.fcgi?val=86157624) | 7-71 | TLAAALAVGVTALATAWV**Q**SRIGSAGAGALAEKPE----VRGAVIVMLAIP**ET**LVILGFVVAVLILTGG--- | H^+^ | Y | ─ |
|  | *Geobacter uraniumreducens* | | [148262502](http://www.ncbi.nlm.nih.gov/entrez/viewer.fcgi?db=protein&id=148262502) | 7-70 | GFAAALAIGLPAIATGWA**Q**SKIGSAGAGAVAEKPE----LTGTMLIMLAIP**ET**MVILGFVVAAMILYL---- | H^+^ | Y |  |
| **Spirochaetes** | *Borrelia burgdorferi* | | [2687983](http://www.ncbi.nlm.nih.gov/sutils/blink.cgi?pid=2687983&quq=15835627)_1 | 5-76 | LIGVNSALTISAIG**S**ALGMGAAGSAAIGAWKRCYMQPAPFGKLLIVFVSAPL**TQ**II**Y**GYILMNTLYEVMMQT | Na^+^ | ─ | Y |
|  | *Borrelia burgdorferi* | | [2687983](http://www.ncbi.nlm.nih.gov/sutils/blink.cgi?pid=2687983&quq=15835627)_2 | 81-144 | LLGAGIGGGFAIAV**S**GFA**Q**GKAAAGACDAFSETGKG----FATYLLVLGLI**ES**VALFVMVFLMIFKFV---- |  | ─ |  |
|  | *Treponema pallidum* | | [15639421](http://www.ncbi.nlm.nih.gov/entrez/viewer.fcgi?val=15639421)_1 | 5-76 | MFGAAAVLGISAVG**S**ALGLALAGQGTIGSWKRCYLNPAPFNKILLAFAGAPL**TQ**TI**Y**GFLLMKAMFSSEKDP | Na^+^ | ─ | Y |
|  | *Treponema pallidum* | | [15639421](http://www.ncbi.nlm.nih.gov/entrez/viewer.fcgi?val=15639421)_2 | 79-140 | LLGAGVACGLGIAA**S**ALS**QG**RAAAAGADALAETGKG----FSQYLTIVGLC**ET**VALLVMVFGIINC------ |  | ─ |  |
| **Thermotogae** | *Thermotoga neapolitana* | | [24298772](http://www.ncbi.nlm.nih.gov/entrez/viewer.fcgi?db=protein&id=24298772) | 79-143 | LLAVALSTGLAAVG**A**GVAV**G**MTGAASIGAISEKPEM----LGRTLIYVGLG**E**GIVI**Y**GLIISIIILGRL--- | H^+^ | Y | ─ |
|  | *Thermotoga* sp. RQ2 | | [161405925](http://www.ncbi.nlm.nih.gov/entrez/viewer.fcgi?db=protein&id=161405925) | 78-142 | LMAVALSTGLAAVG**A**GIAV**G**MTGAASVGAISEKPEL----LGRTLIYVGLA**E**GIVI**Y**GLIVSIMILGRL--- | H^+^ | Y | ─ |
| **Eukaryota** |  | |  |  |  |  |  |  |
| **Fungi** | *Saccharomyces cerevisiae* | | [137481](http://www.ncbi.nlm.nih.gov/entrez/viewer.fcgi?db=protein&id=137481)_1 | 14-81 | AIGCASAIIFTSLG**A**AYGTAKSGVGICATCVLRPDL----LFKNIVPVIMAGIIAI**Y**GLVVSVLVCYSLGQK | **H^+^** | ─ | Y |
|  | *Saccharomyces cerevisiae* | | [137481](http://www.ncbi.nlm.nih.gov/entrez/viewer.fcgi?db=protein&id=137481)_2 | 90-157 | QLGAGLSVGLSGLA**A**GFAIGIVGDAGVRGSSQQPRL----FVGMILILIFA**E**VLGL**Y**GLIVALLLNSRATQD |  | ─ |  |
| **Mycetozoa** | *Dictyostelium discoideum* | | [1718094](http://www.ncbi.nlm.nih.gov/entrez/viewer.fcgi?db=protein&id=1718094)_1 | 33-100 | AMGVTAALVFTVMG**A**AYGTAKASVGISNMGVMKPDL----VIKAFIPVIFAGVIAI**Y**GLIICVILVGGIKPN | **H^+^** | ─ | Y |
|  | *Dictyostelium discoideum* | | [1718094](http://www.ncbi.nlm.nih.gov/entrez/viewer.fcgi?db=protein&id=1718094)_2 | 111-178 | DLGAGLTVGLCGLA**A**GMAIGIVGDSGVRAFGQQPKL----YVIMMLILIFS**E**ALGL**Y**GLIIGILLSSVSDTY |  | ─ |  |
| **Metazoa** | *Drosophila melanogaster* | | [137478](http://www.ncbi.nlm.nih.gov/entrez/viewer.fcgi?db=protein&id=137478)_1 | 18-85 | VMGAASAIIFSALG**A**AYGTAKSGTGIAAMSVMRPEL----IMKSIIPVVMAGIIAI**Y**GLVVAVLIAGALEEP | **H^+^** | ─ | Y |
|  | *Drosophila melanogaster* | | [137478](http://www.ncbi.nlm.nih.gov/entrez/viewer.fcgi?db=protein&id=137478)_2 | 96-159 | HLGAGLAVGFSGLA**A**GFAIGIVGDAGVRGTAQQPRL----FVGMILILIFA**E**VLGL**Y**GLIVAIYLYTK---- |  | ─ |  |
|  | *Bos taurus* | | [137477](http://www.ncbi.nlm.nih.gov/entrez/viewer.fcgi?db=protein&id=137477)_1 | 16-83 | VMGASAAMVFSALG**A**AYGTAKSGTGIAAMSVMRPEM----IMKSIIPVVMAGIIAI**Y**GLVVAVLIANSLNDG | **H^+^** | ─ | Y |
|  | *Bos taurus* | | [137477](http://www.ncbi.nlm.nih.gov/entrez/viewer.fcgi?db=protein&id=137477)_2 | 92-155 | QLGAGLSVGLSGLA**A**GFAIGIVGDAGVRGTAQQPRL----FVGMILILIFA**E**VLGL**Y**GLIVALILSTK---- |  | ─ |  |
| **Viridiplantae** | *Arabidopsis thaliana* | | [27923954](http://www.ncbi.nlm.nih.gov/entrez/viewer.fcgi?db=protein&id=27923954)_1 | 17-84 | FLGAAAALVFSCMG**A**AYGTAKSGVGVASMGVMRPEL----VMKSIVPVVMAGVLGI**Y**GLIIAVIISTGINPK | **H^+^** | ─ | Y |
|  | *Arabidopsis thaliana* | | [27923954](http://www.ncbi.nlm.nih.gov/entrez/viewer.fcgi?db=protein&id=27923954)_2 | 96-163 | HLSSGLACGLAGLS**A**GMAIGIVGDAGVRANAQQPKL----FVGMILILIFA**E**ALAL**Y**GLIVGIILSSRAGQS |  | ─ |  |

^a^ Eukaryotic F-ATPase subunits are indicated as M (mitochondrial) or C (chloroplast).

^b^ Gene identification numbers are linked to the corresponding sequences in the NCBI protein database

^c^ Active site residues are indicated indicated in boldface and colored as follows: conserved ion-binding acidic (Glu/Asp) residue - red; other Na+ ligands (see Fig. 3) are in purple.. The hydrophobic residue corresponding to Val63 of *Ilyobacter tartaricus* c subunit is shaded yellow. The conserved small (Pro, Gly, Ala, Ser) residue, corresponding to Pro28 of *I. tartaricus* c subunit (see text) is shaded green.

^d^ Predicted cation specificity of the c/K subunit. Ions whose binding has been experimentally studied are shown in bold and colored as in Fig. 5.

^e^ Sequences used to construct sequence logos (Fig. 4) are marked with Y.

^f^ Subunits that have been used to assign cation specificity on Fig. 5 are marked with Y.
