# Supplementary material for: Evolutionary primacy of sodium bioenergetics
Source: Biol Direct. 2008 Apr 1;3:13. doi: 10.1186/1745-6150-3-13 (PMC2359735; doi:10.1186/1745-6150-3-13)
Supplement: Additional file 2 — Multiple alignment of the catalytic subunits of F- and V-type ATPases used for the construction of the tree in Fig. 5 [file 1745-6150-3-13-S2.doc]

**GI number Alignment Species names as in the Figure 5**

2605819 GKVISVRGSIVDVLFEPPVYTLLRAIAIEVLTQLDAHHVRGIALTPTEGLARGMAVEDT B_Methanosarcina_barkeri_F

20091272 GSVVSVRGSIVDIRFEPPIYSLLRAIAIEVLTQLDSHRVRGIALTPTEGLSRGTLVEDT B_Methanosarcina_acetivorans_F

61219617 GRVARVIGPVVDVEFPPEIYNALHVLTLEVAQHLGDGLVRTISMQPTDGLIRQAPVTDT B_Streptomyces_coelicolor

15608450 GRVVRVTGPVVDVEFPPELFNALHALTLEVAQHLGDNLVRTISLQPTDGLVRGVEVIDT B_Mycobacterium_tuberculosis

3913128 GKVVQVIGPVVDVEFEPKIKDGLKTLFMEVAQHIGEHRVRAIAMGPTDGLVRGQEVEYL B_Aquifex_aeolicus

2493023 GKIIQVLGPVVDVEFEPAIFEALDILVLEVAAHLGGNRVRAIAMDMTEGLVRNQVIKAR B_Helicobacter_pylori

81467688 GKIKQIIGSVLDIEFEPEIYNALEIIIAEVQTHIGGKAVRAIALSSTDGLIRGQEVSNT B_Leptospira_interrogans

3913124 GFIVSIMGPVVDVKFPPDIYNALEVVVLEVEQLIGDGVVRTVAMDSTDGLTKGLEVVDT B_Thermotoga_maritima

66826095 GVVHQVIGAVVDVYFPPYINDALIVLVLEVAQHLGDGIVRCVALDITDGLGRGALVLNT E_Dictyostelium_discoideum_M

53713460 GHISQVIGPVVDVYFEPSIHDALEILIVEVQQHIGENTVRTVAMDSTDGLQRGMKVFPT B_Bacteroides_fragilis_F

114573 GKIVQIIGAVVDVEFPPSVYDALNVLVLEVQQQLGGGVVRCIVMGSSDGLRRGVEVVNT B_Vibrio alginolyticus

14547933 GKIVQIIGAVVDVEFPPSVYDALNVLVLEVQQQLGGGVIRAIVMGSSDGLRRGMTVQNT B_Vibrio cholerae

15677764 GKIVQIIGAVVDVEFPPRVYDALKLLTLEVQQLLGDGVVRAIAMGSSDGLKRGMTVSNT B_Neisseria_meningitidis

16131600 GKIVQVIGAVVDVEFPPRVYDALEVLVLEVQQQLGGGIVRTIAMGSSDGLRRGLDVKDL B_Escherichia_coli

25452812 GKISQIIGPVVDVDFPPSILDALTVLVLETQQHLGEERVRTIAMEGTDGLVRGMSAVNT B_Chlorobium_tepidum

81830486 GKIVQVIGAVVDVEFPPNILNALDILVCEVAQHLGDNIVRTIAMDATEGLVRGMEASDT B_Desulfovibrio_vulgaris

81832140 GKISQVIGAVIDVEFEPGIYNALRVLVLEVAQHLGENAVRTIAMDSTDGLVRGQAVLDT B_Geobacter_sulfurreducens

22326673 GRVCQVIGAIVDVRFEPPIMTSLEVLVLEVSHHLGQNVVRTIAMDGTEGLVRGRKVLNT E_Arabidopsis_thaliana_M

114543 GRIVAVIGAVVDVQFDPPILNALEVLVLEVAQHLGESTVRTIAMDGTEGLVRGQKVLDS E_Bos_taurus_M

47606749 GKIVAVIGAVVDVQFDPPILNALEVLVLEVAQHLGENTVRTIAMDGTEGLVRGQKVLDT E_Drosophila_melanogaster_M

83592562 GTITQVTGAVVDVKFEPSILSALETLVLEVAQHLGESVVRTIAMDSTEGLVRGQQVTST B_Rhodospirillum_rubrum

2493025 GKVTQVIGAVVDVQFEPAILNALETLVLEVAQHLGENTVRTIAMDATEGLVRGAAVSDT B_Paracoccus_denitrificans

114570 GKITQVIGPVIDAQFPPRIYNALKVVTCEVQQLLGDNQVRAVAMSSTDGLVRGMDVVDT B_Synechocystis_PCC_6803

6686269 GRIAQIIGPVLDVAFPPNIYNALVVVTCEVQQLLGNNRVRAVAMSATEGLKRGMDVVDM E_Arabidopsis thaliana_C

11465725 GSVTQIIGPVLDIAFPPKVFNALKVITCEVQQLLGDNKVRAVSMSSTEGLKRGVEVVDT E_Porphyra_purpurea_C

1703680 GKVVQVIGPVVDVKFQPKLNNAVNILVIEVAQQLGDDIVRCIAMDSTDGLMRNQEAVDT B_Acetobacterium_woodii

15673746 GKITQIIGPVVDVEFGPEINNALIVITLEVALELGDGAVRTIAMESTDGLTRGLEVLDT B_Lactococcus_lactis

29377093 GKIVQVIGPVVDVEFSPDINNALVVVVLEVALELGDGVIRSIAMESTDGLQRGMEVIDT B_Enterococcus_faecalis_F

1168575 GKIVQVIGPVVDVEFSPDINNALVVVVLETALELGDGVIRTIAMESTDGLQRGMEVIDT B_Enterococcus_hirae_F

114571 GRVIQVMGPVVDVKFEPAIYNALKILTLEVALHLGDDTVRTIAMASTDGLIRGMEVIDT B_Bacillus_PS3

14916961 GHITQVMGPVVDVKFKPEINNALKVVTLEVALHLGDDSVRTVAMGSTDGLVRGTEALDT B_Bacillus_pseudofirmus

20137589 GKIVQVIGPVVDIKFDPDIYNSIEILIAEVEQHVGDDIVRTIAMEGTDGLKRGMEAVNT B_Clostridium_acetobutylicum

22266799 GILTQIIGPVVDVSFDPKIYNAIKILVAEVQQHLGNNVVRAICMDGSEGLQRGMEVIDT B_Ilyobacter_tartaricus

1352033 GVITQIIGPVVDVTFEPRIYNALKILVAEVQQHLGNSVVRAVAMDATDGLQRGMEVVDT B_Propionigenium_modestum

77964181 GKVVQIIGPVLDIKFSPNLLNAIEIIVAEVAQHIGDDTVRCIAMSSTDGLVRGIDAIDT B_Clostridium_paradoxum

2662066 GKFVQIVGAVVDVRFDPNLLNALEILVVEVAQHIGDDVVRCIAMSSTDGLVRGVDAVDT B_Ruminococcus_albus

137464 GAIYSVSGPVVIAENMCAMYELVKVLVGEVIRIDGDKATIQV-YEETAGLTVGDPVLRT E_Saccharomyces_cerevisiae_VA

92090652 GFVLSVSGPVVIANQLAAMYELVRVLVGEIIRLEEDTATIQV-YEETSGLTVGDPVLRT E_Dictyostelium_discoideum_VA

3334404 GYVRKVSGPVVVADGMAAMYELVRVLIGEIIRLEGDSATIQV-YEETAGLTVNDPVLRT E_Arabidopsis_thaliana_VA

124053334 GYVHGVSGPVVTACDMAAMYELVRVLVGEIIRLEGDMATIQV-YEETSGVSVGDPVLRT E_Bos_taurus_VA

93141332 GRVFAVSGPVVTAEAMSAMYELVRVLVGEIIRLEGDMATIQV-YEETSGVTVGDPVLRT E_Drosophila_melanogaster_VA

25009559 GKIEYISGPVVKAELPARLYELVFVLFGEVVRIQGEKAFIQV-YEDTTGLKPGEPVERT A_Pyrobaculum_aerophilum_VA

126352990 GRILVVNGPVIKAELPAKLYELVFVLFGEVVRVQGENAFIQV-YEDTTGIRPGEPVVRT A_Caldivirga maquilingensis_VA

41614899 NRIISINGPLVIAKGKFSIFEVVRVLIGEVIGIENDKAYIQV-YEDTNGLKVGEPVFNT A_Nanoarchaeum_equitans_VA

126008291 GSIYSISGPVVIATDLGKMFDVVRVLVGEVIKIVGDKFTIQV-YEDTSGLKPGEPVYST A_Ferroplasma_acidarmanus_VA

48477562 GSIYSVSGPVVIAQDIAKMFDVVRVLIGEIIRISGNKATIQV-YEDTSGLRPGEKVYST A_Picrophilus_torridus_VA

12229704 GEIESVSGPVVTATGLAQMNDVVYVLMGEVIEIEGDVTTIQV-YEETSGIGPGQPVDNT A_Halobacterium salinarium_VA

2493099 GVIASVSGPVVTARGLARMNDVVYVLMGEVIEIEGDLTTIQV-YEETSGVGPGEPVEST A_Haloferax_volcanii

12585484 GVVQSIAGPAVIAKGMAKMYDIVRVLVGEIIRLDGDTAFVQV-YEDTAGLTVGEPVETT B_Deinococcus_radiodurans_VA

32172455 GVIQKIAGPAVIAKGMARMYDICKVLVGEIIRLDGDTAFVQV-YEDTSGLKVGEPVVST B_Thermus_thermophilus_VA

116754898 GRVKRVAGPVVQAVGLASMYDLVLVLMSEVIGISGDKHIIQV-YEDTSGIKPGEPVKET A_Methanosaeta_thermophila_VA

12585404 GEIYRISGPLVVAEGLARMYDLCKVLMGEVVGLVGQKVLIQV-YEDTEGVKPGDKVENT A_Archaeoglobus_fulgidus_VA

91773223 GEIYRVAGPVVTVIGIPRMYDVVKVLMGEVIRIKGEQATVQV-YEDTSGLKPGEPVMNT A_Methanococcoides_burtonii_VA

22002078 GEIYRVAGPVVTAIGLAKMYDLCKVLMGEVIQIVGDKTIIQV-YEETGGVRPGEPCVTT B_Methanosarcina_mazei_VA

114520 GEIYRVSGPVVTAIGLAKMYDLVKVLMGEVIQILGPKTIIQV-YEETAGIKPGEPCVST B_Methanosarcina_barkeri_VA

29376065 GKIVKVSGPLILAENMASIQDICHVVIGEIIEMRGDVASIQV-YEETTGIGPGEPVIST B_Enterococcus_faecalis_VA

1171780 GKIIKVSGPLVMAENMASIQDMCLVVIGEIIEMRQDVASIQV-YEETSGIGPGEPVRST B_Enterococcus hirae_VA

76795559 GIITKVSGPLVVAEGLAKMFDVVKVLIGEIIEIRGERVSIQV-YEETSGLGPGDPVVST B_Thermoanaerobacter_ethanolicus_VA

110798759 GKIIKVSGPLVVAEGMANVYDVVKVLIGEIIEMRGDKASIQV-YEETSGIGPGDPVITT B_Clostridium_perfringens_VA

87082847 GRIIKVSGPLVVAEGMAKMFDVCRVLIGEIIEMRGDKASIQV-YEETSGLGIGEPVETT B_Caloramator fervidus_VA

12585422 GRIIRVTGPLVVADGMAKMYEVVRVLIGEIIRLEGDKAVIQV-YEETAGVRPGEPVVGT A_Pyrococcus_horikoshii_VA

57641537 GRIIRVTGPLVVADGMAKMYEVVRVLIGEIIRLEGDKAVIQV-YEETAGIRPGEPVEGT A_Thermococcus_kodakarensis_VA

12585563 GKIIKIAGPVVVAEGMAQMYEVVKVLTGEIIQLHDDKAVIQV-YEETSGIKPGEPVVGT A_Methanocaldococcus_jannaschii_VA

45358607 GKIIKISGPVVVAEGMSQMFEVVKVLTGEIIQLTEDEAIIQV-YEETAGIKPGEGVEGT A_Methanococcus_maripaludis_VA

84489927 GNIIKIAGPVIIGDGMTQIHEMVRVLIGEIIELEGDTATVQV-YEETAGIKPGEKIEST A_Methanosphaera_stadtmanae_VA

12585397 GRIIKIAGPVIIAEGMSQMYEMVKVLIGEIIELEGDTATIQV-YEETAGIKPGETVERT A_M_thermautotrophicus_VA

118195150 GKIVWVSGPAVKADGMAKMYETVTVLIGEVIRLTGDVAFIQV-YESTSGLKPGEPVEGT A_Cenarchaeum_symbiosum_VA

12585522 GSIVRISGPLVVAEGMAQMYEMVYVLIGEITRIRGDRAFIQV-YESTSGLKPGEPVVGT A_Aeropyrum_pernix_VA

74053565 GRVVRVNGPLVVADGMAQMFEVVEVLVGEITRIEGDRAYIQV-YEATDGIKPGEKAYRT A_Sulfolobus_acidocaldarius_VA

53714024 GTVSGVIANMVTLVVDVAQNEICYILMAEVIKVVGTHVYVQV-FESTRGLKVGAEAEFT B_Bacteroides_fragilis_VA

12585442 GYVVEAYGNLLRVHVDVRQGEVAYVLKAEIIEVVGDEVKIQV-FEETQGISRGALVTFS B_Chlamydia_trachomatis_VA

12585416 GKVVGVNGNLVTIEVEVSMNEVLFVLKAEVIRIRGNEVDAQV-FELTKGISVGDLVEFT B_Borrelia_burgdorferi_VA

12585431 GIVSAVNGNMVSVTFEVSLNEVGYVLKAEIIRVRGREAQLQV-FEITRGVSVGDRVEFT B_Treponema_pallidum_VA

**Continued on the next page**

**GI number Alignment**  **Species names as in the Figure 5**

2605819 GGPLKAPVGREILSRMFDVFGNTIDKPPSDIQWRSIHQTPPLMRRSTTSEIFETGIKAI B_Methanosarcina_barkeri_F

20091272 GGPLKAPVGRGILSRMFDVFGNPIDAPPSNVRWRTIHQAPPLIRRSTRSEIFETGIKVI B_Methanosarcina_acetivorans_F

61219617 GAAISVPVGDFTKGKVFNTLGEVLNDEQYTGERWPIHRKAPFDELESKTEMFETGVKVI B_Streptomyces_coelicolor

15608450 GRSISVPVGEGVKGHVFNALGDCLDGYGEKFEHWSIHRKPPFEELEPRTEMLETGLKVV B_Mycobacterium_tuberculosis

3913128 GGPIKIPVGKEVLGRIFNVAGQPIDGPVEAKEYWPMFRNPPLVEQSTKVEILETGIKVI B_Aquifex_aeolicus

2493023 GKMIEVPVGEEVLGRIFNVVGESIDEPLKPSLTWPIHRKAPFEQQSTKTEMFETGIKVI B_Helicobacter_pylori

81467688 GKPISVPVGDATLGRIFNVLGKTIDPAITVKETRPIHRAAPFDELTSKTEVFETGIKVI B_Leptospira_interrogans

3913124 GAPITAPVGKEVLGRILNVIGEPVDGEIKAKERWPIHRPAPLVEQSTEIEILETGIKVI B_Thermotoga_maritima

66826095 GSPLMVPVGQATLGRIMNVIGEPIDGPIPATEKRPIWRAPPFADLAPSASILETGIKVI E_Dictyostelium_discoideum_M

53713460 GGPITMPVGEQIKGRLMNVVGDSIDKELNRDGAYSIHRDPPFEDLTTVQEVLFTGIKVI B_Bacteroides_fragilis_F

114573 GAPISVPVGTKTLGRIMNVLGDAIDGEVGAEEVYSIHRSAPYEEQSNEIALLETGVKVI B_Vibrio alginolyticus

14547933 GAPISVPVGTKTLGRIMNVLGDAIDGDIGAEEVYSIHRPAPYEEQSSATELLETGVKVI B_Vibrio cholerae

15677764 GAPITVPVGKGTLGRIVDVLGTPVDGPIDTDKSRAIHQAAPFDELSSTTELLETGIKVI B_Neisseria_meningitidis

16131600 EHPIEVPVGKATLGRIMNVLGEPVDGEIGEEERWAIHRAAPYEELSNSQELLETGIKVI B_Escherichia_coli

25452812 GKPIQVPVGGEVLGRMLNVVGDPIDGPVPAKKTYSIHRAAPFDELSTKTEMFETGIKVI B_Chlorobium_tepidum

81830486 GKPIMVPVGKASLGRIMNVVGRPVDGPINADKSLPIHRAAPFTEQNTKVELLETGIKVV B_Desulfovibrio_vulgaris

81832140 GKQISVPVGRKTLGRILNVIGEPVDGPVNAEKEYGIHREAPAFVDQSTKVEAFTTGIKV B_Geobacter_sulfurreducens

22326673 GAPITVPVGRATLGRIMNVLGEPIDGEIKTEHYLPIHRDAPLVDLATGQEILATGIKVV E_Arabidopsis_thaliana_M

114543 GAPIRIPVGPETLGRIMNVIGEPIDGPIKTKQFAAIHAEAPFVEMSVEQEILVTGIKVV E_Bos_taurus_M

47606749 GYPIRIPVGAETLGRIINVIGEPIDGPIDTDKTAAIHAEAPFVQMSVEQEILVTGIKVV E_Drosophila_melanogaster_M

83592562 GGPITVPVGPQVLGRIMNVIGEPVDGPVVTAQRYPIHRQAPFAEQATETEILVTGIKVI B_Rhodospirillum_rubrum

2493025 GGPITVPVGNATLGRILNVIGEPVDGDVSKAEARAIHQPAPFAAQSTESQILVTGIKVI B_Paracoccus_denitrificans

114570 GAPISVPVGTGTLGRIFNVLGEPVDGPVPAGETFPIHRPAPLVDLETKPQVFETGIKVI B_Synechocystis_PCC_6803

6686269 GNPLSVPVGGATLGRIFNVLGEPVDGPVDTRTTSPIHKSAPFIELDTKLSIFETGIKVV E_Arabidopsis thaliana_C

11465725 GAPISVPVGTNTLGRIFNVLGEPVDGPVSSESTLPIHRPAPFTKLETKPSIFETGIKVV E_Porphyra_purpurea_C

1703680 GSAIQVPVGKATLGRMFNVLGEPIDFDTKDVVMHPIHRHPPFEEQQTQPEMFETGIKVV B_Acetobacterium_woodii

15673746 GKAVSVPVGEATLGRVFNVLGDVIDEFAADAERNPIHKKAPFDELSTANEVLVTGIKVV B_Lactococcus_lactis

29377093 GKSISVPVGKDTLGRVFNVLGDTIDPFPADAERSGIHKKAPFDELSTSNEILETGIKVI B_Enterococcus_faecalis_F

1168575 GKAISVPVGKDTLGRVFNVLGDTIDSFPEDAERSEIHKKAPFDELSTSTEILETGIKVI B_Enterococcus_hirae_F

114571 GAPISVPVGQVTLGRVFNVLGEPIDDIPADARRDPIHRPAPFEELATEVEILETGIKVV B_Bacillus_PS3

14916961 GAPISVPVGEATLGRVFNVLGEAIDPVAADVKRDPIHREAPFEELSTTTEILETGIKVV B_Bacillus_pseudofirmus

20137589 GKPISVPVGENVLGRLFNVLGQTIDGDMNADKYYPIHRPAPFEEQSVQPEMFETGIKVI B_Clostridium_acetobutylicum

22266799 GAPITVPVGKAVLGRILNVLGEAIDEELNAEEFAPIHREAPFEDQGTDVEIFETGIKVI B_Ilyobacter_tartaricus

1352033 GPAITVPVGKAVLGRILNVLGEPVDGEVKAEEYAPIHREAPFEDQGTEKEVFETGIKVV B_Propionigenium_modestum

77964181 GNAISVPVGRKTLGRIFNVLGDPVDPEVEDAPKLPIHRPAPFEDQETATEILETGIKVV B_Clostridium_paradoxum

2662066 GRAISVPVGRETLSRMFNLLGDPVDPAPETAERWEIHREPPYEEQTAANEILETGIKVI B_Ruminococcus_albus

137464 GKPLSVELGPGLMETIYDGIQRPLKSDFTLYHTWPVRVPRPVTEKLSADYPLLTGQRVL E_Saccharomyces_cerevisiae_VA

92090652 HKPLTVELGPGIMNNIFDGIQRPLNKQLTMVHNWPVRSARPVIEKLPCNYPLLTGQRVL E_Dictyostelium_discoideum_VA

3334404 HKPLSVELGPGILGNIFDGIQRPLKKSYTMLQSWPVRTPRPVASKLAADTPLLTGQRVL E_Arabidopsis_thaliana_VA

124053334 GKPLSVELGPGIMGAIFDGIQRPLSEKFSMVQVWPVRQVRPVTEKLPANHPLLTGQRVL E_Bos_taurus_VA

93141332 GKPLSVELGPGIMGSIFDGIQRPLKTKHTMLQVWPVRQPRPVTEKLPANHPLLTGQRVL E_Drosophila_melanogaster_VA

25009559 GEPLSAWLGPTIIGKIYDGVQRPLRVEVKMWHKWPVRRPRPFKEKLPPVEPLITGVRTV A_Pyrobaculum_aerophilum_VA

126352990 GEMLSAWLGPGIIGQVYDGVQRPLKIKVKMWHKWPVRRPRPFQEKLPPSDPLITGIRVI A_Caldivirga maquilingensis_VA

41614899 GKPLTIELGPGLLANIFDGLGRPLKKPIKLYHEWPIRKPRPYKEKLDYNYPFITGTRVL A_Nanoarchaeum_equitans_VA

126008291 GKPLSVELGPGLLKSIYDGIQRPLDYDIKLKQIWPVRQARKVFLKFAPEIPLITGQRVI A_Ferroplasma_acidarmanus_VA

48477562 GKPLSVELGPGLLSSIYDGIQRPLDYEIKLKQIWPVREARRVFHKFPPEIPLITGQRVI A_Picrophilus_torridus_VA

12229704 GEPLTVDLGPGMLDSIYDGVQRPLDEEIQMHQEWPVRRQRPTVDKQTPTEPLVSGQRIL A_Halobacterium salinarium_VA

2493099 GEPLTVDLGPGMMDAIYDGVQRPLDEEITMHQEWPVRQARPAAEKKTPREPLVSGQRIL A_Haloferax_volcanii

12585484 GLPLSVELGPGMLNGIYDGIQRPLDTKLRLAHYWPVRAPRPVQKKLDPSLPFLTGMRIL B_Deinococcus_radiodurans_VA

32172455 GLPLAVELGPGMLNGIYDGIQRPLETELKMYHTWPVRRARPVQRKLDPNTPFLTGMRIL B_Thermus_thermophilus_VA

116754898 GGPLVAQLGPGILTQIYDGVQRPLPSELTMLQKWPVRQARPVVRKLPPTIPLRTGQRVI A_Methanosaeta_thermophila_VA

12585404 GMPLSVELGPGLIRNIYDGVQRPLPTELKLYHKWPVRIPRPYVEKLPPVVPLITGQRIL A_Archaeoglobus_fulgidus_VA

91773223 GLPLSVELGPGLLESIYDGIQRPLPTEISMMQKWPVRGPRPVAKKLMPTKPLITGQRIL A_Methanococcoides_burtonii_VA

22002078 GMSLAVELGPGLLSSIYDGVQRPLHTELQMMQRWPVRKPRPVKRKLTPEKPLVTGQRIL B_Methanosarcina_mazei_VA

114520 GSSLSVELGPGLLSSIYDGVQRPLHTELQMMQRWPVRRPRPVKAKLTPTRPLVTGMRIL B_Methanosarcina_barkeri_VA

29376065 GEPLSVELAPGLIAEMFDGIQRPLDESFSMMQKWPVRRGRPILEKLSPKVPMVTGQRVI B_Enterococcus_faecalis_VA

1171780 GEALSVELGPGIISQMFDGIQRPLDKELTMMQKWPVRRGRPIKQKLNPDVPMITGQRVI B_Enterococcus hirae_VA

76795559 GEPLSVELGPGMLEGIFDGIQRPLDVEITMMQKWPVRKARPYKEKLPPEIPMPTGQRVI B_Thermoanaerobacter_ethanolicus_VA

110798759 GEPLSVELGPGLIESMFDGIQRPLDKNLTLMQKWPVRKGRPYARKLNPVEPMTTGQRVI B_Clostridium_perfringens_VA

87082847 GEPLSVELGPGLIEAMFDGIQRPLDVNLTMMQKWPVRKGRPYKSKITPEEPLLTGQRVI B_Caloramator fervidus_VA

12585422 GASLSVELGPGLLTSIYDGIQRPLEKELKMYQRWPVRVKRPYKEKLPPEVPLITGQRVI A_Pyrococcus_horikoshii_VA

57641537 GSSLSVELGPGLLTAMYDGIQRPLEEELKMYHRWPVRVKRPYKQKLPPEVPLITGQRTI A_Thermococcus_kodakarensis_VA

12585563 GAPLSVELGPGMLRAMYDGIQRPLTKEITMMQKWPVRKPRPYKEKLPPEIPLITGQRVE A_Methanocaldococcus_jannaschii_VA

45358607 GAPLSVELGPGMLKAMYDGIQRPLNKEIMMMQKWPVRKPRPSKGKQAPVIPLITGQRVE A_Methanococcus_maripaludis_VA

84489927 GGPLSVELGPGILKSIYDGIQRPLDETVQMMQVWPVRVGRPYTNKLDPDVPLITGQRAQ A_Methanosphaera_stadtmanae_VA

12585397 GGPLSVELGPGILGSIFDGIQRPLEEKIQMLQKWPVRKGRPYKKKLDPDVPLVTGQRAQ A_M_thermautotrophicus_VA

118195150 GNPLSVLLGPGIIGQIYDGIQRPLKIPLKMYHRWPVRQPRSYHTKYDPTVPLITGQRVI A_Cenarchaeum_symbiosum_VA

12585522 GAPLSVELGPGLLGTIYDGVQRPLPVEIRMHQRWPVRIPRPFKEKLEPQLPLITGVRII A_Aeropyrum_pernix_VA

74053565 GSLLSVELGPGLMGGIFDGLQRPLDKELKLYQRWPVRIPRPFKEKLEPTEPLLTGTRVV A_Sulfolobus_acidocaldarius_VA

53714024 GHMLEVTLGPGMLSKNYDGLQNDLDVEVNMIQKWPVKRAMTYKEKPRPFKLLETGVRVI B_Bacteroides_fragilis_VA

12585442 GHLLEAELGPGLLQGIFDGLQNRLELEFTMVQKWPIKQAFLEGEKVPSHEIMDVGLRVL B_Chlamydia_trachomatis_VA

12585416 DKLLTVELGPGLLTQVYDGLQNPLPHNITMSFHWPVKVPITYKERLIPSEPMLTQTRII B_Borrelia_burgdorferi_VA

12585431 GDLLSVELGPGLLGQVYDGLQNPLPHELTMAFRWPVKRPVHYAERLKPTEPLVTSIRTI B_Treponema_pallidum_VA

**Continued on the next page.**

**GI number Alignment**  **Species names as in the Figure 5**

2605819 DVLVPLERGGKAGLFGGAGVGKTVLLTEMIHNVVKQHQGVSIFCGIGERCREGEELYRD B_Methanosarcina_barkeri_F

20091272 DVLVPLERGGKAGLFGGAGVGKTVLLTEMIHNVVRQQKGVSIFCGIGERCREGEELYRD B_Methanosarcina_acetivorans_F

61219617 DLLTPYVKGGKIGLFGGAGVGKTVLIQEMIYRVANNHDGVSVFAGVGERTREGNDLIDE B_Streptomyces_coelicolor

15608450 DLLTPYVRGGKIALFGGAGVGKTVLIQEMINRIARNFGGTSVFAGVGERTREGNDLWVE B_Mycobacterium_tuberculosis

3913128 DLLQPIIKGGKVGLFGGAGVGKTVLMQELIHNIARFHEGYSVVVGVGERTREGNDLWLE B_Aquifex_aeolicus

2493023 DLLAPYSKGGKVGLFGGAGVGKTVIIMELIHNVAYKHNGYSVFAGVGERTREGNDLYFE B_Helicobacter_pylori

81467688 DLLAPYIKGGKTGLFGGAGVGKTVLIQELINNIAKQHGGFSVFAGVGERTREGNDLWRE B_Leptospira_interrogans

3913124 DLLAPFPKGGKIGFFGGAGVGKTVLVMELIRNIAIEHKGFSVFAGVGERTREGNELWLE B_Thermotoga_maritima

66826095 DLLAPYSRGGKIGLFGGAGVGKTVLIQELINNIAKAHGGFSVFTGVGERTREGNDLYHE E_Dictyostelium_discoideum_M

53713460 DLLEPYSKGGKIGLFGGAGVGKTVLIMELINNIAKKHNGFSVFAGVGERTREGNDLLRE B_Bacteroides_fragilis_F

114573 DLICPFAKGGKIGLFGGAGVGKTVNMMELINNIALQHSGLSVFAGVGERTREGNDFYYE B_Vibrio alginolyticus

14547933 DLICPFAKGGKIGLFGGAGVGKTVNMMELINNIALQHSGLSVFAGVGERTREGNDFYHE B_Vibrio cholerae

15677764 DLLCPFAKGGKVGLFGGAGVGKTVNMMELINNIAKAHSGLSVFAGVGERTREGNDFYHE B_Neisseria_meningitidis

16131600 DLMCPFAKGGKVGLFGGAGVGKTVNMMELIRNIAIEHSGYSVFAGVGERTREGNDFYHE B_Escherichia_coli

25452812 DLLEPYSRGGKTGLFGGAGVGKTVLIMELINNIAKQQSGYSVFAGVGERTREGNDLWHE B_Chlorobium_tepidum

81830486 DLLIPFPKGGKMGLFGGAGVGKTVILMEMINNIAKQHGGISVFAGVGERTREGNDLYHE B_Desulfovibrio_vulgaris

81832140 VDLLAPYARGGKIGLFGGAGVGKTVLIMELINNIAKQHGGFSVFAGVGERTREGNDLWM B_Geobacter_sulfurreducens

22326673 DLLAPYQRGGKIGLFGGAGVGKTVLIMELINNVAKAHGGFSVFAGVGERTREGNDLYRE E_Arabidopsis_thaliana_M

114543 DLLAPYAKGGKIGLFGGAGVGKTVLIMELINNVAKAHGGYSVFAGVGERTREGNDLYHE E_Bos_taurus_M

47606749 DLLAPYAKGGKIGLFGGAGVGKTVLIMELINNVAKAHGGYSVFAGVGERTREGNDLYNE E_Drosophila_melanogaster_M

83592562 DLIAPYTKGGKVGLFGGAGVGKTVLIQELINNVAKGHGGYSVFAGVGERTREGNDLYHE B_Rhodospirillum_rubrum

2493025 DLLAPYSKGGKIGLFGGAGVGKTVLIMELINNIAKVHSGFSVFAGVGERTREGNDLYHE B_Paracoccus_denitrificans

114570 DLLTPYRQGGKIGLFGGAGVGKTVIMMELINNIAIQHGGVSVFGGVGERTREGNDLYNE B_Synechocystis_PCC_6803

6686269 DLLAPYRRGGKIGLFGGAGVGKTVLIMELINNIAKAHGGVSVFGGVGERTREGNDLYME E_Arabidopsis thaliana_C

11465725 DLLAPYRRGGKIGLFGGAGVGKTVLIMELINNIAKAHGGVSVFGGVGERTREGNDLYME E_Porphyra_purpurea_C

1703680 DLICPYVRGGKIGLFGGAGVGKTVLIQELINNIATQHGGLSVFAGVGERTREGNDLYYE B_Acetobacterium_woodii

15673746 DLLAPYLKGGKVGLFGGAGVGKTVLIQELIHNIAQEHGGISVFTGVGERTREGNDLYWE B_Lactococcus_lactis

29377093 DLLAPYLKGGKVGLFGGAGVGKTVLIQELIHNIAQEHGGISVFTGVGERTREGNDLYYE B_Enterococcus_faecalis_F

1168575 DLLAPYLKGGKVGLFGGAGVGKTVLIQELIHNIAQEHGGISVFTGVGERTREGNDLYYE B_Enterococcus_hirae_F

114571 DLLAPYIKGGKIGLFGGAGVGKTVLIQELIHNIAQEHGGISVFAGVGERTREGNDLYHE B_Bacillus_PS3

14916961 DLLAPYIKGGKIGLFGGAGVGKTVLIQELINNIAQEHGGISVFAGVGERTREGNDLYHE B_Bacillus_pseudofirmus

20137589 DLLAPYQKGGKIGLFGGAGVGKTVLIQELINNIAKEHGGLSVFTGVGERTREGNDLYYE B_Clostridium_acetobutylicum

22266799 DLLAPYVKGGKIGLFGGAGVGKTVLIMELINNIAKGHGGLSVFAGVGERTREGRDLFDE B_Ilyobacter_tartaricus

1352033 DLLAPYVKGGKIGLFGGAGVGKTVLIMELINNIAQGHGGLSVFAGVGERTREGRDLYDE B_Propionigenium_modestum

77964181 DLIAPYAKGGKIGLFGGAGVGKTVLIMELINNIAKEHGGLSVFSGVGERTREGNDLYHE B_Clostridium_paradoxum

2662066 DLIAPYLKGGKIGLFGGAGVGKTVLIQELINNVANQHGGISVFTGVGERTREGNDLYWE B_Ruminococcus_albus

137464 DALFPCVQGGTTCIPGAFGCGKTVISQSLSK---YSNSDAIIYVGCGERGNEMAEVLME E_Saccharomyces_cerevisiae_VA

92090652 DSLFPCVQGGTCAIPGAFGCGKTVISQSLSK---FSNSDAIVYVGCGERGNEMAEVLME E_Dictyostelium_discoideum_VA

3334404 DALFPSVLGGTCAIPGAFGCGKTVISQALSK---YSNSDAVVYVGCGERGNEMAEVLMD E_Arabidopsis_thaliana_VA

124053334 DALFPCVQGGTTAIPGAFGCGKTVISQSLSK---YSNSDVIIYVGCGERGNEMSEVLRD E_Bos_taurus_VA

93141332 DSLFPCVQGGTTAIPGAFGCGKTVISQALSK---YSNSDVIIYVGCGERGNEMSEVLRD E_Drosophila_melanogaster_VA

25009559 DTMFPIAKGGTAAVPGPFGSGKTVMIRTLSM---FAQSRFIIPVLCGERGNEAADALQG A_Pyrobaculum_aerophilum_VA

126352990 DTVFPIAKGGAASIPGPFGSGKTVTIRSLML---YAMTQYSVPVLCGERGNEAADALQG A_Caldivirga maquilingensis_VA

41614899 DIMFPIAKGGSAAVPGPFGSGKTVLNQQIAK---WADSDIVIYIGCGERGNEMTEVLEE A_Nanoarchaeum_equitans_VA

126008291 DSFFPVAKGGTVAVPGPFGSGKTVIQHQLSK---WSDADITVYVGCGERGNEMTEILST A_Ferroplasma_acidarmanus_VA

48477562 DAFFPVAKGGTVAVPGPFGSGKTVIQHQLSK---WSDSDIVVYVGCGERGNEMTEILST A_Picrophilus_torridus_VA

12229704 DGLFPIAKGGTAAIPGPFGSGKTVTQQSLAK---FADADIVVYIGCGERGNEMTEVIED A_Halobacterium salinarium_VA

2493099 DGLFPIAKGGTAAIPGPFGSGKTVTQHQLAK---WADADIVVYVGCGERGNEMTEVIED A_Haloferax_volcanii

12585484 DVMFPLVMGGAAAIPGPFGSGKTVTQQSVAK---YGNADIVVYVGCGERGNEMTDVLVE B_Deinococcus_radiodurans_VA

32172455 DVLFPVAMGGTAAIPGPFGSGKTVTQQSLAK---WSNADVVVYVGCGERGNEMTDVLVE B_Thermus_thermophilus_VA

116754898 DGFFPLAKGGTAAIPGGFGTGKTVMQQTLSK---WSDVDIVIYVGCGERGNEMADLLHE A_Methanosaeta_thermophila_VA

12585404 DTFFPVAKGGTAAIPGPFGSGKTVTQHQLAK---WSDAQIVVYIGCGERGNEMTEVLEE A_Archaeoglobus_fulgidus_VA

91773223 DGMFPIAKGGTAAIPGPFGSGKTVTQQQLAK---WSDTDIVVYIGCGERGNEMADVLNE A_Methanococcoides_burtonii_VA

22002078 DGLFPVAKGGTAAIPGPFGSGKTVTQQQLSK---WSDTEIVVYIGCGERGNEMADVLWE B_Methanosarcina_mazei_VA

114520 DGLFPVAKGGTAAIPGPFGSGKTVTQQSLAK---WSDTEIVVYIGCGERGNEMADVLSE B_Methanosarcina_barkeri_VA

29376065 DTFFPITKGGAAAVPGPFGAGKTVVQHQIAK---WADVDLVVYVGCGERGNEMTDVLNE B_Enterococcus_faecalis_VA

1171780 DTFFPVTKGGAAAVPGPFGAGKTVVQHQIAK---WSDVDLVVYVGCGERGNEMTDVVNE B_Enterococcus hirae_VA

76795559 DTLFPVTKGGTACIPGPFGSGKTVVQHQLAK---WADAEIVVYIGCGERGNEMTDVLLE B_Thermoanaerobacter_ethanolicus_VA

110798759 DTFFPVAKGGAAAVPGPFGAGKTVVQHQVAK---WGDTEIVVYVGCGERGNEMTDVLNE B_Clostridium_perfringens_VA

87082847 DTFFPVAKGGTACVPGPFGSGKTVVQHQLAK---WADAEVVVYIGCGERGNEMTDVLME B_Caloramator fervidus_VA

12585422 DTFFPQAKGGTAAIPGPFGSGKTVTQHQLAK---WSDAQVVIYIGCGERGNEMTDVLEE A_Pyrococcus_horikoshii_VA

57641537 DTFFSQAKGGTAAIPGPFGSGKTVTQHQLAK---WSDAQVVVYIGCGERGNEMTDVLEE A_Thermococcus_kodakarensis_VA

12585563 DTFFTLAKGGTAAIPGPFGSGKTVTQHQLAK---WSDADVVVYIGCGERGNEMTEVIEE A_Methanocaldococcus_jannaschii_VA

45358607 DTFFGLAKGGASAIPGPFGSGKTVTQHQLAK---WSDVDVVVYIGCGERGNEMTEVIEE A_Methanococcus_maripaludis_VA

84489927 DTFFCVAKGGTSAMPGPFGSGKTVTQQQLAK---WADADIVVYIGCGERGNEMTEVLTE A_Methanosphaera_stadtmanae_VA

12585397 DTFFSVAKGGTAAIPGPFGSGKTVTQQQLAK---WADADIIVYVGCGERGNEMTEVLKE A_M_thermautotrophicus_VA

118195150 DTFFPIAKGGTGSIPGGFGTGKTVTLHQIAK---WADSQVVVYIGCGERGNEMTEVLVE A_Cenarchaeum_symbiosum_VA

12585522 DTFFPMAKGGTGAVPGGFGTGKTVTLHSLAQ---WSEARVVIYIGCGERGNEMTEVLER A_Aeropyrum_pernix_VA

74053565 DTIFPIAKGGTAAIPGPFGSGKTVTLQSLAK---WSEAKVVIYVGCGERGNEMTDELRQ A_Sulfolobus_acidocaldarius_VA

53714024 DTVNPIVEGGTGFIPGPFGTGKTVLQHAISK---QAEADIVIIAACGERANEVVEIFTE B_Bacteroides_fragilis_VA

12585442 DTQIPVLKGGTFCTPGPFGAGKTVLQHHLSK---YAAVDIVVLCACGERAGEVVEILQE B_Chlamydia_trachomatis_VA

12585416 DTFFPVAKGGTFCIPGPFGAGKTVLQQVTSR---NADVDVVIIAACGERAGEVVETLKE B_Borrelia_burgdorferi_VA

12585431 DTFFPVAKGGTYCIPGPFGAGKTVLQHSTSR---NADVDVVVIAACGERAGEVVETLRE B_Treponema_pallidum_VA

**Continued on the next page.**

**GI number Alignment**  **Species names as in the Figure 5**

2605819 MKDNTVMVFGQMNEPPGARFRVGHTALTMAEYFRDRRDVLLLIDNIFRFIQAGSEVSGL B_Methanosarcina_barkeri_F

20091272 MKENTVMVFGQMNEPPGARFRVGHVALTMAEYFRDHRDVLLLIDNIFRFIQAGSEVSGL B_Methanosarcina_acetivorans_F

61219617 MSEKTALVFGQMDEPPGTRLRVALAGLTMAEYFRDKQDVLFFIDNIFRFTQAGSEVSTL B_Streptomyces_coelicolor

15608450 LAEDTALVFGQMDEPPGTRMRVALSALTMAEWFRDGQDVLLFIDNIFRFTQAGSEVSTL B_Mycobacterium_tuberculosis

3913128 MKEYTVMVYGQMNEPPGVRFRVAHTGLTMAEYFRDGQDVLIFIDNIFRFVQAGAEVSTL B_Aquifex_aeolicus

2493023 MKEKVALCYGQMNEPPGARNRIAFTGLTMAEYFRDGLDVLMFIDNIFRYAQSGAEMSAL B_Helicobacter_pylori

81467688 MKEKTVLCYGQMNEPPGARLRVALSALTMAEHFRDGTDVLLFVDNIFRFSQAGSEVSAL B_Leptospira_interrogans

3913124 MQENTVLVFGQMNEPPGARFRVALTALTIAEYFRDGRDVLLFIDNIFRFVQAGSEVSAL B_Thermotoga_maritima

66826095 MVEKVALVFGQMNEPPGARARVTLTGLTVAEYFRDGQDVLLFIDNIFRFTQAGSEMSAL E_Dictyostelium_discoideum_M

53713460 MIEQATLVFGQMNEPPGARASVALSGLTVAESFRDARDILFFIDNIFRFTQAGSEVSAL B_Bacteroides_fragilis_F

114573 MQEKVAMVYGQMNEPPGNRLRVALTGLTMAERFRDGRDVLLFIDNIYRYTLAGTEVSAL B_Vibrio alginolyticus

14547933 MQEKVAMVYGQMNEPPGNRLRVALTGLTMAEKFRDGRDVLLFIDNIYRYTLAGTEVSAL B_Vibrio cholerae

15677764 MKDKVAMVYGQMNEPPGNRLRVALTGLTMAEYFRDGRDVLFFVDNIYRYTLAGTEVSAL B_Neisseria_meningitidis

16131600 MTDKVSLVYGQMNEPPGNRLRVALTGLTMAEKFRDGRDVLLFVDNIYRYTLAGTEVSAL B_Escherichia_coli

25452812 MMEKTALVFGQMNEPPGARARVALTGLSIAEYFREGRDVLLFIDNIFRFTQAGSEVSAL B_Chlorobium_tepidum

81830486 MKDKAALIYGQMNEPPGARARVALTALACAEYFRDNQDVLLFVDNIFRFTQAGSEVSAL B_Desulfovibrio_vulgaris

81832140 EMDKAALVYGQMNEPPGARARVALSALSIAEYFRDEQNVLLFVDNIFRFTQAGSEVSAL B_Geobacter_sulfurreducens

22326673 MIEKCALVYGQMNEPPGARARVGLTGLTVAEYFRDGQDVLLFIDNIFRFTQANSEVSAL E_Arabidopsis_thaliana_M

114543 MIEKVALVYGQMNEPPGARARVALTGLTVAEYFRDGQDVLLFIDNIFRFTQAGSEVSAL E_Bos_taurus_M

47606749 MIEKVALVYGQMNEPPGARARVALTGLTVAEYFRDGQDVLLFIDNIFRFTQAGSEVSAL E_Drosophila_melanogaster_M

83592562 MIDKVALVYGQMNEPPGARARVALAGLTQAEYFRDGQDVLFFVDNIFRFTQAGSEVSAL B_Rhodospirillum_rubrum

2493025 MIEKVALVYGQMNEPPGARARVALTGLTLAEQFRDGTDVLFFVDNIFRFTQAGSEVSAL B_Paracoccus_denitrificans

114570 MIEKIALVYGQMNEPPGARMRVGLTALTMAEYFRDKQDVLLFIDNIFRFVQAGSEVSAL B_Synechocystis_PCC_6803

6686269 MKEKVALVYGQMNEPPGARMRVGLTALTMAEYFRDEQDVLLFIDNIFRFVQAGSEVSAL E_Arabidopsis thaliana_C

11465725 MKEKVALVYGQMNEPPGARMRVGLTALTMAEYFRDKQDVLLFIDNIFRFVQAGSEVSAL E_Porphyra_purpurea_C

1703680 MMEKTALCFGQMNEPPGARMRIALAGLTMAEYFRDGQDVLLFIDNIFRFTQAGSEVSAL B_Acetobacterium_woodii

15673746 MKEKTAMVFGQMNEPPGARMRVALTGLTIAEYFRDGQDVLLFIDNIFRFTQAGSEVSAL B_Lactococcus_lactis

29377093 MKDKTAMVFGQMNEPPGARMRVALTGLTIAEYFRDGQDVLLFIDNIFRFTQAGSEVSAL B_Enterococcus_faecalis_F

1168575 MKDKTAMVFGQMNEPPGARMRVALTGLTIAEYFRDGQDVLLFIDNIFRFTQAGSEVSAL B_Enterococcus_hirae_F

114571 MKDKTAMVFGQMNEPPGARMRVALTGLTMAEYFRDGQDGLLFIDNIFRFTQAGSEVSAL B_Bacillus_PS3

14916961 MTDKTAMVFGQMNEPPGARMRVALSGLTMAEHFRDGQDVLLFVDNIFRFTQAGSEVSAL B_Bacillus_pseudofirmus

20137589 MKDKTALVFGQMNEPPGARMRVALTGLTMAEYFRDGQDVLLFIDNIFRFTQAGSEVSAL B_Clostridium_acetobutylicum

22266799 MTEKTSLVYGQMNEPPGARLRVALTGLTMAENFRDGQDVLLFVDNIFRFTQAGSEVSAL B_Ilyobacter_tartaricus

1352033 MLEKTSLVYGQMNEPPGARLRVGLTGLTMAENFRDGQDVLFFVDNIFRFTQAPSEVSAL B_Propionigenium_modestum

77964181 MIDKTTLVYGQMNEPPGARMRVALTGLTMAEYFRDGQDVLLFIDNIFRFTQAGSEVSAL B_Clostridium_paradoxum

2662066 MKEKTVLVYGQMNEPPGARMRVGLSGLTMAEYFRDGQDVLLFIDNIFRFTQAGSEVSAL B_Ruminococcus_albus

137464 FPERTTLVANTSNMPVAAREASIYTGITLAEYFRDGKNVSMIADSSSRWAEALREISGR E_Saccharomyces_cerevisiae_VA

92090652 FPERTCLVANTSNMPVAAREASIYTGITLAEYFRDGLNVAMMADSTSRWAEALREISGR E_Dictyostelium_discoideum_VA

3334404 FPQRTTLVANTSNMPVAAREASIYTGITIAEYFRDGYNVSMMADSTSRWAEALREISGR E_Arabidopsis_thaliana_VA

124053334 FPERTALVANTSNMPVAAREASIYTGITLSEYFRDGYHVSMMADSTSRWAEALREISGR E_Bos_taurus_VA

93141332 FPERTALVANTSNMPVAAREASIYTGITLSEYFRDGYNVSMMADSTSRWAEALREISGR E_Drosophila_melanogaster_VA

25009559 LLKRTTIIVNTSNMPVAAREASVYMGTTLGEYFRDGYDVLVLADSTSRWAEAMREVALR A_Pyrobaculum_aerophilum_VA

126352990 LLKRETIIVNTSNMPVAAREASIYMGATIAEYFRDGYDVLLMADSTSRWAEAMREVALR A_Caldivirga maquilingensis_VA

41614899 FPKRTILIANTSNMPIAAREASIYLGATIGEYFRDGYSVVVNADSTSRWAEALREISSR A_Nanoarchaeum_equitans_VA

126008291 FPEKTILIANTSNMPVAAREASIYTGVTIAEYYRDGYDVALMADSTSRWAEALREISGR A_Ferroplasma_acidarmanus_VA

48477562 FPERTVLIANTSNMPVAAREASIYTGVTIAEYYRDGYNVALMADSTSRWAEALREISGR A_Picrophilus_torridus_VA

12229704 FPERTTLIANTSNMPVAARESCIYTGITIAEYYRDGYDVALMADSTSRWAEAMREISSR A_Halobacterium salinarium_VA

2493099 FPERTCLIANTSNMPVAARESCIYTGITIAEYYRDGYDVALMADSTSRWAEAMREISSR A_Haloferax_volcanii

12585484 FPERTILIANTSNMPVAAREASVYTGVTLAEYFRDGYSVSLMADSTSRWAEALREISSR B_Deinococcus_radiodurans_VA

32172455 FPERTVLIANTSNMPVAAREASIYVGVTIAEYFRDGFSVALMADSTSRWAEALREISSR B_Thermus_thermophilus_VA

116754898 FPERSIVYANTSNMPVAAREASIYTGMTTAEYYRDGYDVLMTADSTSRWAEAMRELASR A_Methanosaeta_thermophila_VA

12585404 FPERTVLVANTSNMPVAAREASVYTGITIAEYFRDGYDVAIQADSTSRWAEAMREISGR A_Archaeoglobus_fulgidus_VA

91773223 FPERTVLIANTSNMPVAAREASVYTGITIAEYYRDGYDVSLMADSSSRWAEAMREISSR A_Methanococcoides_burtonii_VA

22002078 FPERTILVANTSNMPVAAREASVYTGMTLAEYFRDGYDVSLMADSTSRWAEAMREISSR B_Methanosarcina_mazei_VA

114520 FPERTVLIANTSNMPVAAREASVYTGITIAEYYRDGLDVSLMADSTSRWAEAMREISSR B_Methanosarcina_barkeri_VA

29376065 FPERTILIANTSNMPVAAREASIYTGITIAEYFRDGYSVAIMADSTSRWAEALREMSGR B_Enterococcus_faecalis_VA

1171780 FPERTVLIANTSNMPVAAREASIYTGITIAEYFRDGYDVAIMADSTSRWAEALREMSGR B_Enterococcus hirae_VA

76795559 FPERTVLIANTSNMPVAAREASIYTGITIAEYFRDGYSVALMADSTSRWAEALREMSGR B_Thermoanaerobacter_ethanolicus_VA

110798759 FPERTVLIANTSNMPVAAREASIYTGITIAEYFRDGYSVSIMADSTSRWAEALREMSGR B_Clostridium_perfringens_VA

87082847 FPERTVLIANTSNMPVAAREASIYTGITIAEYFRDGYSVAMMADSTSRWAEALREMSGR B_Caloramator fervidus_VA

12585422 FPKRTVLIANTSNMPVAAREASIYTGITIAEYFRDGYDVALMADSTSRWAEALREISGR A_Pyrococcus_horikoshii_VA

57641537 FPKRTVLIANTSNMPVAAREASIYTGITIAEYFRDGYDVALMADSTSRWAEALREISGR A_Thermococcus_kodakarensis_VA

12585563 FPHRTVLIANTSNMPVAAREASVYTGITIAEYFRDGYGVLLTADSTSRWAEAMREISGR A_Methanocaldococcus_jannaschii_VA

45358607 FPHRTVLIANTSNMPVAAREASVYTGITIAEYFRDGLGVLLTADSTSRWAEAMREISGR A_Methanococcus_maripaludis_VA

84489927 FPERTVLIANTSNMPVAAREACVYTGITIAEYFRDGYDVALMADSTSRWAEAMRELSGR A_Methanosphaera_stadtmanae_VA

12585397 FPERTVLIANTSNMPVAAREACVYTGITIAEYFRDGYDVALMADSTSRWAEAMREISGR A_M_thermautotrophicus_VA

118195150 FPHRTVLVANTSNMPVAAREASIYTGVTIAEYYRDGKDVVLVADSTSRWAEALREMSGR A_Cenarchaeum_symbiosum_VA

12585522 FPQRTVLIANTSNMPVAAREASIYVGITIAEYYRDGYDVLLVADSTSRWAEALREIAGR A_Aeropyrum_pernix_VA

74053565 FPKRTILVANTSNMPVAARESSIYVGVTMAEYFRDGYDVLLVADSTSRWAEALRELGGR A_Sulfolobus_acidocaldarius_VA

53714024 FPERTIIIANTSNMPVAAREASVYTAMTIAEYYRAGLKVLLMADSTSRWAQALREMSNR B_Bacteroides_fragilis_VA

12585442 FPHRTCIICNTSSMPVAARESSIYLGITIAEYYRQGLHILLLADSTSRWAQALREISGR B_Chlamydia_trachomatis_VA

12585416 FPERTCIICNTSSMPVAAREASVYTAITIGEYYRQGLDILLLADSTSRWAQAMREMSGR B_Borrelia_burgdorferi_VA

12585431 FPDRTVIVCNTSSMPVASREASVYTGVTLAEYYRQGLDVLLLADSTSRWAQALREMSGR B_Treponema_pallidum_VA

**Continued on the next page.**

**GI number Alignment**  **Species names as in the Figure 5**

2605819 MGQMPSRLGYQPTLGTELSELEERISTTDAGAIMSIQAVYVPADDFTDPSAVHTFSHLS B_Methanosarcina_barkeri_F

20091272 MGQMPSRLGYQPTMGTELSELEERISNTDAGAIMSIQAVYVPADDFTDPAAVHTFSHLS B_Methanosarcina_acetivorans_F

61219617 LGRMPSAVGYQPNLADEMGLLQERITSTRGHSITSMQAIYVPADDLTDPAPATTFAHLD B_Streptomyces_coelicolor

15608450 LGRMPSAVGYQPTLADEMGELQERITSTRGRSITSMQAVYVPADDYTDPAPATTFAHLD B_Mycobacterium_tuberculosis

3913128 LGRLPSAVGYQPTLNTDVGEVQERITSTKKGSITAIQAVYVPADDITDPAPWSIFAHLD B_Aquifex_aeolicus

2493023 LGRIPSAVGYQPTLAGEMGKLQERIASTKNGSITSVQAVYVPADDLTDPAPASVFAHLD B_Helicobacter_pylori

81467688 LGRMPSAVGYQPTLSTEMGALQERITSTKKGSITSVQAIYVPADDLTDPAPANAFAHLD B_Leptospira_interrogans

3913124 LGRMPSAVGYQPTLATDMGELQERITSTRRGSITSVQAIYVPADDITDPAPATTFAHLD B_Thermotoga_maritima

66826095 LGRIPSAVGYQPTLATDMGCMQERIATTKKGSITSVQAVYVPADDLTDPAPATTFAHLD E_Dictyostelium_discoideum_M

53713460 LGRMPSAVGYQPTLATEMGAMQERITSTKTGSITSVQAVYVPADDLTDPAPATTFTHLD B_Bacteroides_fragilis_F

114573 LGRMPSAVGYQPTLAEEMGVLQERITSTKSGSITSVQAVYVPADDLTDPSPATTFAHLD B_Vibrio alginolyticus

14547933 LGRMPSAVGYQPTLAEEMGVLQERITSTKKGSITSVQAVYVPADDLTDPSPATTFAHLD B_Vibrio cholerae

15677764 LGRMPSAVGYQPTLAEEMGRLQERITSTQTGSITSIQAVYVPADDLTDPSPATTFAHLD B_Neisseria_meningitidis

16131600 LGRMPSAVGYQPTLAEEMGVLQERITSTKTGSITSVQAVYVPADDLTDPSPATTFAHLD B_Escherichia_coli

25452812 LGRMPSAVGYQPTLSTEMGELQDRITSTKKGSVTSVQAIYVPADDLTDPAPATAFTHLD B_Chlorobium_tepidum

81830486 LGRMPSAVGYQPTLGTDLGALQERITSTTKGSITSVQAVYVPADDLTDPAPATTFSHLD B_Desulfovibrio_vulgaris

81832140 LGRIPSAVGYQPTLATEMGELQERITSTTKGSITSVQAIYVPADDLTDPAPATAFAHLD B_Geobacter_sulfurreducens

22326673 LGRIPSAVGYQPTLASDLGALQERITTTKKGSITSVQAIYVPADDLTDPAPATTFAHLD E_Arabidopsis_thaliana_M

114543 LGRIPSAVGYQPTLATDMGTMQERITTTKKGSITSVQAIYVPADDLTDPAPATTFAHLD E_Bos_taurus_M

47606749 LGRIPSAVGYQPTLATDMGSMQERITTTKKGSITSVQAIYVPADDLTDPAPATTFAHLD E_Drosophila_melanogaster_M

83592562 LGRIPSAVGYQPTLATDMGALQERITSTKKGSITSVQAIYVPADDLTDPAPAASFAHLD B_Rhodospirillum_rubrum

2493025 LGRIPSAVGYQPTLATDMGALQERITSTKAGSITSVQAIYVPADDLTDPAPATSFAHLD B_Paracoccus_denitrificans

114570 LGRMPSAVGYQPTLGTDVGDLQERITSTKEGSITSIQAVYVPADDLTDPAPATTFAHLD B_Synechocystis_PCC_6803

6686269 LGRMPSAVGYQPTLSTEMGTLQERITSTKKGSITSIQAVYVPADDLTDPAPATTFAHLD E_Arabidopsis thaliana_C

11465725 LGRMPSAVGYQPTLATEMGALQERITSTTEGSITSIQAVYVPADDLTDPAPATTFAHLD E_Porphyra_purpurea_C

1703680 LGRMPSAVGYQPTLATEMGALQERITSTSKGSITSVQAVYDPADDLTDPAPATTFAHLD B_Acetobacterium_woodii

15673746 LGRMPSAVGYQPTLATEMGQLQERITSTKKGSVTSIQAIYVPADDYTDPAPATAFAHLD B_Lactococcus_lactis

29377093 LGRMPSAVGYQPTLATEMGQLQERITSTKKGSITSIQAIYVPADDYTDPAPATAFAHLD B_Enterococcus_faecalis_F

1168575 LGRMPSAVGYQPTLATEMGQLQERITSTKKGSITSIQAIYVPADDYTDPAPATAFAHLD B_Enterococcus_hirae_F

114571 LGRMPSAIGYQPTLATEMGQLQERITSTAKGSITSIQAIYVPADDYTDPAPATTFSHLD B_Bacillus_PS3

14916961 LGRMPSAVGYQPTLATEMGQLQERITSTKVGSVTSIQAIYVPADDYTDPAPATTFAHLD B_Bacillus_pseudofirmus

20137589 LGRIPSAVGYQPTLANEMGALQERITSTKQGSITSVQAVYVPADDLTDPAPATTFTHLD B_Clostridium_acetobutylicum

22266799 LGRMPSAVGYQPTLATDMGALQERITSTKTGSITSVQAVYVPADDLTDPAPATTFTHLD B_Ilyobacter_tartaricus

1352033 LGRMPSAVGYQPNLATDMGALQERITSTKTGSITSVQAVYVPADDLTDPAPATTFTHLD B_Propionigenium_modestum

77964181 LGRMPSAVGYQPTLATEMGALQERITSTKKGSITSVQAVYVPADDLTDPAPATTFAHLD B_Clostridium_paradoxum

2662066 LGRMPSAVGYQPTLATEMGALQERITSTNKGSITSVQAVYVPADDLTDPAPATTFAHLD B_Ruminococcus_albus

137464 LGEMPADQGFPAYLGAKLASFYERAGKDRTGSVSIVAAVSPAGGDFSDPVTTATLGITQ E_Saccharomyces_cerevisiae_VA

92090652 LAEMPADSGYPAYLGARLASFYERAGRTRIGSVTIVGAVSPPGGDFADPVTAATLGIVQ E_Dictyostelium_discoideum_VA

3334404 LAEMPADSGYPAYLAARLASFYERAGKERNGSVTIVGAVSPPGGDFSDPVTSATLSIVQ E_Arabidopsis_thaliana_VA

124053334 LAEMPADSGYPAYLGARLASFYERAGREREGSVSIVGAVSPPGGDFSDPVTSATLGIVQ E_Bos_taurus_VA

93141332 LAEMPADSGYPAYLGARLASFYERAGREREGSVSIVGAVSPPGGDFSDPVTSATLGIVQ E_Drosophila_melanogaster_VA

25009559 IGEMPSEEGYPAYLPTRLAEFYERAGRERVGSLTIAASVSPPGGDFTEPVTSNTLRFIG A_Pyrobaculum_aerophilum_VA

126352990 IGEMPSEEGFPAYLPTRLAEFYERAGKGGLGSLTIAASVSPPGGDFTEPVTSHTLRFIG A_Caldivirga maquilingensis_VA

41614899 LGEIPSEEGYPAYLLRKLAEFYERSGRDLEGSLTIIGAVSPPGGDFSEPVTQNTLRLVG A_Nanoarchaeum_equitans_VA

126008291 LEEMPGEEGYPAYLGRRISEFYERSGNERTGSITLIGAVSPPGGDLSDPVVQNTLRVTR A_Ferroplasma_acidarmanus_VA

48477562 LEEMPGEEGYPAYLGRRISEFYERSGNQRTGSVTLIGAVSPPGGDLSDPVVQNTLRVTR A_Picrophilus_torridus_VA

12229704 LEEMPGEEGYPAYLAARLSEFYERAGYGTEGSISVIGAVSPPGGDFSEPVTQNTLRIVK A_Halobacterium salinarium_VA

2493099 LEEMPGEEGYPAYLAARLSEFYERAGYGEEGSVSVIGAVSPPGGDFSEPVTQNTLRIVK A_Haloferax_volcanii

12585484 LEEMPAEEGYPPYLGAKLAAFYERAGAGEDGAVSVIGAVSPAGGDMSEPVTQATLRITG B_Deinococcus_radiodurans_VA

32172455 LEEMPAEEGYPPYLAARLAAFYERAGKGEEGAVTIVGAVSPPGGDMSEPVTQSTLRIVG B_Thermus_thermophilus_VA

116754898 LEEMPGEEGYPAYLAARLADFYERAGRGGEGSVAVVGAVSPPGGDFTEPVTQNTLRIVK A_Methanosaeta_thermophila_VA

12585404 LEEMPGEEGYPAYLASRLAEFYERAGRGNIGSVTVVGAVSPPGGDFSEPVTQNTLRIVK A_Archaeoglobus_fulgidus_VA

91773223 LEEMPGEEGYPAYLSARLSEFYERAGAGLDGSITVIGAVSPPGGDFSEPVTQNTLRIVK A_Methanococcoides_burtonii_VA

22002078 LEEMPGEEGYPAYLSARLAEFYERAGVGEKGSITAIGAVSPPGGDFSEPVTQNTLRIVK B_Methanosarcina_mazei_VA

114520 LEEMPGEEGYPAYLSARLAEFYERAGVGETGSITVIGAVSPPGGDFSEPVTQNTLRIVK B_Methanosarcina_barkeri_VA

29376065 LEEMPGDEGYPAYLGSRLAEYYERAGQHREGSITAISAVSPSGGDISEPVTQNTLRVVK B_Enterococcus_faecalis_VA

1171780 LEEMPGDEGYPAYLGSRLAEYYERSGRQREGSITAISAVSPSGGDISEPVTQNTLRVVK B_Enterococcus hirae_VA

76795559 LEEMPGEEGYPAYLARRLAEFYERAGRNREGALTVVGAVSPPGGDLSEPVTQATLRVVK B_Thermoanaerobacter_ethanolicus_VA

110798759 LEEMPGDEGYPAYLGSRLADYYERAGKGREGAVTAIGAVSPPGGDISEPVTQSTLRIVK B_Clostridium_perfringens_VA

87082847 LEEMPGEEGYPAYLASRAAEFYERAGKNRIGALTVIGAVSPPGGDLSEPVTQATLKIVK B_Caloramator fervidus_VA

12585422 LEEMPGEEGYPAYLASKLAEFYERAGRYRVGSVSVIGAVSPPGGDFSEPVVQNTLRVVK A_Pyrococcus_horikoshii_VA

57641537 LEEMPGEEGYPAYLASKIAEFYERAGRERVGSVSVIGAVSPPGGDFSEPVVQNTLRVVK A_Thermococcus_kodakarensis_VA

12585563 LEEMPGEEGYPAYLASRLAQFYERAGRNRQGFVCIVGAVSPPGGDFSEPVTSNTLRIVK A_Methanocaldococcus_jannaschii_VA

45358607 LEEMPGEEGYPAYLSSKLAQFYERAGRNKQGFVCIVGAVSPPGGDFSEPVTSNTLRIVK A_Methanococcus_maripaludis_VA

84489927 LEEMPGEEGYPAYLASRLAQFYERAGRKAEASVTVVGAVSPPGGDLSEPVTQNTLRIAK A_Methanosphaera_stadtmanae_VA

12585397 LEEMPGEEGYPAYLASRLAQFYERAGRDKIASVSVVGAVSPPGGDLSEPVTQNTLRICK A_M_thermautotrophicus_VA

118195150 LEEMPAEEGYPSYLASRLAEFYERAGRERNGSVTLVGAVSPSGGDFTEPVTTHTMRFIK A_Cenarchaeum_symbiosum_VA

12585522 LEEMPAEEGYPSYLASRLAEFYERAGRERSGSVTVVGAVSPPGGDFSEPVTSHTTRFIR A_Aeropyrum_pernix_VA

74053565 MEEMPAEEGFPSYLPSRLAEYYERAGRERFGSVSIASAVSPPGGDFTEPVTSNTLRFVR A_Sulfolobus_acidocaldarius_VA

53714024 MEELPGPDAFPMDLSSIISNFYGRAGYGESGSITFIGTVSPAGGNLKEPVTENTKKVAR B_Bacteroides_fragilis_VA

12585442 LEEIPGEEAFPAYLASRIAAFYERGGAGSEGSLTICGAVSPAGGNFEEPVTQATLSVVG B_Chlamydia_trachomatis_VA

12585416 LEEIPGEEAFPAYLESVIASFYERAGIGDIGSVTVGGSVSPAGGNFEEPVTQATLKVVG B_Borrelia_burgdorferi_VA

12585431 LEEIPGEEAFPAYLESCIAAFYERAGVGEKGSVTIGGTVSPAGGNFEEPVTQATLKVVG B_Treponema_pallidum_VA

**Continued on the next page.**

**GI number Alignment**  **Species names as in the Figure 5**

2605819 ASIVLSRKRASEGLYPAIDPLQSNSKMATPGIIRQTLAQYSELKDIISMLGLEQLSPED B_Methanosarcina_barkeri_F

20091272 ASIVLSRKRASEGLYPAIDPLQSNSKMSTPGIIRQTLAQYAELKDIISMLGLEQLSQED B_Methanosarcina_acetivorans_F

61219617 ATTVLSRPISEKGIYPAVDPLDSTSRILDPRYVKNILQKYKDLQDIIAILGIDELGEED B_Streptomyces_coelicolor

15608450 ATTELSRAVFSKGIFPAVDPLASSSTILDPSVVIRILQRYKDLQDIIAILGIDELSEED B_Mycobacterium_tuberculosis

3913128 ATTVLTRRLAELGIYPAIDPLESTSKYLAPEYVKRILQRYKELQEIIAILGMEELSDED B_Aquifex_aeolicus

2493023 ATTVLNRKIAEKGIYPAVDPLDSTSRILSPQMIQQVLQKYKDLQDIIAILGLDELSEED B_Helicobacter_pylori

81467688 ATTVLSRAISDKGIYPAVDPLDSTSRVMNAQVVQRILQRYKDLQDIIAILGMDELSEDD B_Leptospira_interrogans

3913124 ATVVLSRRIAELGLYPAVDPLDSSSKILDPAIVQEVLQRYKDLQDIIAILGVEELSPED B_Thermotoga_maritima

66826095 ATTVLSRAISELGIYPCVDPLDSTSLMMDPNIVQKILQEYKSLQDIIAILGMDDLSEDQ E_Dictyostelium_discoideum_M

53713460 ATTVLSRKITELGIYPAVDPLESTSRILDPHIVKQILQRNKELQDIISILGMEELSDAD B_Bacteroides_fragilis_F

114573 ATVVLNRNIAAMGLYPAIDPLDSTSRMLDPLVVQQTLQRYKELKDIIAILGMDELSEED B_Vibrio alginolyticus

14547933 ATVVLNRNIAAMGLYPAIDPLDSTSRQLDPLVVQATLQRYKELKDIIAILGMDELSEAD B_Vibrio cholerae

15677764 ATVVLSRDIASLGIYPAVDPLDSTSRQLDPMVVQSTLQKYKELRDIIAILGMDELSDED B_Neisseria_meningitidis

16131600 ATVVLSRQIASLGIYPAVDPLDSTSRQLDPLVVQSILQRYQELKDIIAILGMDELSEED B_Escherichia_coli

25452812 ATTVLSRQIAELGIYPAVDPLDSTSRILDPNIVKQILQRYKDLQDIIAILGMDELSDED B_Chlorobium_tepidum

81830486 GTLVLSRQIAELGIYPAVDPLDSTSRILDPNVVQQVLQKYKDLQDIIAILGMDELSDED B_Desulfovibrio_vulgaris

81832140 ATTVLSRQIAELGIYPAVDPLDSTSRILDPQVVQYVLQKYKDLQDIIAILGMDELSEED B_Geobacter_sulfurreducens

22326673 ATTVLSRQISELGIYPAVDPLDSTSRMLSPHIVQKVLQNYKNLQDIIAILGMDELSEDD E_Arabidopsis_thaliana_M

114543 ATTVLSRAIAELGIYPAVDPLDSTSRIMDPNIVQKILQDYKSLQDIIAILGMDELSEED E_Bos_taurus_M

47606749 ATTVLSRAIAELGIYPAVDPLDSTSRIMDPNIVQKILQDYKSLQDIIAILGMDELSEED E_Drosophila_melanogaster_M

83592562 ATTTLNRSIAELGIYPAVDPLDSTSRALDPLVVQRVLQTYKSLQDIIAILGMDELSEED B_Rhodospirillum_rubrum

2493025 ATTVLSRAISELGIYPAVDPLDSTSRILDPQVVQGMLQRYKSLQDIIAILGMDELSEED B_Paracoccus_denitrificans

114570 GTTVLSRGLAAKGIYPAVDPLDSTSTMLQPSIVQSTLQRYKELQDIIAILGLDELSEED B_Synechocystis_PCC_6803

6686269 ATTVLSRGLAAKGIYPAVDPLDSTSTMLQPRIVKQTLQRYKELQDIIAILGLDELSEED E_Arabidopsis thaliana_C

11465725 ATTVLSRNLAAKGIYPAVDPLDSTSTMLQPGIVKSTLQRYKELQDIIAILGLDELSEED E_Porphyra_purpurea_C

1703680 ATTVLSRAITEKGIYPAVDPLDSTSRILDPKIVQEILQRYKELQDIIAILGMDELSDAD B_Acetobacterium_woodii

15673746 ATTNLERRLTQMGIYPAVDPLASSSRALTPEIVQRVLQRYKELQDIIAILGMDELSDDE B_Lactococcus_lactis

29377093 ATTNLERKLTEQGIYPAVDPLASSSSALAPEIVQHILQRYRELQDIIAILGMDELSDDE B_Enterococcus_faecalis_F

1168575 ATTNLERKLTEQGIYPAVDPLASSSSALAPEIVQHVLQRYRELQDIIAILGMDELSDQE B_Enterococcus_hirae_F

114571 ATTNLERKLAEMGIYPAVDPLVSTSRALAPEIVQQTLERYKELQDIIAILGMDELSDED B_Bacillus_PS3

14916961 ATTNLERKLSEMGIYPAVDPLASTSRALSPEIVQQTLQKYKELQDIIAILGMDELSEED B_Bacillus_pseudofirmus

20137589 ATTVLSREISNLGIYPAVSPLESTSRILDPRIVKHILERYQELQDIIAILGVDELSDED B_Clostridium_acetobutylicum

22266799 ATTVLSRRIASLGIYPAVDPLDSTSTALQPGVVQSVLQRYKELQDIIAILGMDELSDED B_Ilyobacter_tartaricus

1352033 ATTVLSRRIASLGIYPAVDPLDSTSTALEPQIVQQILQRYKELQDIIAILGMDELSDED B_Propionigenium_modestum

77964181 ATTVLSRQIAALGIYPAVDPLDSTSRILDPNIVQSVLQRYKELQDIIAILGMDELSDED B_Clostridium_paradoxum

2662066 ATTVLSRSIASLGIFPAVDPLESTSRILTPEIVQSILQRYVELQDIIAIMGMDELSDED B_Ruminococcus_albus

137464 VFWGLDKKLAQRKHFPSINTSVSYSKYTNVLNMKEILSNAEELEQVVQLVGKSALSDSD E_Saccharomyces_cerevisiae_VA

92090652 VFWGLDKKLAQRKHFPSINWLISFSKYMQALDAKEILQMEEDLSEIVQLVGQDSLGESE E_Dictyostelium_discoideum_VA

3334404 VFWGLDKKLAQRKHFPSVNWLISYSKYSTALEAREVLQREDDLNEIVQLVGKDALAEGD E_Arabidopsis_thaliana_VA

124053334 VFWGLDKKLAQRKHFPSVNWLISYSKYMRALDAKEILQEEEDLAEIVQLVGKASLAETD E_Bos_taurus_VA

93141332 VFWGLDKKLAQRKHFPSINWLISYSKYMRALDVKEILQEEEDLSEIVQLVGKASLAETD E_Drosophila_melanogaster_VA

25009559 AFWPLSPRLAYSRHYPAIDWLVAFSRYVDTVELQSILVKEAELQEIVRILGTEALSEYE A_Pyrobaculum_aerophilum_VA

126352990 AFWPLDARLAYSRHYPAINWLQGFSRYVDSVAAIEVLTREAELSEIVRILGSEALSEQE A_Caldivirga maquilingensis_VA

41614899 ALWALDSKLAYKRHYPAINYLISYTKQWEFVKFFAILKRESELMDIVSIVGPDALSDNE A_Nanoarchaeum_equitans_VA

126008291 AFWALDASLASRRHFPSINWLNSYSLYLDSLSMMGILQKESELQEIVQLVGYDSLPENQ A_Ferroplasma_acidarmanus_VA

48477562 VFWALDASLASRRHFPSINWLTSYSLYTNNLSMMDLLEKESELQEIVQLVGYDALPEKE A_Picrophilus_torridus_VA

12229704 TFWALDSDLAERRHFPAINWDESYSLYKDQLDAVDILDEESELEEIVQLVGKDALPEDQ A_Halobacterium salinarium_VA

2493099 TFWALDADLAERRHFPAINWNESYSLYQEQLDAVDVLDEENELQEIVQLVGKDALPEDQ A_Haloferax_volcanii

12585484 AFWRLDAGLARRRHFPAINWNGSYSLFTPILDIGSLLQQEAALQEVVQLVGPDALQDNE B_Deinococcus_radiodurans_VA

32172455 AFWRLDASLAFRRHFPAINWNGSYSLFTSALDISELLQREAGLQEIVQLVGPDALQDAE B_Thermus_thermophilus_VA

116754898 VFWALDSRLTQRRHFPSINWLDSYSLYEKDLEAMAILQENAELEEIVMLVGSDALPEDQ A_Methanosaeta_thermophila_VA

12585404 VFWALDAKLAARRHFPAINWLQSYSLYVDTLKAMEVLQEEANLQEIVQLVGSDALPESQ A_Archaeoglobus_fulgidus_VA

91773223 VFWALDAKLSQRRHFPSINWLTSYSLYTQGLAAMDLLQQESELQEIVQLVGSDALPEDQ A_Methanococcoides_burtonii_VA

22002078 VFWALDAKLSQKRHFPAINWLNSYSLYKEDLNAMDMLQTESELQEIVQLVGSDALPEEQ B_Methanosarcina_mazei_VA

114520 VFWALDAKLSQRRHFPAINWLNSYSLYKDSLNAMEMLQTESELQEIVQLVGSDALPDDQ B_Methanosarcina_barkeri_VA

29376065 VFWGLDSQLAQKRHFPSINWLQSYSLYSTEVGGMRILQEESQLEEIVRLVGIDSLSDKD B_Enterococcus_faecalis_VA

1171780 VFWGLDSSLAQKRHFPSINWIQSYSLYSTEVGGMRILQEEEQLNEIVRLVGIDSLSDND B_Enterococcus hirae_VA

76795559 VFWALDSELAYARHFPAINWLTSYSLYSDVVEAMRLLQEEASLQEIVRLVGIDALSTRD B_Thermoanaerobacter_ethanolicus_VA

110798759 VFWGLDAQLAYKRHFPSINWLTSYSLYLEKMGAMALLQEEANLEEIVRLVGMDALSEGD B_Clostridium_perfringens_VA

87082847 VFWGLDANLAYRRHFPAINWLLSYSLYMEKIAAMKILQEENELQEIVRLVGIDALSEKD B_Caloramator fervidus_VA

12585422 VFWALDADLARRRHFPAINWLTSYSLYVDAVKAMALLQKESELQEIVRIVGPDALPERE A_Pyrococcus_horikoshii_VA

57641537 VFWALDADLARRRHFPAINWLRSYSLYIDAIQAMALLQKEAELQEIVRIVGPDALPDRE A_Thermococcus_kodakarensis_VA

12585563 VFWALDANLARRRHFPAINWLQSYSLYIDDVTAMSLLQKEAELQEIVQLVGPDALPDRE A_Methanocaldococcus_jannaschii_VA

45358607 VFWALDANLARRRHFPAINWLTSYSLYIDDIAAMSLLQKEAELQEIVQLVGPDALPDRE A_Methanococcus_maripaludis_VA

84489927 VFWALDASLADRRHFPSINWLNSYSLYVDSITAMALLQKESELNEIVQLVGPDALPQKD A_Methanosphaera_stadtmanae_VA

12585397 VFWALDASLADKRHFPSIDWLQSYSLYIDSVQAMALLQKEAELQEIVQLVGPDALPDRE A_M_thermautotrophicus_VA

118195150 TFWALDAKLAYSRHYPSINWMNSYSGYLADIAAYGILQREDTLKEIVRLLGPEALPDEE A_Cenarchaeum_symbiosum_VA

12585522 VFWALDTKLAYSRHYPAINWLMSYSAYVDLVTAMDILLRESELQEIVRLVGTEGLDEKD A_Aeropyrum_pernix_VA

74053565 VFWPLDVSLAQARHYPAINWIQGFSAYVDLVALVKILLREDELRQIVRLVGPESLSDKD A_Sulfolobus_acidocaldarius_VA

53714024 CFYALEQDRADKKRYPAVNPIDSYSKYIEYPEIKTRLQRGKEIAEQINILGDDGVPVEY B_Bacteroides_fragilis_VA

12585442 AFCGLSKARADARRYPSIDPMISWSKYLDSVAASRFLEEGAEIGKRIEVVGEEGISMED B_Chlamydia_trachomatis_VA

12585416 AFHGLTRERSDARKFPAISPLESWSKYKGVIDARSFLVKGNEINQMMKVVGEEGISNDD B_Borrelia_burgdorferi_VA

12585431 AFHGLSRERSDARRYPAVHPLDSWSKYPSVLDGRSFLRRGAEVEQMMRVVGEEGTSMED B_Treponema_pallidum_VA

**Continued on the next page.**

**GI number Alignment**  **Species names as in the Figure 5**

2605819 RNVVARARRL-ERFLTQP-FFTTEQFTGIKGKSISLSDALDGCERILND B_Methanosarcina_barkeri_F

20091272 RNVVARARRL-ERFLTQP-FFTTEQFTGFKGKFVTLSDALDGCERILLD B_Methanosarcina_acetivorans_F

61219617 KLVVHRARRV-ERFLSQN-THVAKQFTGVDGSDVPLDESIAAFNAICDG B_Streptomyces_coelicolor

15608450 KQLVNRARRI-ERFLSQN-MMAAEQFTGQPGSTVPVKETIEAFDRLCKG B_Mycobacterium_tuberculosis

3913128 KAIVNRARRI-QKFLSQP-FHVAEQFTGMPGKYVKLEDTIRSFKEVLTG B_Aquifex_aeolicus

2493023 KKTVERARKI-EKFLSQP-FFVAEVFTGSPGKYVTLQETLEGFGGILEG B_Helicobacter_pylori

81467688 KVLVARARKI-EKFLSQP-FHVAEVFTGAPGKYVKLADTVRSFKEVISG B_Leptospira_interrogans

3913124 KLVVHRARRI-QRFLSQP-FHVAERFTGRPGRYVPIEETIRGFKEILDG B_Thermotoga_maritima

66826095 KATVFRARKI-QRFLSQP-FEVAHAFTNMEGRFVKLSDSIKAFKGILEG E_Dictyostelium_discoideum_M

53713460 RLVVNRARRV-QRFLSQP-FTVAEQFTGVPGAMVAIEDTIKGFKMILDG B_Bacteroides_fragilis_F

114573 KQVVSRARKI-ERFLTQP-YHVAEVFTGDPGIYVPLKETLRGFKGLLAG B_Vibrio alginolyticus

14547933 KQVVARARKI-ERFLTQP-YHVAEVFTGDPGVYVPLKETLRGFKGLLAG B_Vibrio cholerae

15677764 KLTVMRARKI-QRFLSQP-FHVAEVFTGSPGKYVALRDTIAGFKAILNG B_Neisseria_meningitidis

16131600 KLVVARARKI-QRFLSQP-FFVAEVFTGSPGKYVSLKDTIRGFKGIMEG B_Escherichia_coli

25452812 KLVVARARKV-QRFLSQP-FFVAEAFTGLAGKYVKLEDTIKGFKEIIDG B_Chlorobium_tepidum

81830486 KLTVARARRI-QRFLSQP-FHVAETFTGTPGVYVKLEDTIKGFMGILNG B_Desulfovibrio_vulgaris

81832140 KLVVARARKI-QRFLSQP-FHVAEAFTGSPGKYVELKDTIKGFQEIVAG B_Geobacter_sulfurreducens

22326673 KLTVARARKI-QRFLSQP-FHVAEIFTGAPGKYVDLKENINSFQGLLDG E_Arabidopsis_thaliana_M

114543 KLTVSRARKI-QRFLSQP-FQVAEVFTGHLGKLVPLKETIKGFQQILAG E_Bos_taurus_M

47606749 KLTVARARKI-QRFLSQP-FQVAEVFTGHAGKLVPLEQTIKGFSAILAG E_Drosophila_melanogaster_M

83592562 RLVVARARKI-QRFLSQP-FHVAEVFTGSPGKLVSLEDTIKGFKGLVEG B_Rhodospirillum_rubrum

2493025 KLTVARARKI-QRFLSQP-FDVAKVFTGSDGVQVPLEDTIKSFKAVVAG B_Paracoccus_denitrificans

114570 RLTVDRARKI-ERFLSQP-FFVAEVFTGAPGKYVSLADTIKGFKAILAG B_Synechocystis_PCC_6803

6686269 RLTVARARKI-ERFLSQP-FFVAEVFTGSPGKYVGLAETIRGFNLILSG E_Arabidopsis thaliana_C

11465725 RQTVSRARKI-ERFLSQP-FFVAEVFTGSPGKYVSLEDAIKGFQMILKG E_Porphyra_purpurea_C

1703680 KITVSRARKV-ERLFANH-LMLRNSLPVRWGICKRFGDTIKGFKEILQG B_Acetobacterium_woodii

15673746 KILVGRARRI-QFFLSQN-FHVAEQFTGQPGSYVPIDKTVHDFKEILEG B_Lactococcus_lactis

29377093 KVLVGRARRV-QFFLSQN-FNVAEQFTGQPGSYVPVAETVRGFKEILEG B_Enterococcus_faecalis_F

1168575 KVLVSRARRV-QFFLSQN-FNVAEQFTGLPGSYVPVEETVKGFREILEG B_Enterococcus_hirae_F

114571 KLVVHRARRI-QFFLSQN-FHVAEQFTGQPGSYVPVKETVRGFKEILEG B_Bacillus_PS3

14916961 KLVVHRARRI-QFFLSQN-FHVAEQFTGQKGSYVPVKETIKGFKEILDG B_Bacillus_pseudofirmus

20137589 RLLVGRARRV-QRFLSQA-FSVAEQFTGMKGQFVPVKDTIRSFKEILDG B_Clostridium_acetobutylicum

22266799 KITVSRARKI-ERFFSQP-FHVAEQFTGMEGKYVTVKETITGFKEILEG B_Ilyobacter_tartaricus

1352033 KVTVNRARKI-ERFFSQP-FHVAEQFTGMDGKYVTVKETIRGFKEIIEG B_Propionigenium_modestum

77964181 KLIVARARKI-QRFLSQP-FSVAEQFTGMAGKYVPLKETIRGFREILEG B_Clostridium_paradoxum

2662066 KLTVSRARKI-QRFLSQP-FFVAEQFTGYQGKYVPLKETIRGFKEIIEG B_Ruminococcus_albus

137464 KITLDVATLIKEDFLQQNGYSTYDAFCPIWKTFDMMRAFISYHDEAQKA E_Saccharomyces_cerevisiae_VA

92090652 KITIEVARIIRDDFLQQNGFSPYDKCCPFFKTVWMLKNMMTFYNLAQKA E_Dictyostelium_discoideum_VA

3334404 KITLETAKLLREDYLAQNAFTPYDKFCPFYKSVWMMRNIIHFYNLANQA E_Arabidopsis_thaliana_VA

124053334 KITLEVAKLIKDDFLQQNGYTPYDRFCPFYKTVGMLSNMIAFYDMARRA E_Bos_taurus_VA

93141332 KITLEVAKLLKDDFLQQNSYSSYDRFCPFYKTVGMLRNIIDFYDMARHS E_Drosophila_melanogaster_VA

25009559 KHILNVAFMIREGFLKQDAYNPVDTPSAPIKQFLLMKAIYTYYEEGLKA A_Pyrobaculum_aerophilum_VA

126352990 KHILNVASMIREGFLKQDAFNPVDTPSAPEKQYWLLRLMITYYRVGSEA A_Caldivirga maquilingensis_VA

41614899 KIYLHMGRIIREGFLQQDAFDENDSYSPLEKTIELMRIIHKYYVTVKQL A_Nanoarchaeum_equitans_VA

126008291 KNVLDIAKIIREDFLQQNAFDDTDTYCSIKKQYEMLTIIKTLNEMQEKS A_Ferroplasma_acidarmanus_VA

48477562 KNVLDIAKMIREDFLQQNAFDDIDTYCSIKKQYMMLKIIKTVYEMQMNA A_Picrophilus_torridus_VA

12229704 QLTLEVARYIREAWLQQNALHDVDRYCPPEKTYAILSGIKTLHEESFEA A_Halobacterium salinarium_VA

2493099 QLTLEIARYLREAYLQQNAFHPTDTYCSPEKTYGILTAIHAFNDEAFKA A_Haloferax_volcanii

12585484 RLIIETGRMLRQDFLQQNGFDPVDASASMPKNYGLMKMFLKFYDEAEAA B_Deinococcus_radiodurans_VA

32172455 RLVIEVGRIIREDFLQQNAYHEVDAYCSMKKAYGIMKMILAFYKEAEAA B_Thermus_thermophilus_VA

116754898 QLTLEVARMIINFWLAQSAFHPVDTFCPYKKQYDLLKAILTYRDYAFDA A_Methanosaeta_thermophila_VA

12585404 RVLLEVARIIREVYLIQYAYHPVDTYCSVQKQYDMLKAIKQINDWFYQA A_Archaeoglobus_fulgidus_VA

91773223 QLTLEVARMVREYFLQQNAFHPVDTYCPFDKQYKLLKSITRYGELATAA A_Methanococcoides_burtonii_VA

22002078 QLLLEITRMIREIFLQQNAFHPIDTYSPFEKQYKIMKAIMKWGDAAMDA B_Methanosarcina_mazei_VA

114520 QLLLEITRMLREIFLQQNAFHPVDAYSPFDQQYKILKAIMKWGDAAMDA B_Methanosarcina_barkeri_VA

29376065 RLTLETAKSLREDYLQQNAFDDVDTFTSRTKQAKMLQLILTFGEEGQKA B_Enterococcus_faecalis_VA

1171780 RLTLEVAKSIREDYLQQNAFDDVDTFTSREKQFNMLKVILTFGKEARKA B_Enterococcus hirae_VA

76795559 RLVLEVARSIREDFLHQNAFHEVDTYSSMEKQYRMLKLIMIFYQEAQKA B_Thermoanaerobacter_ethanolicus_VA

110798759 RLKLEVAKSIREDYLQQNAFHENDTYTSLNKQYKMLNLILSFKHEAEKA B_Clostridium_perfringens_VA

87082847 RLTLEAAKSIREDYLHQNAFHEIDTYTSLNKQYKMLKLILEFYHLSQKA B_Caloramator fervidus_VA

12585422 RAILLVARMLREDYLQQDAFDEVDTYCPPEKQVTMMRVLLNFYDKTMEA A_Pyrococcus_horikoshii_VA

57641537 KAILIVTRMLREDYLQQDAFDEVDTYCPPKKQVTMMRVILNFYEKTMQA A_Thermococcus_kodakarensis_VA

12585563 RVILEVARMLREDFLQQDAFDEVDTYCPPMKQYLMLKIIMTFYQEALKA A_Methanocaldococcus_jannaschii_VA

45358607 RVILEIARMLREDFLQQDAYHEVDSYCSPLKQYNMLKIIMTFYKKGLDA A_Methanococcus_maripaludis_VA

84489927 RVTLESARMLREDFLQQNAFDDTDTYCSPSKQYNMLKTILLYNTTAQSA A_Methanosphaera_stadtmanae_VA

12585397 RITLETTRMIREDFLQQNAYHEVDTYCSPSKQFEMLRTIIMFHRNATAA A_Methanothermobacter_thermautotrophicus_VA

118195150 KLILEVARMMKIGLLQQNSFDDVDTYCSPEKQYKLLKMQVDFYKRGQQA A_Cenarchaeum_symbiosum_VA

12585522 KMVLETARLIKDGFLKQNAFDPIDAFATPQKQFRLLKMIMDIHRKSLEL A_Aeropyrum_pernix_VA

74053565 KLILEASKLIRDAFLKQNAFDDIDAFSSPQKQAKIMRLIYDFYTNASQL A_Sulfolobus_acidocaldarius_VA

53714024 HVIFWKSELIDFVILQQDAFDEIDAVTPMERQEAILNMVIDICHTEFEF B_Bacteroides_fragilis_VA

12585442 MEIFLKSELYDFCYLQQNAFDAEDCYCPFDRQIELFSLMNHIFNSRFCF B_Chlamydia_trachomatis_VA

12585416 FLIYLKSELLDSCYLQQNSFDSIDAAVSSERQNYMFDIVYNILKTNFEF B_Borrelia_burgdorferi_VA

12585431 FLVYLKGSFLDSVYLQQNSFDTVDSAVPVARQKHCYAIVMRVLGSVLAF B_Treponema_pallidum_VA
